# Search for Antiviral Preparations in Series of New Derivatives of N-Substituted Piperidines

**DOI:** 10.3390/molecules30122540

**Published:** 2025-06-10

**Authors:** Gulmira S. Akhmetova, Ulzhalgas B. Issayeva, Kaldybay D. Praliyev, Ilya S. Korotetskiy, Tulegen M. Seilkhanov, Samir A. Ross, Manas T. Omyrzakov, Ubaidilla M. Datkhayev, Khaidar S. Tassibekov, Lyudmila N. Ivanova, Natalya V. Zubenko

**Affiliations:** 1 Laboratory of Synthetic and Natural Medicinal Compounds Chemistry, A.B. Bekturov Institute of Chemical Sciences JSC, 106 Sh. Ualikhanov St., Almaty 050010, Kazakhstan; gulgakhmet@mail.ru (G.S.A.); praliyevkd@mail.ru (K.D.P.); kh.tassibekov@ihn.kz (K.S.T.); 2 Scientific Center for Anti-Infectious Drugs JSC, Almaty 050060, Kazakhstan; laeda81@gmail.com (I.S.K.); lyudmila_69.69@mail.ru (L.N.I.); zubenkonatalie@gmail.com (N.V.Z.); 3 International Engineering and Technological University LLC, Almaty 050060, Kazakhstan; 4 Research and Production Association Kazpharmacom LLC, Irgeli 040900, Kazakhstan; 5 The Laboratory of Engineering Profile of NMR Spectroscopy, Sh. Ualikhanov Kokshetau State University, Kokshetau 020000, Kazakhstan; tseilkhanov@mail.ru; 6 National Center for Natural Products Research, School of Pharmacy, The University of Mississippi, Oxford, MS 38677, USA; sross@olemiss.edu; 7 School of Pharmacy, Asfendiyarov Kazakh National Medical University, Almaty 050000, Kazakhstan; u.datkhayev@gmail.com

**Keywords:** piperidines, cyanohydrins, piperidine carboxylic acids, esters, antiviral activity, cytotoxicity

## Abstract

Cyanohydrin synthesis, as the simplest preparative method for introducing a carboxyl group into a piperidine molecule, has been used to obtain potentially biologically active piperidinecarboxylic acids, which have alkyl and arylalkyl radicals at the nitrogen atom of the piperidine ring. Hydrochlorides of cyclopropanecarboxylic acid esters based on piperidinecarboxylic acids, as well as hydrochlorides of fluorobenzoic acid esters of N-substituted piperidines, have been synthesized. The purpose of this study was to search for antiviral drugs among new piperidine derivatives. The structure of the synthesized compounds was studied by NMR methods, including COSY (^1^H-^1^H), HMQC (^1^H-^13^C) and HMBC (^1^H-^13^C) techniques. The values of chemical shifts, multiplicities, and integrated intensities of ^1^H and ^13^C signals in one-dimensional NMR spectra were determined. The results of COSY (^1^H-^1^H), HMQC (^1^H-^13^C), and HMBC (^1^H-^13^C) revealed homo- and heteronuclear interactions, confirming the structure of the studied compounds. The antiviral and cytotoxic activities of the synthesized compounds were studied. The antiviral activity in vitro was determined according to the therapeutic regimen against the influenza A/Swine/Iowa/30 (H1N1) virus on the MDCK cell model. The cytotoxicity of the studied substances in vitro was assessed using the MTT test. Based on the results of the antiviral activity against the influenza A virus, it can be concluded that all substances are effective against the influenza A/H1N1 virus compared to the commercial preparations Tamiflu and Rimantadine.

## 1. Introduction

Influenza is one of the most significant acute infectious diseases in humans, characterized by a high degree of contagiousness and widespread distribution. Among the currently known types of influenza viruses—A, B, and C—type A viruses have the greatest epidemiological importance. They are the primary cause of seasonal epidemics and global pandemics, leading to high morbidity and considerable mortality across various age groups. According to the World Health Organization (WHO, 2018), influenza affects 20–30% of children and 5–10% of adults worldwide each year. Additionally, 3 to 5 million cases of severe illness are reported annually, and influenza-related complications result in an estimated 250,000 to 500,000 deaths globally [1,2,3].

Influenza makes a substantial contribution to morbidity and mortality, affecting all age and social groups. The infection is associated with severe complications, including pneumonia, bronchitis, and involvement of the nervous and cardiovascular systems. Additionally, influenza often exacerbates pre-existing chronic conditions. During epidemic periods, an increase in the incidence of spontaneous miscarriages, preterm births, stillbirths, and congenital malformations in the fetus is observed [4,5,6].

The relevance of combating influenza is largely determined by the unique ability of influenza A viruses to undergo changes in their antigenic structure. The phenomenon, known as antigenic drift, is characterized by gradual alterations in the structure of the virus’s surface proteins—hemagglutinin and neuraminidase—resulting from the accumulation of point mutations. These changes lead to the emergence of new viral strains that are responsible for annual seasonal influenza epidemics. A less frequent but epidemiologically significant process is antigenic shift, which occurs due to the replacement of one or more gene segments. This results in the emergence of viral strains with novel antigenic subtypes, against which the human population has little or no pre-existing immunity. The most likely mechanism for antigenic shift is genetic reassortment, which arises from interactions between human and animal influenza A viruses. The emergence of such reassortant strains may lead to pandemics characterized by high morbidity and substantial mortality [7,8,9,10,11,12].

Influenza pandemics have been recorded over several centuries. The most well-known pandemics occurred in 1918 (the Spanish flu), caused by the A (H1N1) virus; in 1957 (the Asian flu), caused by the A (H2N2) virus; and in 1968 (the Hong Kong flu), caused by the A (H3N2) virus. According to experts, the likelihood of another pandemic remains high, although the exact timing of its onset is uncertain [13,14,15].

Given the growing awareness of the threat of an influenza pandemic, the widespread nature of the disease, the high frequency of complications and fatalities, and the significant socio-economic damage it causes, the problem of combating influenza has become a critical task.

Currently, there are three main strategies for controlling influenza: vaccination, nonspecific chemoprophylaxis, and chemotherapy. Vaccination remains the primary method of preventing influenza, helping to reduce morbidity and mitigate the consequences of annual epidemics.

However, the continuous changes in the influenza virus, such as antigenic drift and antigenic shift, require constant monitoring and the identification of potential epidemic strains. The technological limitations of vaccine production make it difficult to prepare vaccines in sufficient quantities at the onset of an influenza epidemic or pandemic. Moreover, influenza vaccination, unfortunately, has a narrow focus and cannot provide comprehensive protection against all possible viral strains.

On the other hand, vaccination coverage against influenza remains very low in most countries, which also affects its effectiveness. The effectiveness of vaccination is limited, highlighting the need for the development of new preventive approaches and methods.

In this regard, preparations are being developed for both the treatment and the prevention of influenza infection.

The use of chemotherapy preparations is one of the most widespread, simple, and effective methods for the prevention and treatment of viral infections. The search for and creation of effective antiviral agents of direct and indirect action with, possibly, a wider spectrum of activity due to products of synthetic origin remain extremely important tasks in practical healthcare.

In light of the above-mentioned, studies of the anti-influenza activity of new synthetically produced preparations are important and relevant.

In this regard, one of the priority tasks of synthetic organic chemistry is the synthesis of new compounds with an original structure that have a set of predictable properties.

The piperidine ring is a ubiquitous structural feature of many alkaloid natural products and preparation candidates. Thousands of piperidine compounds have been mentioned in clinical and preclinical studies over the past 10 years [16,17,18,19,20,21,22,23]. The diversity of the functional and substitution structures found in piperidine targets has formed the basis for the generally accepted concept that the biological properties of piperidines are highly dependent on the type and location of substituents on the heterocyclic ring.

Numerous studies have confirmed the high biological activity of a number of fluorinated organic compounds; this interest is due to the significant improvement in the pharmacological properties of fluorinated compounds. The introduction of a fluorine atom into the molecules of organic compounds increases their bioavailability, metabolic stability, and lipophilicity and improves the ability of these substances to interact with target proteins [24,25,26,27,28,29,30].

In connection with the above, the purpose of this study was to search for antiviral drugs among new piperidine derivatives.

In continuation from previously initiated studies [31,32], new biologically active piperidine derivatives **(3a-d, 5c, d, 8, 11)** were synthesized within the framework of this work.

## 2. Results and Discussion

### 2.1. Synthesis of New Piperidine Derivatives

**Scheme 1:**
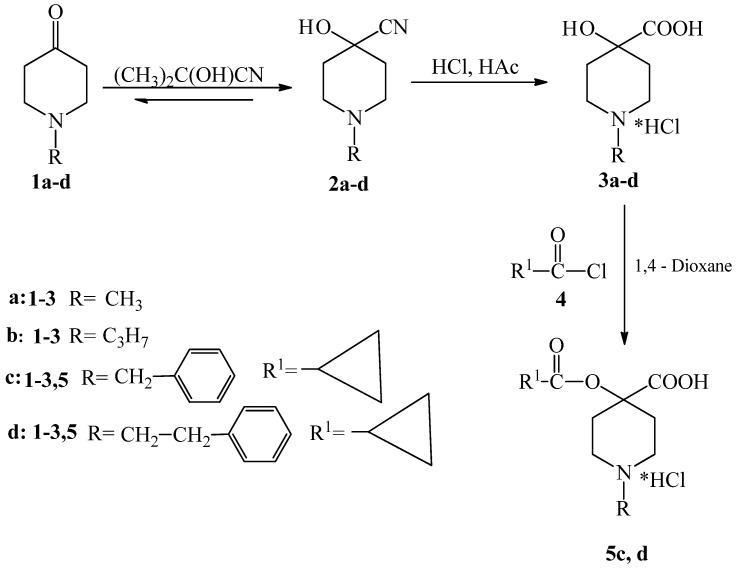
The synthesis of piperidine carboxylic acids **3a-d** and their acyl derivatives **5c, d**.

Crystalline cyanohydrines of [1-methyl, 1-propyl-, 1-benzyl-, 1-(2-phenylethyl-)]-4-ketopiperidines **(2a-d)** with yields of 75–83% were obtained by reacting the corresponding piperidine-4-ones **(1a-d)** with acetone cyanohydrin. The reaction was carried out at room temperature without solvent, with the addition of a small amount (2–3 drops) of water. Further, in order to obtain piperidine carboxylic acids, their acid hydrolysis was carried out to form hydrochlorides [1-methyl, 1-propyl-, 1-benzyl-, 1-(2-phenylethyl-)]-4-hydroxy-4-carboxypiperidines **(3a-d)**. The cyanohydrin hydrolysis was carried out with concentrated hydrochloric acid at room temperature (Scheme 1).

In order to study the reactivity of the hydroxyl group and subsequently clarify the effect of the nature of the acyl residue—particularly regarding the introduction of a cyclopropane fragment into the piperidine structures—on pharmacological properties, cyclopropanecarboxylic acid ester **(5c, d)** hydrochlorides were synthesized by the hydroxyl group of hydrochlorides [1-benzyl-,1-(2-phenylethyl-)]-4-hydroxy-4-carboxypiperidines **(3c, d)**.

By the acylation of [1-benzyl-,1-(2-phenylethyl-)]-4-hydroxy-4-carboxypiperidine hydrochlorides **(3c-d)** with cyclopropanecarboxylic acid, chlorangydride hydrochlorides of cyclopropanecarboxylic acid esters **(5c-d)** were synthesized.

In the IR spectra of cyanohydrins **(2a-d)**, the characteristic absorption bands of the nitrile group (2230–2802 cm^−1^) and the hydroxyl group in the range of 3420–3468 cm^−1^ were observed. In the IR spectra of hydroxyacids **(3a-d)**, there were characteristic absorption bands of carbonyls of the carboxyl group in the range of 1723–1741 cm^−1^ and the hydroxyl group in the range of 3374–3472 cm^−1^. In the IR spectra of the esters obtained **(5c, d)**, there were intense absorption bands of valence vibrations of ester carbonyls in the range of 1726–1735 cm^−1^ and carbonyls of the carboxyl (acid) group in the range of 1421–1468 cm^−1^, as well as the carboxyl hydroxyl at 3400–3600 cm^−1^.

The structures of the synthesized compounds **(3b, 5c, 5d)** were studied by ^1^H and ^13^C NMR methods, including the COSY (^1^H-^1^H), HMQC (^1^H-^13^C), and HMBC (^1^H-^13^C) techniques. The values of the chemical shifts, multiplicity, and integral intensity of ^1^H and ^13^C signals in one-dimensional NMR spectra were determined. The results of COSY (^1^H-^1^H), HMQC (^1^H-^13^C), and HMBC (^1^H-^13^C) revealed homo- and heteronuclear interactions, confirming the structure of the studied compounds (Table S1 in Supplementary Materials).

The detailed ^1^H and ^13^C NMR spectra of the compounds (**3a-d)** and (**5c, d)** are provided in the Supplementary Materials (Figures S13–S35).

In the ^1^H-^1^H COSY spectra of the compound **3b**, spin–spin correlations were observed through three proton bonds of neighboring methyl and methylene groups H^9^-H^8^ (0.81, 1.65 and 1.65, 0.81) and H^8^-H^7^ (1.74, 2.23 and 2.23, 1.74) of the N-propyl fragment and methylene groups H^3ax,5ax^-H^3eq,5eq^ (1.74, 2.23 and 2.23, 1.74), H^3ax,5ax^-H^2ax,6ax^ (1.67, 2.89 and 2.89, 1.67), and H^2ax,6ax^-H^2eq,6eq^ (2.91, 3.27 and 3.27, 2.91) of the piperidine ring. The heteronuclear interactions of protons with carbon atoms through a single bond were established, using ^1^H-^13^C HMQC spectroscopy for the following pairs present in the compound: H^9^-C^9^ (0.82, 11.57), H^8^-C^8^ (1.66, 17.22), H^3ax,5ax^-C^3,5^ (1.70, 31.17), H^3eq,5eq^-C^3,5^ (2.19, 31.17), H^2ax,6ax^-C^2,6^ (2.92, 47.45), H^2eq,6eq^-C^2,6^ (3.29, 47.45), and H^7^-C^7^ (2.91, 57.75).

The correlations observed in the compound **5c** molecule were shown, which made it possible to establish spin–spin interactions of a homo- and heteronuclear nature.

In the ^1^H–^1^H COSY spectra of compound **5c**, spin–spin correlations through three bonds were observed between the protons of adjacent methylene and methine groups: H^20,21^-H^19^ (0.88, 1.65 and 1.65, 0.88) of the cyclopropane fragment; the adjacent methylene groups H^3ax,5ax^-H^3eq,5eq^ (2.17, 2.39 and 2.39, 2.17) of the piperidine ring; methine–methine groups H^9,11,13^-H^10,12^ (3.06, 3.18 and 3.18, 3.06) of the N-ethylphenyl fragment; and methine–methine protons H^10−14^-H^10−14^ (7.38, 7.65 and 7.65, 7.38) of the aromatic ring. The heteronuclear one-bond interactions between the protons and carbon atoms were determined, using the ^1^H–^13^C HMQC spectroscopy for the following pairs present in the compound: H^20,21^-C^20,21^ (0.87, 9.25), H^19^-C^19^ (1.64, 13.23), H^3ax,5ax^-C^3,5^ (2.17, 28.87), H^3eq,5eq^-C^3,5^ (2.43, 28.85), H^7^-C^7^ (4.30, 59.05), H^9,11,13^-C^9,11,13^ (7.38, 128.18), and H^7^-C^7^ (3.21, 57.03).

In the ^1^H–^1^H COSY spectra of compound **5d**, spin–spin correlations through three bonds were observed between the protons of the adjacent methylene and methine groups: H^21,22^-H^20^ (0.92, 1.73 and 1.73, 0.82) of the cyclopropane fragment; the adjacent methylene groups H^3ax,5ax^-H^3eq,5eq^ (2.18, 2.39 and 2.39, 2.18) and H^2ax,6ax^-H^2eq,6eq^ (3.02, 3.50 and 3.50, 3.02) of the piperidine ring; and the methylene–methylene groups H^8^-H^7^ (3.06, 3.25 and 3.25, 3.06) of the N-ethylphenyl fragment. The one-bond heteronuclear interactions between the protons and carbon atoms were established by means of ^1^H–^13^C HMQC spectroscopy for the following pairs present in the compound: H^21,22^-C^21,22^ (0.87, 9.25), H^3ax,5ax^-C^3,5^ (2.21, 29.18), H^3eq,5eq^-C^3,5^ (2.43, 29.18), H^8^-C^8^ (3.05, 56.75), H^2eq,6eq^-C^2,6^ (3.53, 47.12), and H^9−13^-C^9−13^ (7.23, 129.18).

**Scheme 2:**
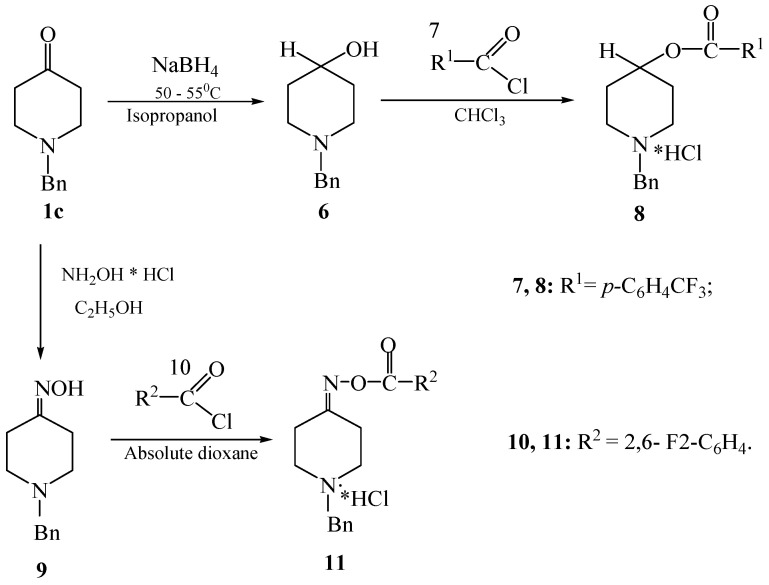
The synthesis of fluorinated piperidine derivatives: compounds **8** and **11**.

Based on 1-benzylpiperidin-4-one **(1c)**, 1-benzyl-4-hydroxypiperidine **(6)** was synthesized in good yield via the reduction with sodium borohydride in isopropanol. The reaction of hydrochloride hydroxylamine with 1-benzyl-4-oxopiperidine **(1c)** in the presence of base in ethanol yielded the oxime of 1-benzylpiperidin-4-one **(9**) (Scheme 2).

In order to obtain new fluorine-containing derivatives of piperidines, the compound **6** (alcohol) was acylated with 4-(trifluoromethyl)benzoyl chloride **(7)**, while oxime (**9)** was acylated with 2,6-difluorobenzoyl chloride **(10)**, affording the corresponding hydrochlorides of fluorobenzoate esters **(8, 11)** (Scheme 2).

The acylation reaction was carried out at room temperature or under heating in dioxane or chloroform by treating the starting piperidinol **(6)** and piperidone oxime **(9)** with an excess of fluorobenzoic acid chlorides. The resulting esters were crystalline powders, ranging in color from white to cream, readily soluble in water, ethanol, and acetone. Their composition and structure were confirmed by elemental analysis and IR and NMR spectroscopy, and their purity was verified by thin-layer chromatography (Al_2_O_3_, eluent benzene/dioxane 3:2, Rf = 0.82–0.90).

In the IR spectra, intense absorption bands at 1718–1730 cm^−1^, corresponding to C=O stretching vibrations of the ester group, confirmed the formation of hydrochloride salts of the target esters **(8, 11)**.

The spectra of esters **(8, 11)** were characterized by strong singlet signals of the ester carbonyl carbon atoms at 1723.1 cm^−1^ and 1754.0 cm^−1^, respectively. Additionally, the signals corresponding to the carbon atoms of the aromatic rings were observed.

The structures of the synthesized compounds **(8, 11)** were studied by ^1^H and ^13^C NMR spectroscopy, including 2D techniques such as COSY (^1^H–^1^H), HMQC (^1^H–^13^C), and HMBC (^1^H–^13^C). The chemical shift values, multiplicities, and integral intensities of the ^1^H and ^13^C signals were determined from the one-dimensional NMR spectra. The results of the COSY (^1^H–^1^H), HMQC (^1^H–^13^C), and HMBC (^1^H–^13^C) experiments revealed homo- and heteronuclear interactions, confirming the structures of the investigated compounds.

The detailed ^1^H and ^13^C NMR spectra of the compounds **(8, 11)** are provided in the Supplementary Materials (Figures S11, S12, and S36–S42).

The structure of the compounds **(8, 11)** was confirmed by the two-dimensional NMR spectroscopy, using the COSY (^1^H–^1^H) and HMQC (^1^H–^13^C) techniques, which enabled the identification of the homo- and heteronuclear spin–spin interactions (Table S1 in the Supplementary Materials).

In the ^1^H–^1^H COSY spectra of compound **(11),** spin–spin correlations through three bonds were observed between the protons of the adjacent methine groups H^11^–H^10,12^ (7.27, 7.66 and 7.66, 7.27) and H^20^–H^21^ (7.40, 7.60 and 7.60, 7.40) of the aromatic rings. The one-bond heteronuclear interactions between the protons and carbon atoms were identified, using ^1^H–^13^C HMQC spectroscopy for the following pairs present in the compound: H^3,5^–C^3,5^ (2.94, 23.86), H^2,6^–C^2,6^ (3.40, 49.11), H^7^–C^7^ (3.20, 50.11), H^11,22^–C^11,22^ (7.25, 113.24), H ^10,12,20^–C^10,12,20^ (7.39, 129.18), and H^9,13,21^–C^9,13,21^ (7.67, 135.16).

### 2.2. The Determination of Cytotoxicity of the Investigational Drugs In Vitro

Drug safety is a key aspect in the study of the properties of drugs under development. In order to determine the maximum concentrations that did not have toxic effects, the cytotoxic activity of five hetero-organic derivatives **(3b, 5c, 5d, 8, 11)**, as well as reference compounds rimantadine and oseltamivir (Tamiflu), was studied.

**Figure 1:**
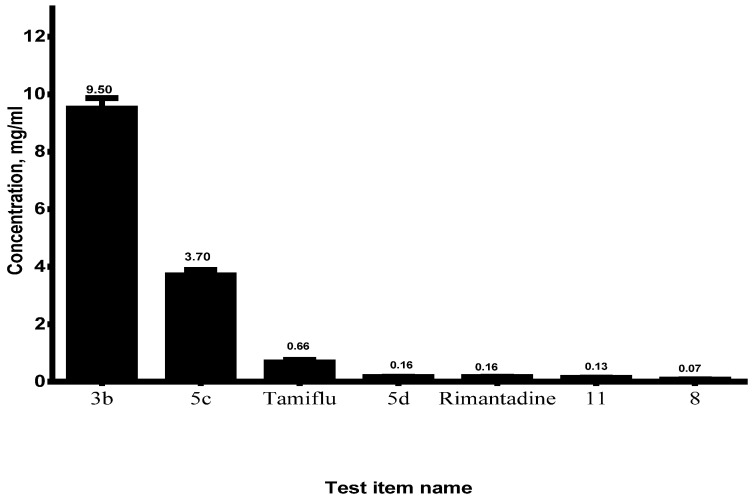
Toxicity series for substances against the MDCK cell culture.

The quantitative assessment of cytotoxicity of the studied substances was performed using the MTT test. The data were recorded 72 h after exposure to the studied compounds. Based on the results obtained, the CC_50_ values were calculated (presented in Table 1 and Figure 1).

### 2.3. The Study of the Antiviral Activity of the Investigated Substances in the In Vitro Experiments

The antiviral activity of the tested compounds was evaluated on the influenza A/H1N1 virus model. Since the reference drugs rimantadine and oseltamivir (Tamiflu) are mainly used in therapeutic regimens, the hetero-organic derivatives were also tested according to the scheme of therapeutic administration.

The MDCK cell culture was infected with the influenza A/Swine/Iowa/30 (H1N1) virus at a dose of 100 ID/0.2 mL and then the test substances were added in a series of dilutions. Activity was determined at six consecutive twofold dilutions, starting from a concentration of 1/2 CC_50_. The efficacy of the compounds was evaluated by decreasing the virus infectivity titer by hemagglutination. A comparative evaluation of activity was performed for compound **5c** with respect to rimantadine (Table 2).

**Figure 2:**
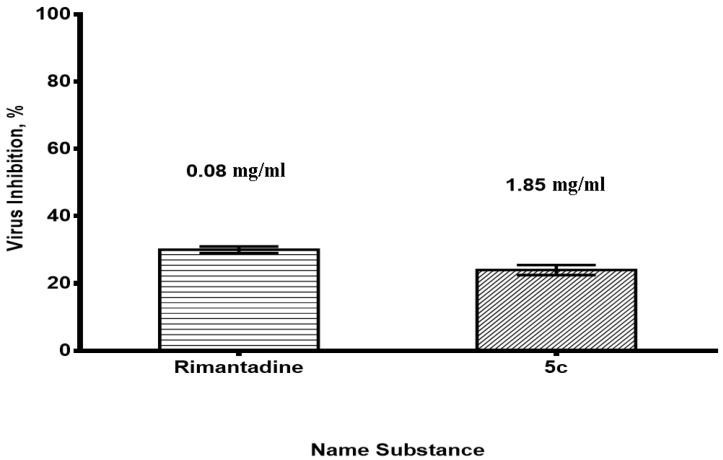
The comparative therapeutic activity of rimantadine and heterorganic compound **5c**.

The results of the experiments showed that compound **5c** suppressed the replication of the influenza A/Swine/Iowa/30 (H1N1) virus by 1.2 log_2_ infectious doses compared to the control group. However, this effect was observed exclusively at the maximum investigated concentration of the drug (1.85 mg/mL). IC_50_ value calculation was not performed because none of the tested compounds in the selected range demonstrated the suppression of virus replication by 50% or more. No statistically significant antiviral effect of **5c** was detected when using the other concentrations tested (Figure 2).

The analysis of the experimental data (Figure 2) showed that heterorganic compound **5c** was characterized by a level of antiviral activity comparable to that of rimantadine at concentrations equivalent to 1/2 of the cytotoxic concentration (CC_50_).

A comparative characterization of the antiviral activity of compounds **3b, 5d, 8,** and **11** was performed, using Tamiflu as a reference drug (Table 3).

The IC_50_ value could not be calculated because none of the tested compounds suppressed virus replication by 50% or more.

This study found that compound **11** demonstrated the ability to inhibit the replication of 100 infectious doses of the influenza A/H1N1 virus by 2.0 log_2_ compared to the control group at the concentrations of 0.0650 and 0.0325 mg/mL. Compound **8** reduced viral load by 2.0 log_2_ at a concentration of 0.035 mg/mL and by 1.0 log_2_ at the concentrations of 0.0175 and 0.0088 mg/mL. Compound **3b** exhibited antiviral activity, reducing virus replication by 1.0 log_2_ exclusively at the maximum tested concentration of 4.75 mg/mL. **5d** demonstrated pronounced antiviral action: at a concentration of 0.08 mg/mL, the pressure was 2.0 log_2_, and at 0.04 mg/mL, it was 1.2 log_2_.

**Figure 3:**
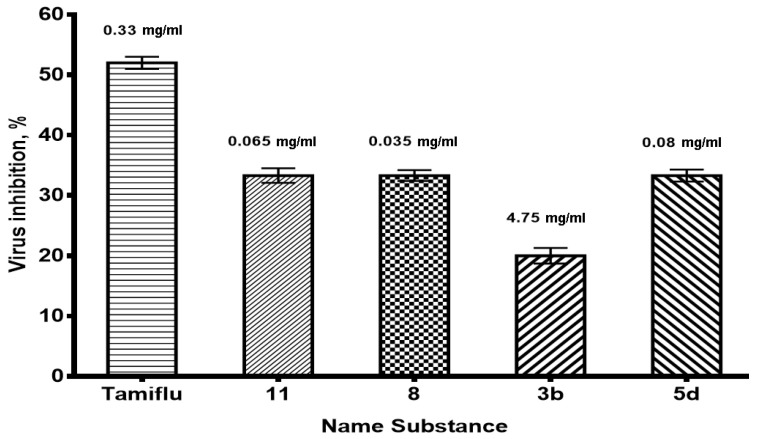
The comparative therapeutic activity of Tamiflu and heterorganic compounds **3b, 5d, 8,** and **11**.

In comparison, the reference drug oseltamivir (Tamiflu) at concentrations of 0.33 and 0.17 mg/mL provided a 2.6 log_2_ reduction in the reproduction of 100 infectious doses of the virus. At concentrations of 0.08 and 0.04 mg/mL, oseltamivir reduced the viral load by 2.0 log_2_, whereas, at concentrations of 0.02 and 0.01 mg/mL, a reduction of 1.0 log_2_ was observed (Figure 3).

Based on the data presented in Figure 3, the anti-viral activity of heterorganic compounds **3b, 5d, 8,** and **11** at concentrations corresponding to 1/2 of their CC_50_ was slightly inferior to the therapeutic efficacy of the comparison drug oseltamivir (Tamiflu). Compounds **5d, 8,** and **11** demonstrated a comparable level of inhibition of influenza A/H1N1 virus replication at the maximum concentration tested (1/2 of CC_50_). At the same time, compound **3b** exhibited significantly lower antiviral activity against the influenza A/H1N1 virus compared to Tamiflu.

A comparative analysis of the obtained data indicated the anti-viral activity of heterorganic compounds **3b, 5d, 8,** and **11** against the influenza A/H1N1 virus. In particular, compounds **8** and **11** showed comparable efficacy to commercially available oseltamivir at similar or lower concentrations. These results support the further investigation of these compounds as potential antiviral agents against the influenza A/H1N1 virus.

## 3. Materials and Methods

### 3.1. Chemical Experimental Part

#### 3.1.1. Reagents and Equipment

Piperidin-4-ones **(1a-d)** and fluorobenzoyl chlorides and cyclopropanecarbonyl chloride were purchased from Sigma-Aldrich (Louis Street, MO, USA). The IR spectra were recorded on a Nicolet 5700 instrument between KBr plates. The ^1^H and ^13^C NMR spectra were recorded on a JNM-ECA Jeol 400 spectrometer (frequencies 399.78 and 100.53 MHz, respectively) using DMSO-*d*_6_ solvent. The elemental analysis data were consistent with the calculated values. The column chromatography and thin-layer chromatography were carried out on alumina (Al_2_O_3_) of the third degree of activity, and Rf compounds were given for this type of plate. The spots were developed in iodine vapors. The IR and NMR spectra for the synthesized compounds are presented in the Supplementary File.

#### 3.1.2. Synthesis of Piperidine Carboxylic Acids (**3a–d**)

A solution of 3.0 g (0.0167 mol) cyanohydrin piperidine-4-one in 35 mL of concentrated hydrochloric acid and 17 mL of acetic acid was kept at room temperature for seven days. Then, it was alkalized with a concentrated sodium hydroxide solution to pH = 10 and extracted with benzene. The aqueous layer was acidified to pH = 1, evaporated dry at a temperature of 55–600 °C, and treated with acetone and then isopropanol.

Hydrochloride of 1-methyl-4-carboxy-4-hydroxypiperidine (**3a**). White powder, yield 79%, melting point 170–172 °C. IR spectrum (KBr), ν, cm^−1^: 1723.6 (C=O). ^1^H NMR spectrum (DMSO-*d*_6_), δ, ppm (*J*, Hz): 1.73 d (2H, H^3_ax^, H^5_ax^, ^3^*J* = 13.6 Hz), 2.12 t (2H, H^3_eq^, H^5_eq^, ^3^*J* = 12.6 Hz), 2.64 s (3H, H^7,7,7^), 3.00 t (2H, H^2_ax^, H^6_ax^, ^3^*J* = 12.6 Hz), 3.20 d (2H, H^2_eq^, H^6_eq^, ^3^*J* = 10.4 Hz), 5.70 br s (1H, H^8^), 7.30–7.56 (1H, H^12^), 10.84 br s (1H, H^11^).^13^C NMR spectrum (DMSO-*d*_6_), δC, ppm: 31.41 (C^3,5^), 42.87 (C^7^), 49.27 (C^2,6^), 68.43 (C^4^), 176.31 (C^9^). COSY NMR spectrum (DMSO-*d*_6_), δC, ppm: H^3ax,5ax^ → H^3eq,5eq^, H^2ax,6ax^ → H^2eq,6eq^, H^17^ → H^14^. HMQC NMR spectrum: H^3ax,5ax^ → C^3,5^, H^3eq,5eq^ → C^3,5^, H^7^ → C^7^, H^2ax,6ax^ → C^2,6^, H^2eq,6eq^ → C^2,6^. Found, %: C 44.16; H 8.25; N 7.63. C_7_H_14_NO_3_Cl. Calculated, %: C 43.06; H 7.17; N 7.17.

Hydrochloride of 1-propyl-4-carboxy-4-hydroxypiperidine (**3b**). White powder, yield 70%, melting point 173–175 °C. IR spectrum (KBr), ν, cm^−1^: 1741.8 (C=O). ^1^H NMR spectrum (DMSO-*d*_6_), δ, ppm (*J*, Hz): 0.82 t (3H, H^9,9,9^, ^3^*J* 7.4), 1.66–1.76 m (4H, H^8,8,3ax,5ax^), 2.19 t (2H, H^3eq,5eq^, ^3^*J* 13.6), 2.64 s (3H, H^7,7,7^), 2.88–2.95 m (4H, H^7,7,2ax,6ax^), 3.28 d (2H, H^2eq,6eq^, ^3^*J* 10.8), 5.66 br s (1H, H^10^), 7.28–7.53 m (1H, H^14^), 10.78 br s (1H, H^13^). ^13^C NMR spectrum (DMSO-*d*_6_), δC, ppm: 11.52 (C^9^), 17.14 (C^8^), 31.27 (C^3,5^), 47.60 (C^2,6^), 57.80 (C^7^), 68.89 (C^4^), 176.24 (C^11^). COSY NMR spectrum (DMSO-*d*_6_), δC, ppm: H^9^ → H^8^, H^8^ → H^7^, H^3ax,5ax^ → H^3eq,5eq^, H^3ax,5ax^ → H^2ax,6ax^, H^2ax,6ax^ → H^2eq,6eq^. HMQC NMR spectrum: H^9^ → C^9^, H^8^ → C^8^, H^3ax,5ax^ → C^3,5^, H^3eq,5eq^ → C^3,5^, H^7^ → C^7^, H^2ax,6ax^ → C^2,6^, H^2eq,6eq^ → C^2,6^. Found, %: C 49.38; H 8.96; N 7.32. C_9_H_18_ClNO_3_. Calculated, %: C 48.41; H 8.06; N 6.27.

Hydrochloride of 1-benzyl-4-carboxy-4-hydroxypiperidine (**3c**). Thick brown oil, yield 63%. IR spectrum (KBr), ν, cm^−1^: 1741.9 (C=O). ^1^H NMR spectrum (DMSO-*d*_6_), δ, ppm (*J*, Hz): 1.74 d (2H, H^3ax,5ax^, ^3^*J* 13.5), 2.20 t (2H, H^3eq,5eq^, ^3^*J* 13.5), 3.00 k (2H, H^2ax,6ax^, ^3^*J* 10.8), 3.14 d (2H, H^2eq,6eq^, ^3^*J* 10.8), 4.23 d (2H, H^7,7^, ^4^*J* 4.0), 7.36 s (3H, H^9,11,13^), 7.59 s (2H, H^10,12^), 5.39 br s (1H, H^14^), 10.97 br s (1H, H^17^). ^13^C NMR spectrum (DMSO-*d*_6_), δC, ppm: 31.15 (C^3,5^), 47.17 (C^2,6^), 68.85 (C^4^), 59.19 (C^7^), 129.22 (C^9,11,13^), 130.05 (C^8^), 132.13 (C^10,12^), 176.23 (C^15^). COSY NMR spectrum (DMSO-*d*_6_), δC, ppm: H^3ax,5ax^ → H^3eq,5eq^, H^2ax,6ax^ → H^2eq,6eq^, H^9,11,13^ → H^10,12^. Found, %: C 58.61; H 7.14; N 6.39. C_13_H_18_ClNO_3_. Calculated, %: C 57.74; H 6.63; N 5.16.

Hydrochloride of 1-(2-phenylethyl)-4-carboxy-4-hydroxypiperidine (**3d**). White powder, yield 88%, melting point 160–162 °C. IR spectrum (KBr), ν, cm^−1^: 1734.9 (C=O). ^1^H NMR spectrum (DMSO-*d*_6_), δ, ppm (*J*, Hz): 1.79 d (2H, H^3ax,5ax^, ^3^*J* 13.6), 2.24 t (2H, H^3eq,5eq^, ^3^*J* 12.6), 3.06 s (4H, H^8,8,2ax,6ax^), 3.40 d (2H, H^2eq,6eq^, ^3^*J* 10.0), 3.20 t (2H, H^7,7^, ^3^*J* 7.2), 7.18–7.28 m (5H, H^10−14^), 5.72 br s (1H, H^15^), 7.28–7.57 m (1H, H^19^), 11.10 br s (1H, H^18^). ^13^C NMR spectrum (DMSO-*d*_6_), δC, ppm: 29.84 (C^8^), 31.38 (C^3,5^), 47.69 (C^2,6^), 57.03 (C^7^), 68.92 (C^4^), 127.26 (C^12^), 129.14 (C^10−14^), 137.14 (C^9^), 176.28 (C^16^). COSY NMR spectrum (DMSO-*d*_6_), δC, ppm: H^3ax,5ax^ → H^3eq,5eq^, H^3eq,5eq^ → C^3,5^, H^2ax,6ax^ → H^2eq,6eq^, H^8^ → H^7^, H^3ax,5ax^ → H^2ax,6ax^. HMQC NMR spectrum: H^3ax,5ax^ → C^3,5^, H^3eq,5eq^ → C^3,5^, H^2ax,6ax^ → C^2,6^, H^2eq,6eq^ → C^2,6^, H^8^ → C^8^, H^7^ → C^7^. Found, %: C 59.67; H 7.92; N 5.24. C_14_H_20_NO_3_Cl. Calculated, %: C 58.84; H 7.00; N 4.90.

#### 3.1.3. Synthesis of Piperidine-Containing Esters of Cyclopropanecarboxylic Acid (**5c–d**)

A solution of (0.022 mol) cyclopropancarbonyl chloride was added to the mixture (0.007 mol) of 4-carboxy-4-hydroxypiperidine hydrochloride while stirring. At the same time, warming up was observed, and a white precipitate fell out. The mixture was kept for 12 h at room temperature. The precipitate was washed with diethyl ether; the remainder was recrystallized from acetone.

Hydrochloride of 1-benzyl-4-cyclopropanoyloxy-piperidine-4-carboxylic acid (**5c**). Crystalline product, yield 59%, melting point 208–210 °C. IR spectrum (KBr), ν, cm^−1^: 1730.2 (C=O) ester, 1421.8 (C=O) carboxylic. ^1^H NMR spectrum (DMSO-*d*_6_), δ, ppm (*J*, Hz): 0.81–0.84 m (4H, H^20ax,21ax,20eq,21eq^), 1.66 s (1H, H^19^), 2.17 d (2H, H^3ax,5ax^, ^3^*J* 13.6), 239 t (2H, H^3eq,5eq^, ^3^*J* 11.6), 3.01 s (2H, H^2ax,6ax^), 3.19 d (2H, H^2eq,6eq^), 4.30 s (1H, H^7,7^), 7.38 s (3H, H^9,11,13^), 7.65 s (2H, H^10,12^), 11.45 br s (1H, H^22^). ^13^C NMR spectrum (DMSO-*d*_6_), δC, ppm: 9.09 (C^20,21^), 13.32 (C^19^), 29.02 (C^3,5^), 47.10 (C^2,6^), 58.96 (C^7^), 75.36 (C^4^), 129.19 (C^9,13^), 129.92 (C^11^), 130.26 (C^8^), 132.06 (C^10,12^), 172.04 (C^17^), 173.30 (C^15^). COSY NMR spectrum (DMSO-*d*_6_), δC, ppm: H^20,21^ → H^19^, H^9,11,13^ → H^10,12^, H^3ax,5ax^ → H^3eq,5eq^. HMQC NMR spectrum: H^20,21^ → C^20,21^, H^19^ → C^19^, H^3ax,5ax^ → C^3,5^, H^3eq,5eq^ → C^3,5^, H^7^ → C^7^, H^9,11,13^ → C^9,11,13^. Found, %: C 61.33; H 7.52; N 5.04; C_17_H_22_ClNO_4_. Calculated, %: C 60.15; H 6.48; N 4.12.

Hydrochloride of 1-(2-phenylethyl)-4-cyclopropanoyloxy-piperidine-4-carboxylic acid (**5d**). Crystalline product, yield 60%, melting point 167–169 °C. IR spectrum (KBr), ν, cm^−1^: 1717.9 (C=O) ester, 1468.3 (C=O) carboxylic. ^1^H NMR spectrum (DMSO-*d*_6_), δ, ppm (*J*, Hz): 0.79–0.93 m (4H, H^21ax,22ax,21eq,22eq^), 1.73 s (1H, H^20^), 2.19 d (2H, H^3ax,5ax^, ^3^*J* 13.8), 2.39 t (2H, H^3eq,5eq^, ^3^*J* 12.6), 3.06 br s (2H, H^8,8,2ax,6ax^), 3.28 s (1H, H^7,7^), 3.52 s (2H, H^2eq,6eq^), 4.84–4.87 m (1H, H^24^), 7.21–7.29 m (5H, H^10−14^), 11.45 br s (1H, H^23^). ^13^C NMR spectrum (DMSO-*d*_6_), δC, ppm: 9.14 (C^21,22^), 13.17 (C^20^), 29.13 (C^3,5^), 47.27 (C^2,6^), 56.79 (C^8^), 69.32 (C^7^), 75.26 (C^4^), 127.31 (C^12^), 129.19 (C^9,10,11,13^), 137.65 (C^9^), 169.93 (C^18^), 172.01 and 173.47 (C^16^). COSY NMR spectrum (DMSO-*d*_6_), δC, ppm: H^21,22^ → H^20^, H^3ax,5ax^ → H^3eq,5eq^, H^2ax,6ax^ → H^2eq,6eq^. HMQC NMR spectrum: H^21,22^ → C^21,22^, H^19^ → C^19^, H^3ax,5ax^ → C^3,5^, H^3eq,5eq^ → C^3,5^, H^8^ → C^8^. Found, %: C 62.36; H 7.53; N 4.27; C_18_H_24_ClNO_4_. Calculated, %: C 61.16; H 6.79; N 3.96.

#### 3.1.4. Synthesis of Piperidine-Containing Esters of Fluorobenzoic Acids (**8**, **11**)

A solution of (0.019 mol) fluorobenzoyl chloride in chloroform or absolute dioxane was added to a solution of (0.013 mol) alcohol **(6)** or ketoxime **(9)** in chloroform while stirring. In this case, heating and discoloration of the reaction mixture were observed. The mixture was kept for 12 h at room temperature. The precipitated white precipitate was filtered out, washed with diethyl ether, and the remainder was recrystallized from isopropyl alcohol.

Hydrochloride of 1-benzyl-4-trifluoromethylbenzoyloxy-piperidine (**8**). White powder, yield 54%, melting point 200–202 °C. IR spectrum (KBr), ν, cm^−1^: 1723.1 (C=O). ^1^H NMR spectrum (DMSO-*d*_6_), δ, ppm (*J*, Hz): 2.03–2.31 m (4H, H^3ax,5ax,3eq,5eq^), 3.09–3.36 m (4H, H^2ax,6ax,2eq,6eq^), 5.05–5.24 m (1H, H^4^), 4.7–4.36 m (2H, H^7,7^), 7.40–7.41 m (3H, H^10,11,12^), 7.61–7.68 m (2H, H^13,19^), 7.85–7.88 m (2H, H^19,21^), 8.11–8.25 m (2H, H^18,22^). ^13^C NMR spectrum (DMSO-*d*_6_), δC, ppm: 26.86 and 27.79 (C^3,5^), 47.18 and 49.57 (C^2,6^), 66.45 and 68.85 (C^4^), 58.61 and 59.19 (C^7^), 122.89 and 125.60 (C-23), 126.32 (C^19,21^), 129.25 (C^9,13^), 129.93 (C^11^), 130.43 (C^8^), 130.68 (C^18,22^), 131.99 (C^10,12^), 133.61 and 134.03 (C^20^), 133.30 (C^17^), 164.44 (C^15^). Found, %: C 61.54; H 6.02; N 4.36; C_20_H_21_ClF_3_NO_2_. Calculated, %: C 60.02; H 5.25; N 3.50.

Hydrochloride of 1-benzyl-4-(2,6-difluoro-benzoyloxymino) piperidine (**11**). White powder, yield 94%, melting point 162–164 °C. IR spectrum (KBr), ν, cm^−1^: 1754.0 (C=O), 1619.7 (C=N). ^1^H NMR spectrum (DMSO-*d*_6_), δ, ppm (*J*, Hz): 2.75–3.34 m (8H, H^2ax,6ax,2eq,6eq,3ax,5ax,3eq,5eq^), 4.34 s (2H, H^7,7^), 7.24–7.28 m (2H, H^21,22^), 7.39–7.41 m (3H, H^10,12,20^), 7.61–7.71 m (2H, H^9,13,21^), 11.89 br s (1H, H^26^). ^13^C NMR spectrum (DMSO-*d*_6_), δC, ppm: 23.95 and 27.71 (C^3,5^), 49.07 and 50.11 (C^2,6^), 29.13 (C^3,5^), 47.27 (C^2,6^), 58.62 (C^7^), 69.32 (C^7^), 108.94, 109.13 and 109.32 (C^18^), 113.13 and 113.38 (C^20,22^), 129.31 (C^10,12^), 130.01 (C^11^), 130.25 (C^8^), 131.88 (C^9,13^), 135.24, 135.35 and 135.45 (C^21^), 158.94 and 161.48 (C^19,23^), 158.57 (C^16^), 163.86 (C^4^). COSY NMR spectrum (DMSO-*d*_6_), δC, ppm: H^11^ → H^10,12^, H^20^ → H^21^. HMQC NMR spectrum: H^3,5^ → C^3,5^, H^2,6^ → C^2,6^, H^7^ → C^7^, H^11^ → C^11^, H^22^ → C^22^, H^10,12^ → C^10,12^, H^9,13^ → C^9,13^. Found, %: C 60.31; H 5.22; N 8.61. C_19_H_19_N_2_O_2_F_2_Cl. Calculated, %: C 59.98; H 4.99; N 7.36.

### 3.2. Biological Experimental Part

*The objects of this research*

The objects of this study were new piperidine derivatives: compounds **3b, 5c, 5d, 8,** and **11**.

- RIMANTADIN-STI, manufactured by Irbit Chemical and Pharmaceutical Plant OJSC, Moscow, Russia.
- Tamiflu, Roche produced by Cenexi S.a.S, Osny, France.

### 3.3. The Cell Culture and Virus

The monolayer transient cell culture MDCK (Madin Darby Canine Kidney cells) obtained from the Laboratory of Cell Biotechnology of the Research Institute of Biological Safety Problems at the National Center of Biotechnology of the Ministry of Education and Science of the Republic of Kazakhstan was used.

Influenza virus strain A/Swine/Iowa/30 (H1N1), obtained from the ATCC collection, USA, was used. The virus was propagated in the MDCK cell culture for 72 h at 35 °C. The virus titer in the culture fluid was 10^7.7^ TCD50/mL.

The infectious titer of the influenza virus was determined by titration on the MDCK cell culture by limiting dilutions. The presence of the influenza virus was judged by the hemagglutination reaction. The virus infectivity titer was calculated according to the method of Reed and Muench [33]. The hemagglutinating activity of viruses was determined using a 0.75% suspension of human erythrocytes (group 1). [34].

### 3.4. Determination of Cytotoxicity of Substances In Vitro

The cytotoxicity of the investigated substances in vitro was assessed, using the MTT test [35]. The plates were incubated in the thermostat at 37 °C and 5.0% CO_2_. After 72 h, optical density was recorded on a Tecan Sunrise RC.4 microplate reader (Austria) at a wavelength of 540 nm for the main filter and 620 nm for the reference filter. The CC50 value was calculated using the following Equation (1):

(1) $${\mathrm{CC}}_{50}=\left[\right.\frac{X_{1}-50}{X_{1}-X_{2}}\times \left(Mx_{2}-Mx_{1}\right)\left.\right]+Mx_{1}$$

where $X_{1}$—more than 50% of surviving cells; $X_{2}$—less than 50% of surviving cells; $Mx_{1}$—concentration of substance where more than 50% of cells survived; $Mx_{2}$—concentration of substance where less than 50% of cells survived.

### 3.5. The Determination of an Antiviral Action of the Substances In Vitro

The antiviral effect of the substances was determined according to the therapeutic scheme against the influenza A/Swine/Iowa/30 (H1N1) virus, using the MDCK cell model.

Briefly, the cell suspension was dispersed into 96-well plates at a concentration of 2.5 × 10^5^ cells/mL at 100.0 μL per well. Cell culture plates were incubated at 37 °C and 5.0% CO_2_ until a complete monolayer was formed. The virus at a dose of 100 ID/0.2 mL was used to infect the cell culture.

To determine the therapeutic activity, six concentrations of each substance were used with a dilution rate of two times, starting from the value of 1/2 CC_50_. Then, 200.0 µL of the working dilution of the virus was added to the wells of the plate. The plates were incubated for 1 h at 37 °C for virus adsorption. At the end of the incubation, the contents of the wells were removed and 200.0 µL of the final dilutions were added. An untreated virus was used as a negative control. The plates were incubated for 72 h in a CO_2_ incubator at 37 °C and 5.0% CO_2_. All studies were performed in five replicates.

The therapeutic activity of the substances against the influenza virus was detected as a result of a decrease in the infectivity titer of the residual virus in the hemagglutination reaction.

### 3.6. Statistics

The results of the conducted quantitative studies were processed using the one-way ANOVA method of one-factor analysis of variance, with further analysis using the GraphPad Prism 5 application software package.

## 4. Conclusions

Potential biologically active piperidinecarboxylic acids were synthesized through cyanohydrin synthesis, hydrochlorides of cyclopropanecarboxylic acid esters, and hydrochlorides of fluorobenzoic acid esters with an N-benzyl piperidine fragment.The synthesized compounds were then characterized using various spectroscopic techniques like ^1^H and ^13^C NMR, as well as advanced techniques like COSY, HMQC, and HMBC. This study highlights the importance of structural characterization in identifying novel compounds with valuable biological activities.

The antiviral and cytotoxic activities of the synthesized compounds were studied. The antiviral activity in vitro was determined according to the therapeutic regimen against the influenza A/Swine/Iowa/30 (H1N1) virus on the MDCK cell model. The cytotoxicity of the studied substances in vitro was assessed using the MTT test.

A comparative analysis of the data obtained indicated the presence of antiviral activity of the new synthesized piperidine derivatives against the influenza A/H1N1 virus, which demonstrated efficacy comparable to commercially available oseltamivir at similar or lower concentrations.

These results indicate support the further investigation of these compounds as potential antiviral agents against the influenza A/H1N1 virus.

## Figures and Tables

**Table 1:** The evaluation of the cytotoxic action of the investigated substances on MDCK.

Name of Substance	CC50 ± SD, mg/mL
3b	9.50 ± 0.364
5c	3.70 ± 0.189
5d	0.16 ± 0.006
8	0.07 ± 0.005
11	0.13 ± 0.002
Rimantadine	0.16 ± 0.011
Tamiflu	0.66 ± 0.091

**Table 2:** The results of the evaluation of the therapeutic activity of investigational substance **5c** on the influenza A/Swine/Iowa/30 (H1N1) virus model.

Name	Concentration, mg/mL	Titre in HAU *	Titre in HAU for PC ^1^
**5c**	1.850	3.8 ± 1.1	5.0 ± 0
	0.925	4.4 ± 0.6	
	0.463	5.0 ± 0	
	0.231	5.0 ± 0	
	0.116	5.0 ± 0	
	0.058	5.0 ± 0	
Rimantadine	0.080	4.2 ± 0.4	6.0 ± 0
	0.040	4.2 ± 0.4	
	0.020	4.2 ± 0.4	
	0.010	4.2 ± 0.4	
	0.005	4.2 ± 0.4	
	0.003	4.2 ± 0.4	

* HAU—Hemagglutinating unit. ^1^ Hemagglutinating unit titer is given in binary logarithm with the mean arithmetic standard deviation (log2 ± StD).

**Table 3:** The results of the evaluation of the therapeutic activity of the investigated substances on the influenza A/Swine/Iowa/30 (H1N1) virus model.

Name	Concentration, mg/mL	Titre in HAU *	Titre in HAU for PC ^1^
**11**	0.0650	4.0 ± 0	6.0 ± 0
	0.0325	4.0 ± 0	
	0.0163	6.0 ± 0	
	0.0081	6.0 ± 0	
	0.0041	6.0 ± 0	
	0.0020	6.0 ± 0	
**8**	0.0350	4.0 ± 0	6.0 ± 0
	0.0175	5.0 ± 0	
	0.0088	5.0 ± 0	
	0.0044	6.0 ± 0	
	0.0022	6.0 ± 0	
	0.0011	6.0 ± 0	
**3b**	4.75	4.0 ± 0	5.0 ± 0
	2.38	4.4 ± 0.6	
	1.19	5.0 ± 0	
	0.59	5.0 ± 0	
	0.30	5.0 ± 0	
	0.15	5.0 ± 0	
**5d**	0.0800	4.0 ± 0	6.0 ± 0
	0.0400	4.8 ± 0.5	
	0.0200	6.0 ± 0	
	0.0100	6.0 ± 0	
	0.0050	6.0 ± 0	
	0.0025	6.0 ± 0	
Tamiflu	0.33	2.4 ± 0.5	5.0 ± 0
	0.17	2.4 ± 0.5	
	0.08	3.0 ± 0	
	0.04	3.0 ± 0	
	0.02	4.0 ± 0	
	0.01	4.0 ± 0	

* HAU—Hemagglutinating unit. ^1^ Hemagglutinating unit titer is given in binary logarithm with the mean arithmetic standard deviation (log2 ± StD).

## Data Availability

The datasets used and/or analyzed during the present study are available from the corresponding author upon reasonable request.

## Supplementary Materials

The following supporting information can be downloaded at https://www.mdpi.com/article/10.3390/molecules30122540/s1: Table S1: Spectroscopic and physical data; Figure S1: IR spectra of compound 2a; Figure S2: IR spectra of compound 2b; Figure S3: IR spectra of compound 2c; Figure S4: IR spectra of compound 2d; Figure S5: IR spectra of compound 3a; Figure S6: IR spectra of compound 3b; Figure S7: IR spectra of compound 3c; Figure S8: IR spectra of compound 3d; Figure S9: IR spectra of compound 5c; Figure S10: IR spectra of compound 5d; Figure S11: IR spectra of compound 8; Figure S12: IR spectra of compound 11; Figure S13: ^1^H NMR spectra of compound 3a (in DMSO); Figure S14: ^13^C NMR spectra of compound 3a (in DMSO); Figure S15: COSY of compound 3a (in DMSO); Figure S16: HMQC of compound 3a (in DMSO); Figure S17: ^1^H NMR spectra of compound 3b (in DMSO); Figure S18: ^13^C NMR spectra of compound 3b (in DMSO); Figure S19: COSY of compound 3b (in DMSO); Figure S20: HMQC of compound 3b (in DMSO); Figure S21: ^1^H NMR spectra of compound 3c (in DMSO); Figure S22: ^13^C NMR spectra of compound 3c (in DMSO); Figure S23: COSY spectra of compound 3c (in DMSO); Figure S24: ^1^H NMR spectra of compound 3d (in DMSO); Figure S25: ^13^C NMR spectra of compound 3d (in DMSO); Figure S26: COSY of compound 3d (in DMSO); Figure S27: HMQC of compound 3d (in DMSO); Figure S28: ^1^H NMR spectra of compound 5c (in DMSO); Figure S29: ^13^C NMR spectra of compound 5c (in DMSO); Figure S30: COSY of compound 5c (in DMSO); Figure S31: HMQC of compound 5c (in DMSO); Figure S32: ^1^H NMR spectra of compound 5d (in DMSO); Figure S33: ^13^C NMR spectra of compound 5d (in DMSO); Figure S34: COSY of compound 5d (in DMSO); Figure S35: HMQC of compound 5d (in DMSO); Figure S36: ^1^H NMR spectra of compound 8 (in DMSO); Figure S37: ^13^C NMR spectra of compound 8 (in DMSO); Figure S38: COSY of compound 8 (in DMSO); Figure S39: ^1^H NMR spectra of compound 11 (in DMSO); Figure S40: ^13^C NMR spectra of compound 11 (in DMSO); Figure S41: COSY of compound 11 (in DMSO); Figure S42: HMQC of compound 11 (in DMSO).
