# Supplementary material for: Search for Antiviral Preparations in Series of New Derivatives of N-Substituted Piperidines
Source: Molecules. 2025 Jun 10;30(12):2540. doi: 10.3390/molecules30122540 (PMC12196486; doi:10.3390/molecules30122540)
Supplement: Supplementary file 1 [file molecules-30-02540-s001.zip › molecules-3648047-supplementary.pdf]

Supporting information

«Search for Antiviral Preparations in a Series of New Derivatives N-Substituted Piperidines»

Table of contents

Experimental 1

General Information 1

Experimental Procedures 1

Spectroscopic and physical data 3

Copies of IR Spectra of Products 7

Copies of NMR Spectra of Products 13

Experimental

General Information

Piperidin-4-ones **(1a-1d)** and benzoyl chlorides, cyclopropanecarbonyl chloride and were purchased from Sigma-Aldrich (Louis Street, MO, USA). IR spectra were recorded on a Nicolet 5700 instrument between KBr plates. ^{1}H and ^{13}C NMR spectra were recorded on a JNM-ECA Jeol 400 spectrometer (frequencies 399.78 and 100.53 MHz, respectively) using DMSO-d6 solvent. The elemental analysis data were consistent with the calculated values. Column chromatography and thin-layer chromatography were carried out on alumina (Al_2O_3) and Rf compounds were given for this type of plate. The spots were developed in iodine vapors. The IR and NMR spectra for the synthesized compounds are presented in the Supplementary File.

Experimental Procedures

Cyanohydrin of 1-methyl-4-piperidone (2a), (fig. S1). A mixture of 2 g (0.0177 mol) of 1-methyl-4-ketopiperidine (1a) and 1 mL (0.0177 mol) of acetone cyanohydrin was maintained for 15 hours at room temperature. The resulting mixture was then washed with hexane, and the precipitate was recrystallized from ethyl acetate with the addition of hexane. As a result, 1.12 g (45% of the theoretical yield) of 1-methyl-4-piperidone cyanohydrin (2a) was obtained, with a melting point of 109–111 °C and an Rf value of 0.65 (Al_2O_3, eluent — benzene : dioxane = 3:2).

Cyanohydrin of 1-propyl-4-piperidone (2b) (fig. S2). A mixture of 6 g (0.0425 mol) of 1-propyl-4-ketopiperidine (1b) and 3 mL (0.0425 mol) of acetone cyanohydrin was maintained for 15 hours at room temperature. The resulting mixture was then washed with hexane, and the precipitate was recrystallized from ethyl acetate with the addition of hexane. As a result, 4.68 g (65% of the theoretical yield) of 1-propyl-4-piperidone cyanohydrin (2b) was obtained, with a melting point of 95–97 °C, and an Rf value of 0.82 (Al_2O_3, eluent — benzene : dioxane = 3:2).

Cyanohydrin of 1-benzyl-4-piperidone (2c) (fig. S3). A mixture of 2 g (0.01 mol) of 1-benzyl-4-ketopiperidine (1c) and 1 mL (0.01 mol) of acetone cyanohydrin was maintained for 15 hours at room temperature. The resulting mixture was then washed with hexane, and the precipitate was recrystallized from ethyl acetate with the addition of hexane. As a result, 1.85 g (81% of the theoretical yield) of 1-benzyl-4-piperidone cyanohydrin (2c) was obtained, with a melting point of 87–89 °C and an Rf value of 0.83 (Al_2O_3, eluent — benzene : dioxane = 3:2).

Cyanohydrin of 1-(2-phenylethyl)-4-piperidone (2d) (fig. S4). A mixture of 2 g (0.01 mol) of 1-(2-phenylethyl)-4-ketopiperidine (1d) and 1 mL (0.01 mol) of acetone cyanohydrin was maintained for 22 hours at room temperature. The resulting mixture was then washed with hexane, and the precipitate was recrystallized from ethyl acetate with the addition of hexane. As a result, 1.9 g (84% of the theoretical yield) of

1-(2-phenylethyl)-4-piperidone cyanohydrin (2d) was obtained, with a melting point of 82–84 °C and an Rf value of 0.84 (Al_2O_3, eluent — benzene : dioxane = 3:2).

Hydrochloride of 1-methyl-4-carboxy-4-hydroxypiperidine (3a) (fig. S5, fig. S13 – S16). A solution of 5.1 g (0.0364 mol) of 1-methyl-4-piperidone cyanohydrin (2a) in 50 mL (1.3698 mol) of concentrated hydrochloric acid and 25 mL (0.4166 mol) of acetic acid was maintained at room temperature for seven days. The reaction mixture was then basified to pH 10 with a concentrated sodium hydroxide solution and extracted with benzene. The aqueous layer was acidified to pH 1, evaporated to dryness at 55–60 °C, and the residue was treated sequentially with acetone and then isopropanol. From the resulting solution, 5.55 g (79% of the theoretical yield) of 1-methyl-4-carboxy-4-hydroxypiperidine hydrochloride (3a) was isolated as a white precipitate, which was recrystallized from isopropanol. Melting point: 170–172 °C.

Hydrochloride of 1-propyl-4-carboxy-4-hydroxypiperidine (3b), (fig. S6, fig. S17 – S20). A solution of 4.68 g (0.0278 mol) of 1-propyl-4-piperidone cyanohydrin (2b) in 47 mL (1.2876 mol) of concentrated hydrochloric acid and 23.5 mL (0.3916 mol) of acetic acid was maintained at room temperature for seven days. The reaction mixture was then basified to pH 10 with a concentrated sodium hydroxide solution and extracted with benzene. The aqueous layer was acidified to pH 1, evaporated to dryness at 55–60 °C, and the residue was treated sequentially with acetone and then isopropanol. From the resulting solution, 3.59 g (70% of the theoretical yield) of 1-propyl-4-carboxy-4-hydroxypiperidine hydrochloride (3b) was isolated as a white precipitate, hich was recrystallized from isopropanol. Melting point: 173–175 °C.

Hydrochloride of 1-benzyl-4-carboxy-4-hydroxypiperidine (3c), (fig. S7, fig. S21 – S23). A solution of 3.62 g (0.0167 mol) of 1-benzyl-4-piperidone cyanohydrin (2c) in 36 mL (0.9863 mol) of concentrated hydrochloric acid and 18 mL (0.30 mol) of acetic acid was maintained at room temperature for seven days. The reaction mixture was then basified to pH 10 with a concentrated sodium hydroxide solution and extracted with benzene. The aqueous layer was acidified to pH 1, evaporated to dryness at 55–60 °C, and the residue was treated sequentially with acetone and then isopropanol. From the resulting solution, 2.88 g (63% of the theoretical yield) of 1-benzyl-4-carboxy-4-hydroxypiperidine hydrochloride (3c) was obtained as an oil.

Hydrochloride of 1-(2-phenylethyl)-4-carboxy-4-hydroxypiperidine (3d), (fig. S8, fig. S24 – S27). A solution of 3.35 g (0.0145 mol) of 1-(2-phenylethyl)-4-piperidone cyanohydrin (2d) in 33.5 mL (0.9178 mol) of concentrated hydrochloric acid and 17 mL (0.2833 mol) of acetic acid was maintained at room temperature for seven days. The reaction mixture was then basified to pH 10 with a concentrated sodium hydroxide solution and extracted with benzene. The aqueous layer was acidified to pH 1, evaporated to dryness at 55–60 °C, and the residue was treated sequentially with acetone and then isopropanol. From the resulting solution, 3.67 g (88% of the theoretical yield) of 1-(2-phenylethyl)-4-carboxy-4-hydroxypiperidine hydrochloride (3d) was isolated as a white precipitate, which was recrystallized from isopropanol. Melting point: 160–162 °C.

Hydrochloride of 1-benzyl-4-cyclopropanoyloxy-piperidine-4-carboxylic acid (5c), (fig. S9, fig. S28 – S31). To a stirred mixture of 2 g (0.0074 mol) of 1-benzyl-4-carboxy-4-hydroxypiperidine hydrochloride (3 c), 14 mL (0.1339 mmol) of cyclopropanecarbonyl chloride (4) was added dropwise. An exothermic reaction occurred, accompanied by the formation of a white precipitate. The reaction mixture was maintained for 12 hours at room temperature. The resulting precipitate was washed with diethyl ether, and the residue was recrystallized from acetone. As a result, 1.49 g (59% of the theoretical yield) of 1-benzyl-4-cyclopropanoyloxy-piperidine-4-carboxylic acid hydrochloride (5c) was obtained, with a melting point of 208–210 °C and an Rf value of 0.80 (Al_2O_3, eluent — benzene : dioxane = 3:2).

Hydrochloride of 1-(2-phenylethyl)-4-cyclopropanoyloxy-piperidine-4-carboxylic acid (5d), (fig. S10, fig. S32 – S35). To a stirred mixture of 1.74 g (0.0061 mol) of 1-(2-phenylethyl)-4-carboxy-4-hydroxypiperidine hydrochloride (3d), 7.0 mL (0.0772 mol) of cyclopropanecarbonyl chloride (4) was added dropwise. An exothermic reaction occurred, accompanied by the formation of a white precipitate. The reaction mixture was maintained for 12 hours at room temperature. The resulting precipitate was washed with diethyl ether, and the residue was recrystallized from acetone. As a result, 1.3 g (60% of the theoretical yield) of 1-(2-phenylethyl)-4-cyclopropanoyloxy-piperidine-4-carboxylic acid hydrochloride (5d) was obtained, with a melting point of 167–169 °C and an Rf value of 0.81 (Al_2O_3, eluent — benzene : dioxane = 3:2).

Hydrochloride of 1-benzyl-4-trifluoromethylbenzoyloxy-piperidine (8), (fig. S11, fig. S36 – S38). To a stirred solution of 2.53 g (0.0132 mol) of 1-benzyl-4-hydroxypiperidine (6) in chloroform, a solution of 4.14 g (0.0198 mol) of trifluoromethylbenzoyl chloride (7) in chloroform was added dropwise. An exothermic

reaction and a color change of the reaction mixture were observed. The reaction mixture was maintained for 12 hours at room temperature. The resulting white precipitate was filtered off, washed with diethyl ether, and the residue was recrystallized from isopropanol. As a result, 2.87 g (54% of the theoretical yield) of 1-benzyl-4-trifluoromethylbenzoyloxy-piperidine hydrochloride (8) was obtained, with a melting point of 200–202 °C and an Rf value of 0.90 (Al_2O_3, eluent — benzene : dioxane = 3:2).

Hydrochloride of 1-benzyl-4-(2,6-difluoro-benzoyloxymino) piperidine (11), fig. S12, fig. S39 – S42). 2 g (0.0098 mol) of 1-benzyl-4-piperidone oxime (9) were dissolved in a small amount of absolute dioxane. To this solution, with stirring, a solution of 2.46 mL (0.0196 mol) of 2,6-difluorobenzoyl chloride (10) in absolute dioxane was added dropwise. An exothermic reaction was observed, and during stirring, a white precipitate formed. The reaction mixture was maintained at room temperature for 24 hours. The progress of the reaction was monitored by TLC. The reaction mixture was washed with diethyl ether, and the resulting precipitate was filtered off and recrystallized from isopropanol. As a result, 3.52 g (94% of the theoretical yield) of 1-benzyl-2,6-difluorobenzoyloxy-ketopiperidine oxime hydrochloride (11) was obtained, with a melting point of 162–164 °C and an Rf value of 0.90 (Al_2O_3, eluent — benzene : dioxane = 4:1).

Table 1. Spectroscopic and physical data

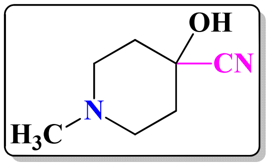<br>**2a**<br>**Fig.S1**	**Cyanohydrin of 1-methyl-4-piperidone (2a).**<br>White powder, yield 45%, melting point 109-111°C.<br>IR spectrum (KBr), ν, см$^{-1}$: 2230.5 (C≡N).<br><br>Found, %: Carbon (C) 60,12; Hydrogen (H) 9,01; Nitrogen (N) 20,23; $C_7H_{12}N_2O$.<br>Calculated, %: Carbon (C) 59,96; Hydrogen (H) 8,56; Nitrogen (N) 19,98.
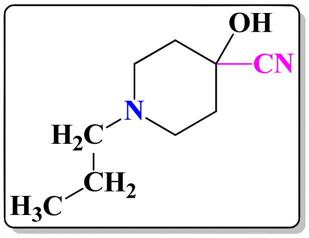<br>**2b**<br>**Fig.S2**	**Cyanohydrin of 1-propyl-4-piperidone (2b).**<br>White powder, yield 65%, melting point 95-97°C.<br>IR spectrum (KBr), ν, см$^{-1}$: 2802.2 (C≡N).<br><br>Found, %: Carbon (C) 65,37; Hydrogen (H) 9,78; Nitrogen (N) 17,09; $C_9H_{16}N_2O$.<br>Calculated, %: Carbon (C) 64,23; Hydrogen (H) 9,51; Nitrogen (N) 16,65.
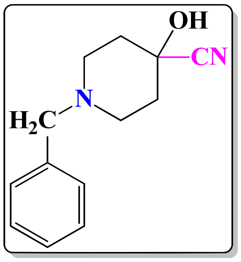<br>**2c**<br>**Fig.S3**	**Cyanohydrin of 1-benzyl-4-piperidone (2c).**<br>White powder, yield 81%, melting point 87-89°C.<br>IR spectrum (KBr), ν, см$^{-1}$: 2361.3 (C≡N).<br><br>Found, %: Carbon (C) 73,82; Hydrogen (H) 8,13; Nitrogen (N) 13,21; $C_{13}H_{16}N_2O$.<br>Calculated, %: Carbon (C) 72,17; Hydrogen (H) 7,40; Nitrogen (N) 12,95.
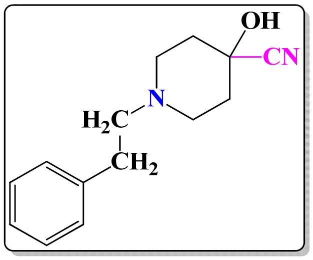<br>**2d**<br>**Fig.S4**	**Cyanohydrin of 1-(2-phenylethyl)-4-piperidone (2d).**<br>White powder, yield 84%, melting point 82-84°C.<br>IR spectrum (KBr), ν, см$^{-1}$: 2304.2 (C≡N).<br><br>Found, %: Carbon (C) 73,47; Hydrogen (H) 8,33; Nitrogen (N) 13,06; $C_{14}H_{18}N_2O$.<br>Calculated, %: Carbon (C) 72,99; Hydrogen (H) 7,82; Nitrogen (N) 12,16.

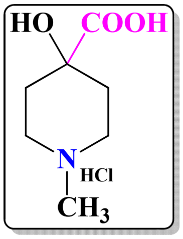<br>3a<br><br>**Fig.S5, Fig.S13 – Fig.S16**	**Hydrochloride of 1-methyl-4-carboxy-4-hydroxypiperidine (3a).**<br>White powder, yield 79%, melting point 170-172°C.<br>IR spectrum (KBr), ν, cм$^{-1}$: 1723.6 (C=O).<br><br>^{1}H NMR spectrum (DMSO-d$_6$), δ, ppm (J, Hz):<br>1.73 d (2H, H^{3_ax}, H^{5_ax}, ^{3}J = 13.6 Hz), 2.12 t (2H, H^{3_eq}, H^{5_eq}, ^{3}J = 12.6 Hz), 2.64 s (3H, H^{7,7,7}), 3.00 t (2H, H^{2_ax}, H^{6_ax}, ^{3}J = 12.6 Hz), 3.20 d (2H, H^{2_eq}, H^{6_eq}, ^{3}J = 10.4 Hz), 5.70 br s (1H, H^{8}), 7.30-7.56 (1H, H^{12}), 10.84 br s (1H, H^{11}).<br>^{13}C NMR spectrum (DMSO-d$_6$), δC, ppm:<br>31.41 (C^{3,5}), 42.87 (C^{7}), 49.27 (C^{2,6}), 68.43 (C^{4}), 176.31 (C^{9}).<br>COSY NMR spectrum (DMSO-d$_6$), δC, ppm: H^{3ax,5ax}→H^{3eq,5eq}, H^{2ax,6ax}→H^{2eq,6eq}, H^{17}→H^{14}.<br>HMQC NMR spectrum: H^{3ax,5ax}→C^{3,5}, H^{3eq,5eq}→C^{3,5}, H^{7}→C^{7}, H^{2ax,6ax}→C^{2,6}, H^{2eq,6eq}→C^{2,6}.<br><br>Found, %: Carbon (C) 44,16; Hydrogen (H) 8,25; Nitrogen (N) 7,63; $C_7H_{14}ClNO_3$.<br>Calculated, %: Carbon (C) 43,06; Hydrogen (H) 7,17; Nitrogen (N) 7,17.
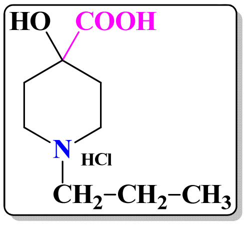<br>3b<br><br>**Fig.S6, Fig.S17 – Fig.S20**<br>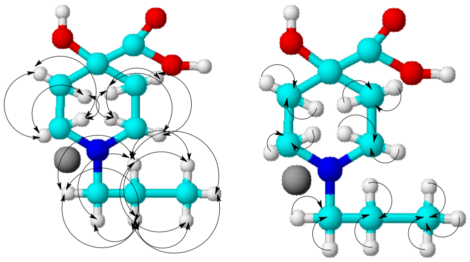<br>**(a)** **(b)**	**Hydrochloride of 1-propyl-4-carboxy-4-hydroxypiperidine (3b).**<br>White powder, yield 70%, melting point 173-175°C.<br>IR spectrum (KBr), ν, cм$^{-1}$: 1741.8 (C=O).<br><br>^{1}H NMR spectrum (DMSO-d$_6$), δ, ppm (J, Hz): 0.82 t (3H, H^{9,9,9}, ^{3}J 7.4), 1.66-1.76 m (4H, H^{8,8,3ax,5ax}), 2.19 t (2H, H^{3eq,5eq}, ^{3}J 13.6), 2.64 s (3H, H^{7,7,7}), 2.88-2.95 m (4H, H^{7,7,2ax,6ax}), 3.28 d (2H, H^{2eq,6eq}, ^{3}J 10.8), 5.66 br s (1H, H^{10}), 7.28-7.53 m (1H, H^{14}), 10.78 br s (1H, H^{13}),. ^{1}<br>^{3}C NMR spectrum (DMSO-d$_6$), δC, ppm: 11.52 (C^{9}), 17.14 (C^{8}), 31.27 (C^{3,5}), 47.60 (C^{2,6}), 57.80 (C^{7}), 68.89 (C^{4}), 176.24 (C^{11}),<br>COSY NMR spectrum (DMSO-d$_6$), δC, ppm: H^{9}→H^{8}, H^{8}→H^{7}, H^{3ax,5ax}→H^{3eq,5eq}, H^{3ax,5ax}→H^{2ax,6ax}, H^{2ax,6ax}→H^{2eq,6eq}.<br>HMQC NMR spectrum: H^{9}→C^{9}, H^{8}→C^{8}, H^{3ax,5ax}→C^{3,5}, H^{3eq,5eq}→C^{3,5}, H^{7}→C^{7}, H^{2ax,6ax}→C^{2,6}, H^{2eq,6eq}→C^{2,6}.<br><br>Found, %: Carbon (C) 49,38; Hydrogen (H) 8,96; Nitrogen (N) 7,32; $C_9H_{18}ClNO_3$.<br>Calculated, %: Carbon (C) 48,41; Hydrogen (H) 8,06; Nitrogen (N) 6,27.
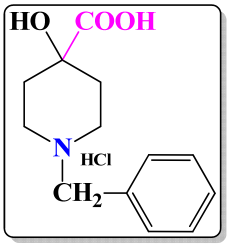<br>3c<br><br>**Fig.S7, Fig.S21 – Fig.S23**	**Hydrochloride of 1-benzyl-4-carboxy-4-hydroxypiperidine (3c).**<br>White powder, yield 63%, oil.<br>IR spectrum (KBr), ν, cм$^{-1}$: 1741.9 (C=O).<br><br>^{1}H NMR spectrum (DMSO-d$_6$), δ, ppm (J, Hz): 1.74 d (2H, H^{3ax,5ax}, ^{3}J 13.5), 2.20 t (2H, H^{3eq,5eq}, ^{3}J 13.5), 3.00 к (2H, H^{2ax,6ax}, ^{3}J 10.8), 3.14 d (2H, H^{2eq,6eq}, ^{3}J 10.8), 4.23 d (2H, H^{7,7}, ^{4}J 4.0), 7.36 s (3H, H^{9,11,13}), 7.59 s (2H, H^{10,12}), 5.39 br s (1H, H^{14}), 10.97 br s (1H, H^{17}).<br>^{13}C NMR spectrum (DMSO-d$_6$), δC, ppm: 31.15 (C^{3,5}), 47.17 (C^{2,6}), 68.85 (C^{4}), 59.19 (C^{7}), 129.22 (C^{9,11,13}), 130.05 (C^{8}), 132.13 (C^{10,12}), 176.23 (C^{15}).<br>COSY NMR spectrum (DMSO-d$_6$), δC, ppm: H^{3ax,5ax}→H^{3eq,5eq}, H^{2ax,6ax}→H^{2eq,6eq}, H^{9,11,13}→H^{10,12}.<br><br>Found, %: Carbon (C) 58,61; Hydrogen (H) 7,14; Nitrogen (N) 6,39; $C_{13}H_{18}ClNO_3$.

	Calculated, %: Carbon (C) 57,74; Hydrogen (H) 6,63; Nitrogen (N) 5,16.
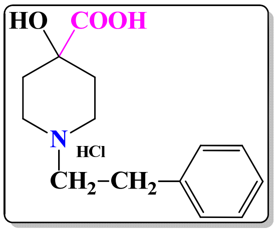<br>3d<br>**Fig.S8, Fig.S24 – Fig.S27**	**Hydrochloride of 1-(2-phenylethyl)-4-carboxy-4-hydroxypiperidine (3d).**<br>White powder, yield 88%, melting point 160-162°C.<br>IR spectrum (KBr), ν, см$^{-1}$: 1734.9 (C=O).<br><br>^{1}H NMR spectrum (DMSO-d$_6$), δ, ppm (J, Hz): 1.79 d (2H, H^{3ax,5ax}, ^{3}J 13.6), 2.24 t (2H, H^{3eq,5eq}, ^{3}J 12.6), 3.06 s (4H, H^{8,8,2ax,6ax}), 3.40 d (2H, H^{2eq,6eq}, ^{3}J 10.0), 3.20 t (2H, H^{7,7}, ^{3}J 7.2), 7.18-7.28 m (5H, H$^{10-14}$), 5.72 br s (1H, H^{15}), 7.28-7.57 m (1H, H^{19}), 11.10 br s (1H, H^{18}).<br>^{13}C NMR spectrum (DMSO-d$_6$), δC, ppm: 29.84 (C^{8}), 31.38 (C^{3,5}), 47.69 (C^{2,6}), 57.03 (C^{7}), 68.92 (C^{4}), 127.26 (C^{12}), 129.14 (C$^{10-14}$), 137.14 (C^{9}), 176.28 (C^{16}),<br>COSY NMR spectrum (DMSO-d$_6$), δC, ppm: H^{3ax,5ax}→H^{3eq,5eq}, H^{3eq,5eq}→C^{3,5}, H^{2ax,6ax}→H^{2eq,6eq}, H^{8}→H^{7}, H^{3ax,5ax}→H^{2ax,6ax}. HMQC NMR spectrum: H^{3ax,5ax}→C^{3,5}, H^{3eq,5eq}→C^{3,5}, H^{2ax,6ax}→C^{2,6}, H^{2eq,6eq}→C^{2,6}, H^{8}→C^{8}, H^{7}→C^{7}.<br><br>Found, %: Carbon (C) 59,67; Hydrogen (H) 7,92; Nitrogen (N) 5,24; $C_{14}H_{20}ClNO_3$.<br>Calculated, %: Carbon (C) 58,84; Hydrogen (H) 7,00; Nitrogen (N) 4,90.
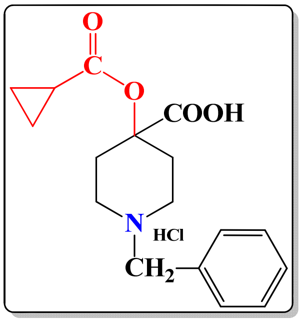<br>5c<br>**Fig.S9, Fig.S28 – Fig.S31**<br>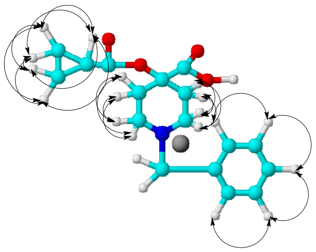<br>**(a)**<br>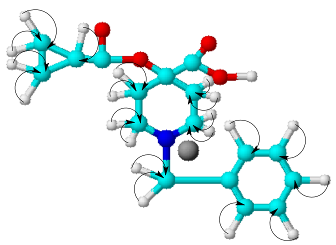<br>**(b)**	**Hydrochloride of 1-benzyl-4-cyclopropanoyloxy-piperidine-4-carboxylic acid (5c).**<br>White powder, yield 59%, melting point 208-210°C.<br>IR spectrum (KBr), ν, см$^{-1}$: 1730.2 (C=O) ester, 1421.8 (C=O) carboxylic.<br><br>^{1}H NMR spectrum (DMSO-d$_6$), δ, ppm (J, Hz): 0.81-0.84 m (4H, H^{20ax,21ax,20eq,21eq}), 1.66 s (1H, H^{19}), 2.17 d (2H, H^{3ax,5ax}, ^{3}J 13.6), 239 t (2H, H^{3eq,5eq}, ^{3}J 11.6), 3.01 s (2H, H^{2ax,6ax}), 3.19 d (2H, H^{2eq,6eq}), 4.30 s (1H, H^{7,7}), 7.38 s (3H, H^{9,11,13}), 7.65 s (2H, H^{10,12}), 11.45 br s (1H, H^{22}).<br>^{13}C NMR spectrum (DMSO-d$_6$), δC, ppm: 9.09 (C^{20,21}), 13.32 (C^{19}), 29.02 (C^{3,5}), 47.10 (C^{2,6}), 58.96 (C^{7}), 75.36 (C^{4}), 129.19 (C^{9,13}), 129.92 (C^{11}), 130.26 (C^{8}), 132.06 (C^{10,12}), 172.04 (C^{17}), 173.30 (C^{15}).<br>COSY NMR spectrum (DMSO-d$_6$), δC, ppm: H^{20,21}→H^{19}, H^{9,11,13}→H^{10,12}, H^{3ax,5ax}→H^{3eq,5eq}.<br>HMQC NMR spectrum: H^{20,21}→C^{20,21}, H^{19}→C^{19}, H^{3ax,5ax}→C^{3,5}, H^{3eq,5eq}→C^{3,5}, H^{7}→C^{7}, H^{9,11,13}→C^{9,11,13}.<br><br>Found, %: Carbon (C) 61,33; Hydrogen (H) 7,52; Nitrogen (N) 5,04; $C_{17}H_{22}ClNO_4$.<br>Calculated, %: Carbon (C) 60,15; Hydrogen (H) 6,48; Nitrogen (N) 4,12.

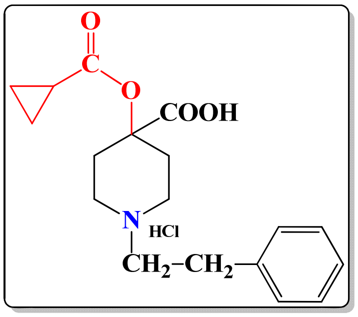

5d

Fig.S10, Fig.S32 – Fig.S35

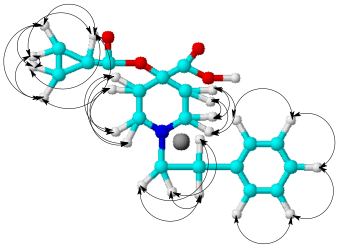

(a)

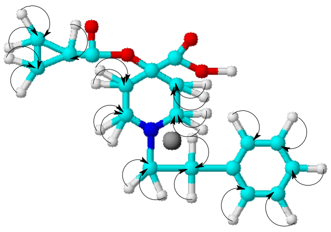

(b)

Hydrochloride of 1-(2-phenylethyl)-4-cyclopropanoyloxy-piperidine-4-carboxylic acid (5d).

White powder, yield 60%, melting point 167-169°C.

IR spectrum (KBr), ν, см$^{-1}$: 1717.9 (C=O) ester, 1468.3 (C=O) carboxylic.

^{1}H NMR spectrum (DMSO-d$_6$), δ, ppm (J, Hz): 0.79-0.93 m (4H, H^{21ax,22ax,21eq,22eq}), 1.73 s (1H, H^{20}), 2.19 d (2H, H^{3ax,5ax}, ^{3}J 13.8), 2.39 t (2H, H^{3eq,5eq}, ^{3}J 12.6), 3.06 br s (2H, H^{8,8,2ax,6ax}), 3.28 s (1H, H^{7,7}), 3.52 s (2H, H^{2eq,6eq}), 4.84-4.87 m (1H, H^{24}), 7.21-7.29 m (5H, H$^{10-14}$), 11.45 br s (1H, H^{23}).

^{13}C NMR spectrum (DMSO-d$_6$), δC, ppm: 9.14 (C^{21,22}), 13.17 (C^{20}), 29.13 (C^{3,5}), 47.27 (C^{2,6}), 56.79 (C^{8}), 69.32 (C^{7}), 75.26 (C^{4}), 127.31 (C^{12}), 129.19 (C^{9,10,11,13}), 137.65 (C^{9}), 169.93 (C^{18}), 172.01 и 173.47 (C^{16}).

COSY NMR spectrum (DMSO-d$_6$), δC, ppm: H^{21,22}→H^{20}, H^{3ax,5ax}→H^{3eq,5eq}, H^{2ax,6ax}→H^{2eq,6eq}.

HMQC NMR spectrum: H^{21,22}→C^{21,22}, H^{19}→C^{19}, H^{3ax,5ax}→C^{3,5}, H^{3eq,5eq}→C^{3,5}, H^{8}→C^{8}.

Found, %: Carbon (C) 62,36; Hydrogen (H) 7,53; Nitrogen (N) 4,27; $C_{18}H_{24}ClNO_4$.

Calculated, %: Carbon (C) 61,16; Hydrogen (H) 6,79; Nitrogen (N) 3,96.

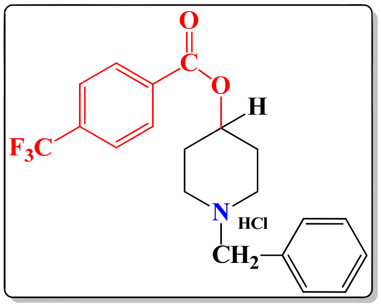

8

Fig.S11, Fig.S36 – Fig.S38

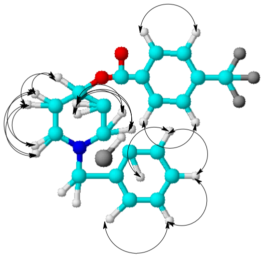

(a)

Hydrochloride of 1-benzyl-4-trifluoromethylbenzoyloxy-piperidine (8).

White powder, yield 54%, melting point 200-202°C.

IR spectrum (KBr), ν, см$^{-1}$: 1723.1 (C=O), 1543.2, 1474.5 (arom.).

^{1}H NMR spectrum (DMSO-d$_6$), δ, ppm (J, Hz): 2.03-2.31 m (4H, H^{3ax,5ax,3eq,5eq}), 3.09-3.36 m (4H, H^{2ax,6ax,2eq,6eq}), 5.05-5.24 m (1H, H^{4}), 4.7-4.36 m (2H, H^{7,7}), 7.40-7.41 m (3H, H^{10,11,12}), 7.61-7.68 m (2H, H^{13,19}), 7.85-7.88 m (2H, H^{19,21}), 8.11-8.25 m (2H, H^{18,22}). ^{13}C NMR spectrum (DMSO-d$_6$), δC, ppm: 26.86 and 27.79 (C^{3,5}), 47.18 and 49.57 (C^{2,6}), 66.45 and 68.85 (C^{4}), 58.61 and 59.19 (C^{7}), 122.89 and 125.60 (C-23), 126.32 (C^{19,21}), 129.25 (C^{9,13}), 129.93 (C^{11}), 130.43 (C^{8}), 130.68 (C^{18,22}), 131.99 (C^{10,12}), 133.61 and 134.03 (C^{20}), 133.30 (C^{17}), 164.44 (C^{15}).

Found, %: Carbon (C) 61,54; Hydrogen (H) 6,02; Nitrogen (N) 4,36; $C_{20}H_{21}ClF_3NO_2$.

Calculated, %: Carbon (C) 60,02; Hydrogen (H) 5,25; Nitrogen (N) 3,50.

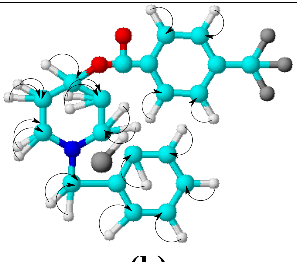<br>**(b)**	
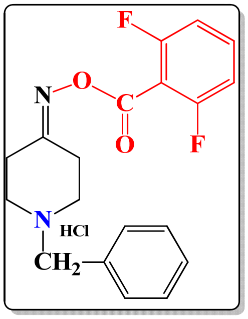<br>**11**<br><br>**Fig.S12, Fig.S39 – Fig.S42**<br><br>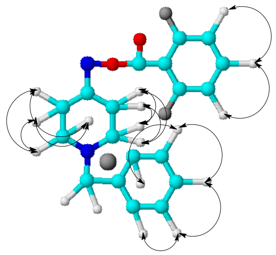<br>**(a)**<br><br>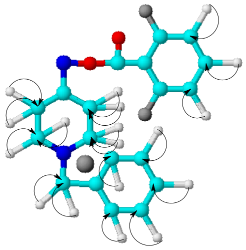<br>**(b)**	**Hydrochloride of 1-benzyl-4-ketopiperidine 2,6-difluorobenzoate oxime (11).**<br>White powder, yield 94%, melting point 162-164°C.<br>IR spectrum (KBr), ν, см$^{-1}$: 1754.0 (C=O), 1619.7 (C=N), 1468.6, 1413.6 (arom.).<br><br>^{1}H NMR spectrum (DMSO-d$_6$), δ, ppm (J, Hz): 2.75-3.34 m (8H, $H^{2ax,6ax,2eq,6eq,3ax,5ax,3eq,5eq}$), 4.34 s (2H, $H^{7,7}$), 7.24-7.28 m (2H, $H^{21,22}$), 7.39-7.41 m (3H, $H^{10,12,20}$), 7.61-7.71 m (2H, $H^{9,13,21}$), 11.89 br s (1H, H^{26}).<br>^{13}C NMR spectrum (DMSO-d$_6$), δC, ppm: 23.95 and 27.71 ($C^{3,5}$), 49.07 and 50.11 ($C^{2,6}$), 29.13 ($C^{3,5}$), 47.27 ($C^{2,6}$), 58.62 (C^{7}), 69.32 (C^{7}), 108.94, 109.13 and 109.32 (C^{18}), 113.13 and 113.38 ($C^{20,22}$), 129.31 ($C^{10,12}$), 130.01 (C^{11}), 130.25 (C^{8}), 131.88 ($C^{9,13}$), 135.24, 135.35 and 135.45 (C^{21}), 158.94 and 161.48 ($C^{19,23}$), 158.57 (C^{16}), 163.86 (C^{4}).<br>COSY NMR spectrum (DMSO-d$_6$), δC, ppm: $H^{11}\rightarrow H^{10,12}$, $H^{20}\rightarrow H^{21}$.<br>HMQC NMR spectrum: $H^{3,5}\rightarrow C^{3,5}$, $H^{2,6}\rightarrow C^{2,6}$, $H^{7}\rightarrow C^{7}$, $H^{11}\rightarrow C^{11}$, $H^{22}\rightarrow C^{22}$, $H^{10,12}\rightarrow C^{10,12}$, $H^{9,13}\rightarrow C^{9,13}$.<br><br>Found, %: Carbon (C) 60,31; Hydrogen (H) 5,22; Nitrogen (N) 8,61 $C_{19}H_{19}N_2O_2F_2Cl$.<br>Calculated, %: Carbon (C) 59,98; Hydrogen (H) 4,99; Nitrogen (N) 7,36

Copies of IR Spectra of Products

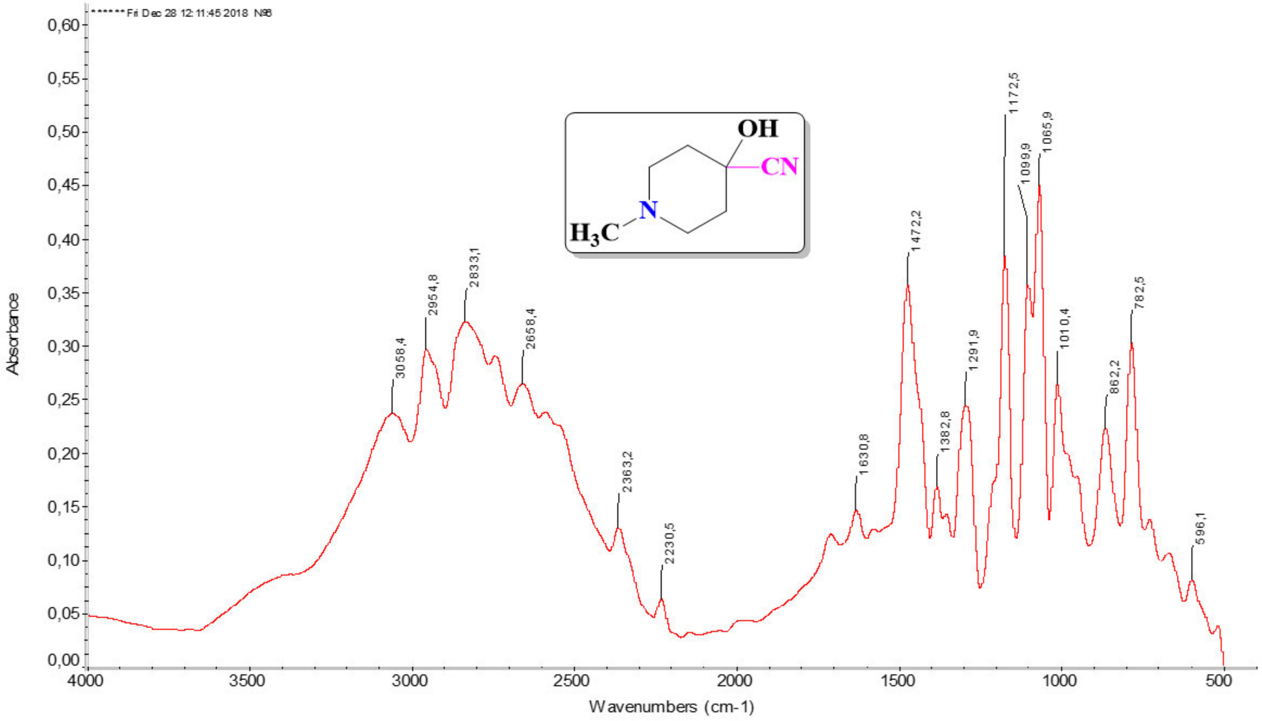

Fig. S1. IR spectra of compound **2a**

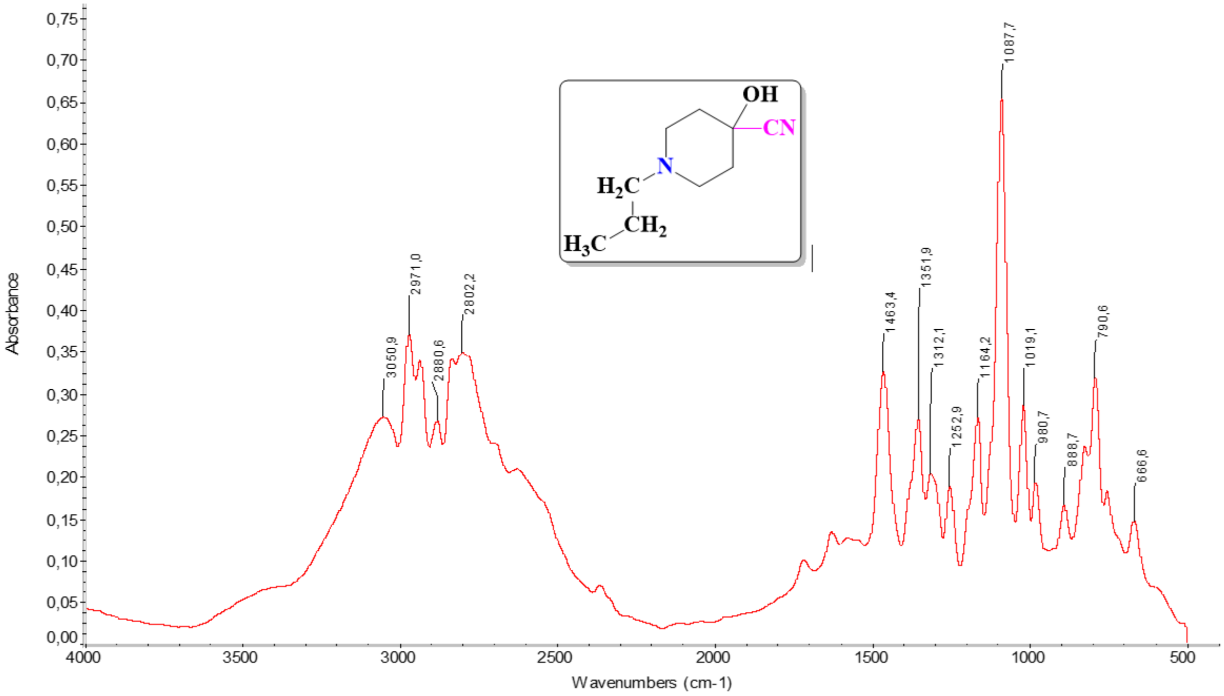

Fig. S2. IR spectra of compound **2b**

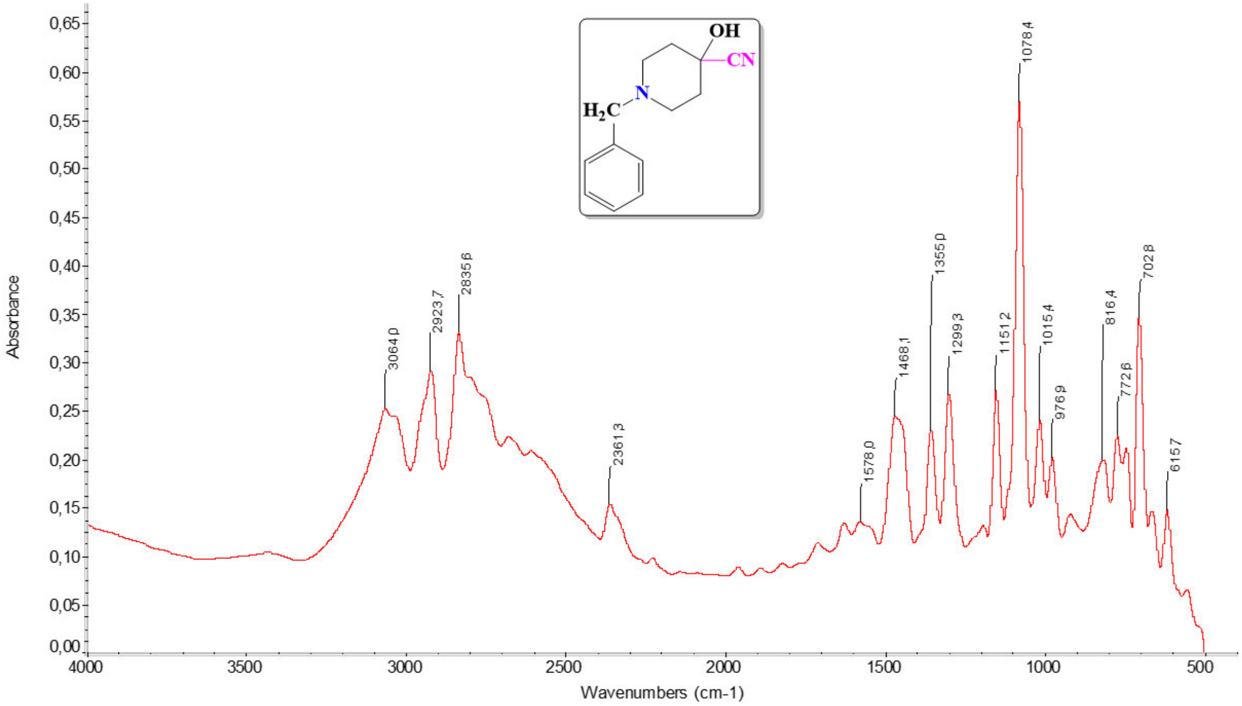

Fig. S3. IR spectra of compound **2c**

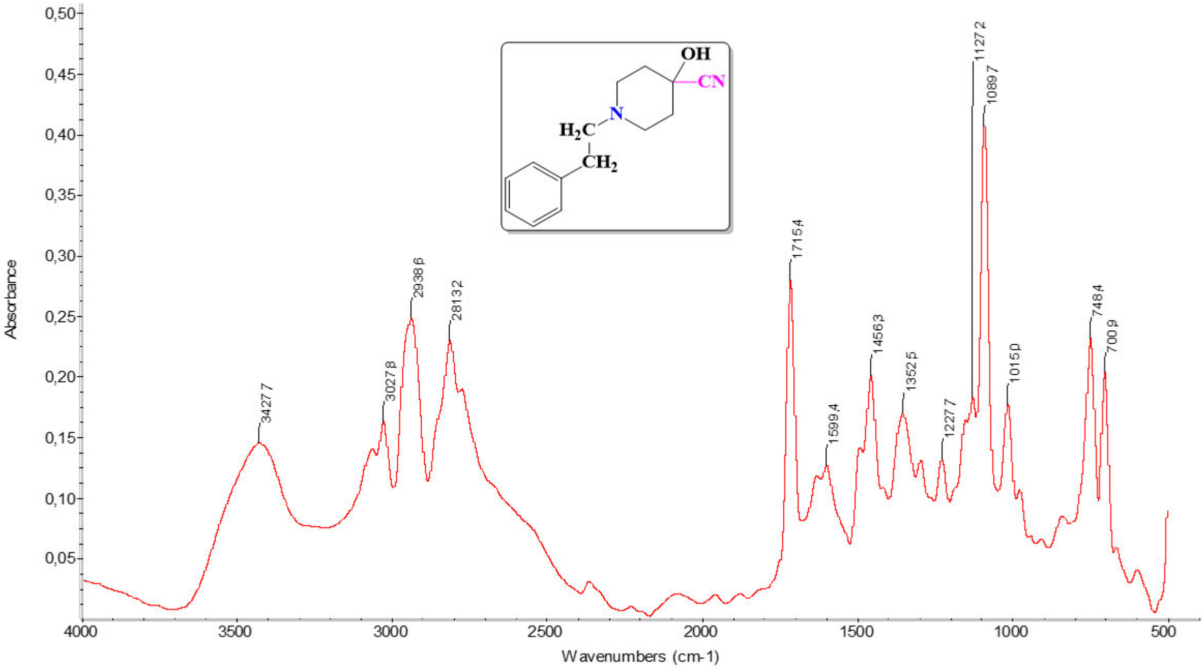

Fig. S4. IR spectra of compound **2d**

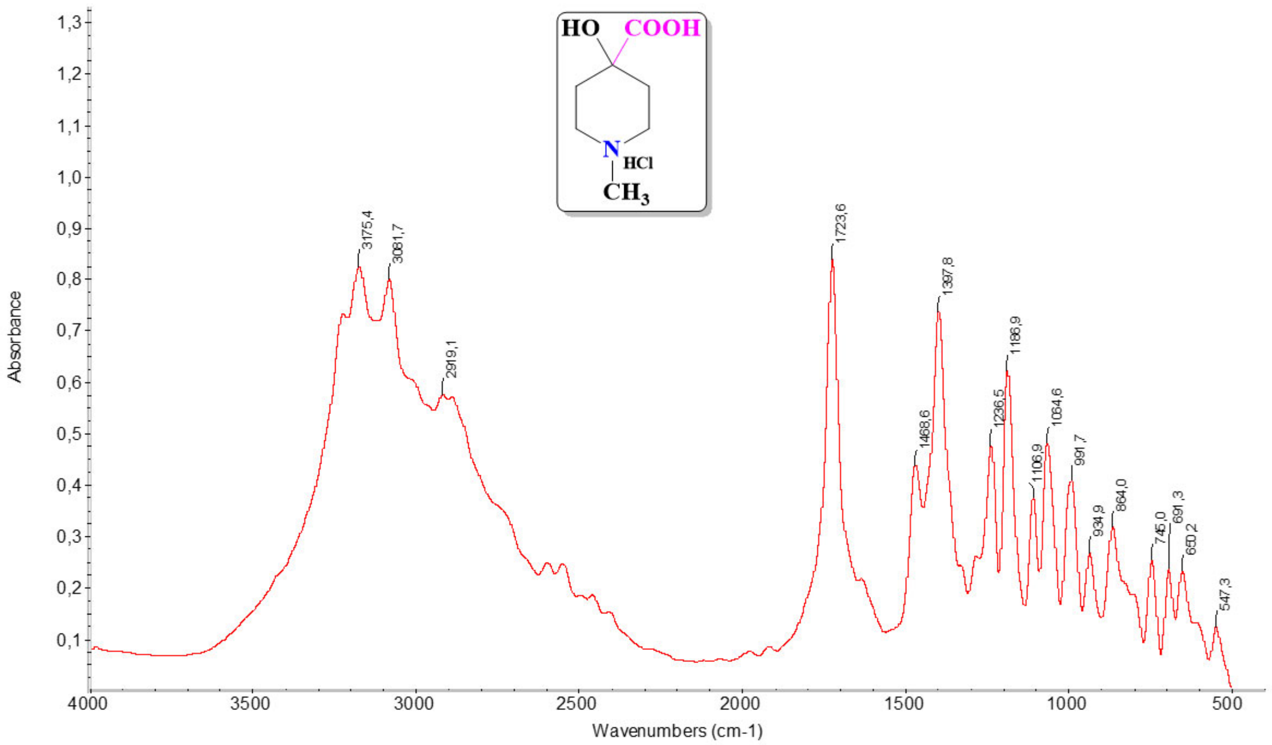

Fig. S5. IR spectra of compound **3a**

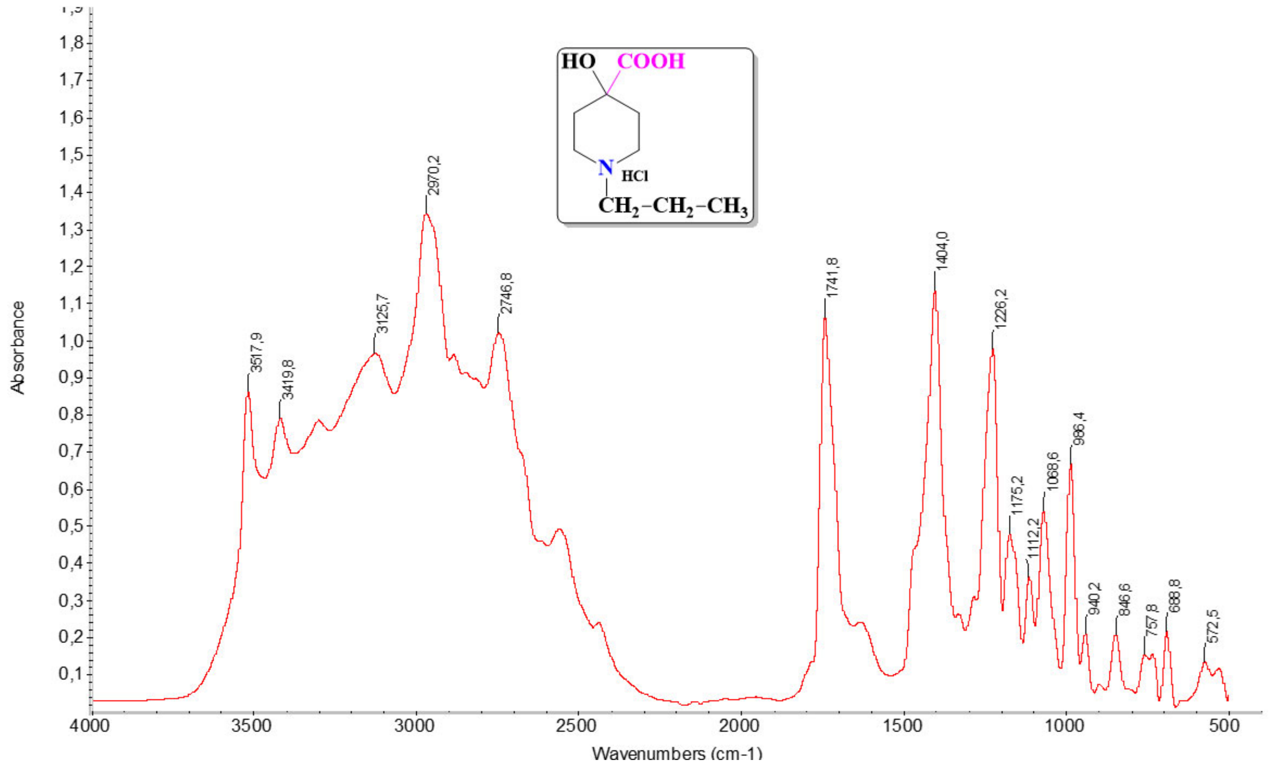

Fig. S6. IR spectra of compound **3b**

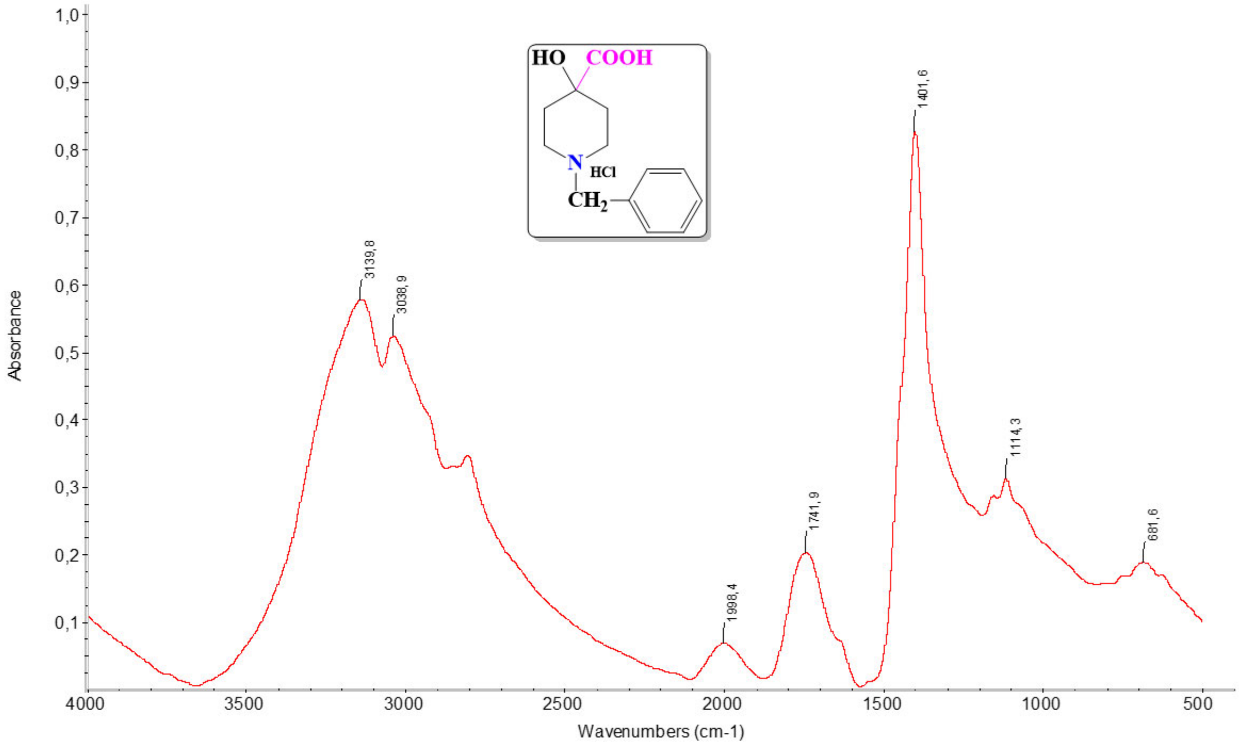

Fig. S7. IR spectra of compound **3c**

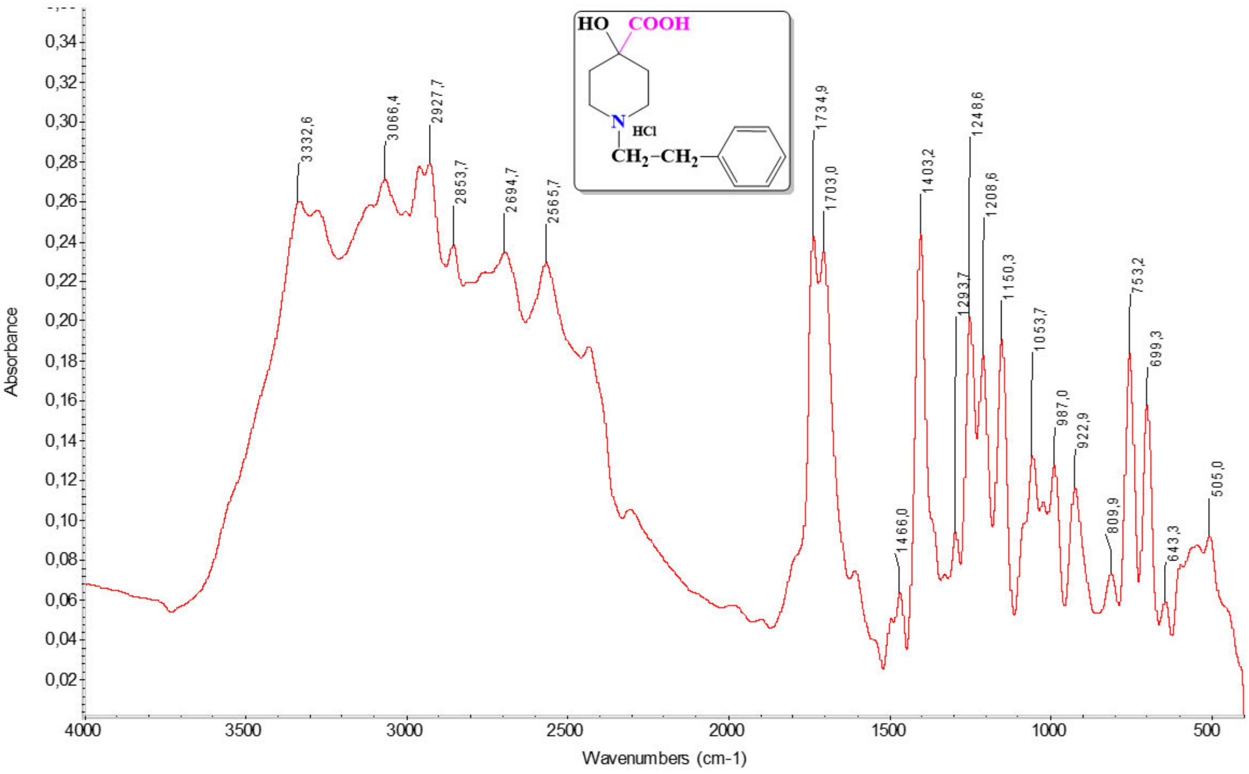

Fig. S8. IR spectra of compound **3d**

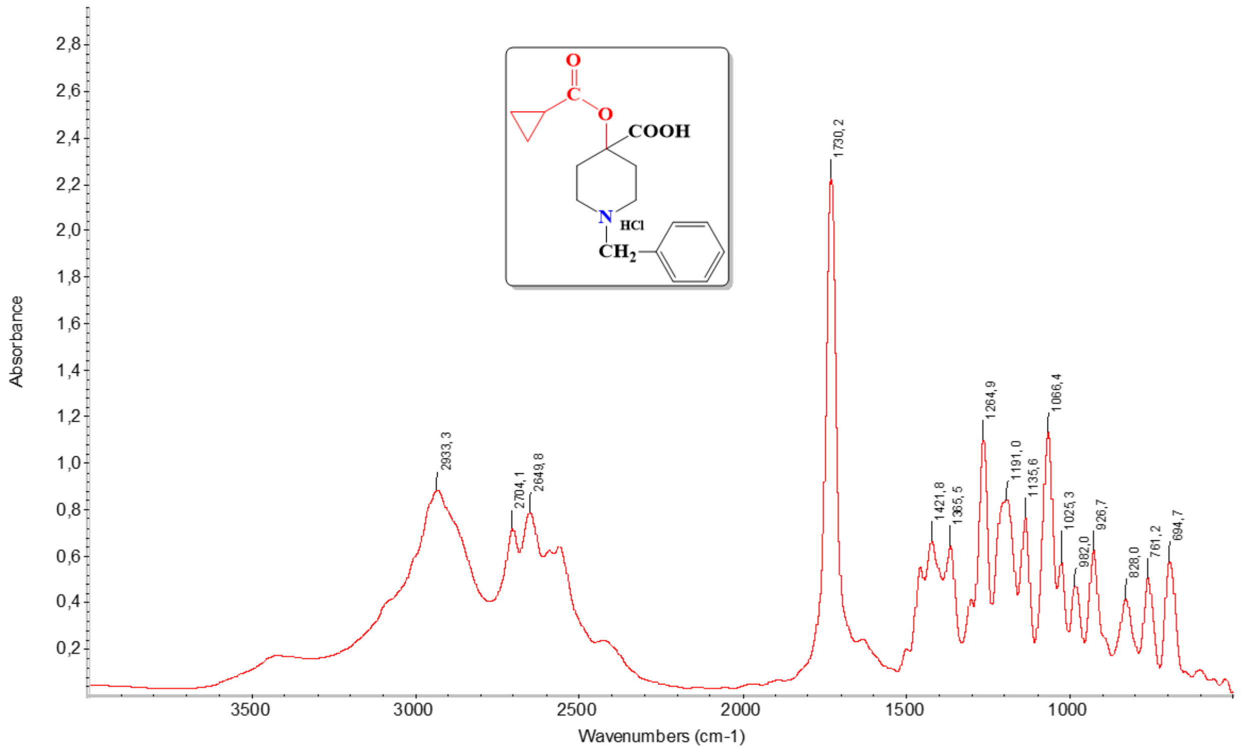

Fig. S9. IR spectra of compound **5c**

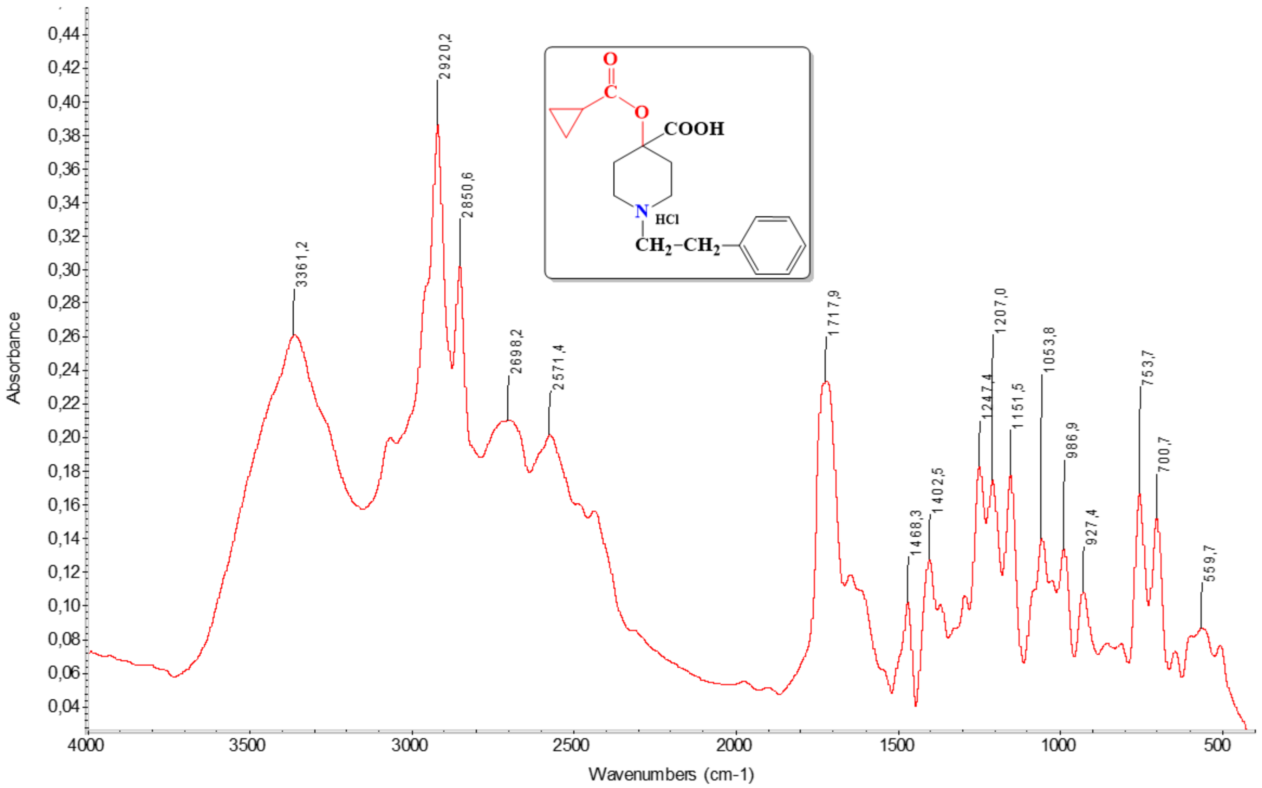

Fig. S10. IR spectra of compound **5d**

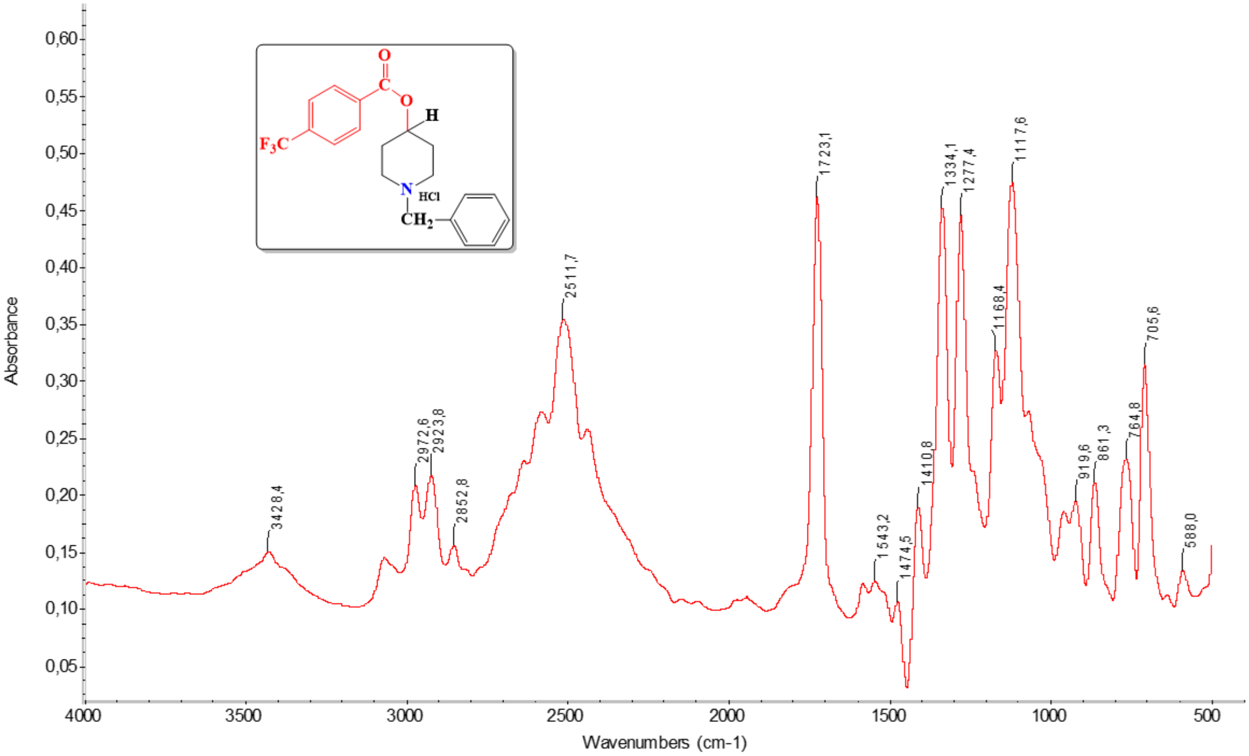

Fig. S11. IR spectra of compound **8**

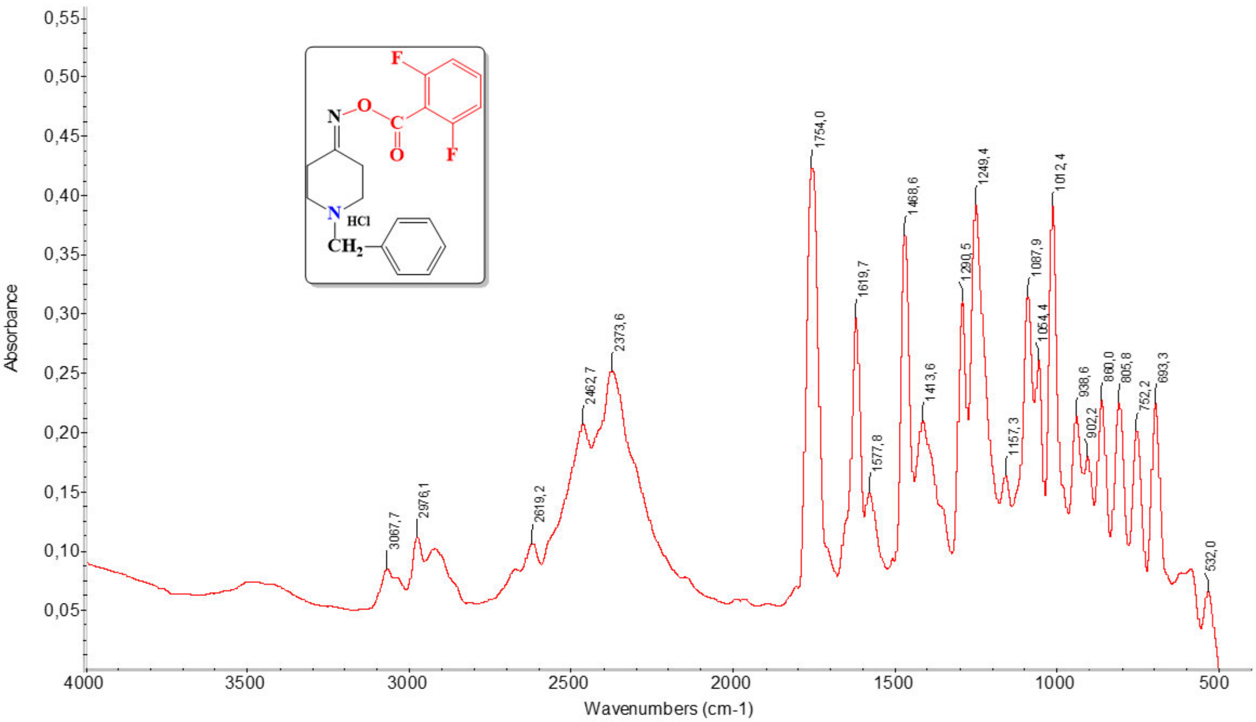

Fig. S12. IR spectra of compound **11**

Copies of NMR Spectra of Products

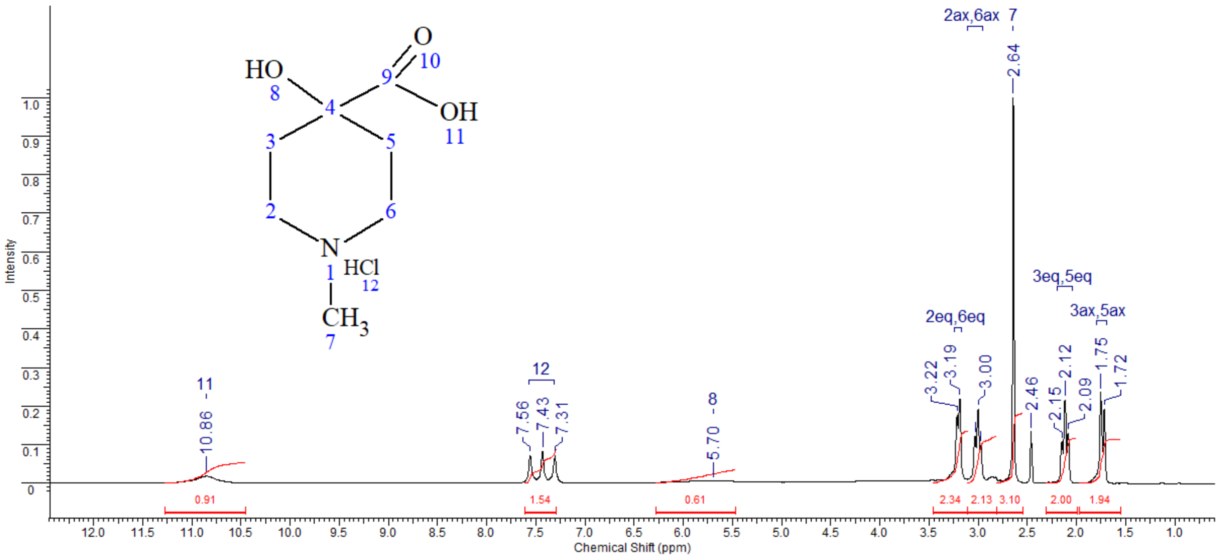

No.	(ppm)	(Hz)	Height
1	1.72	686.9	0.1912
2	1.75	700.6	0.2347
3	2.09	833.9	0.1304
4	2.12	846.3	0.2164
5	2.15	859.1	0.1070
6	2.46	983.6	0.1330
7	2.64	1056.9	1.0000
8	2.97	1188.3	0.0981
9	3.00	1200.3	0.1915
10	3.03	1212.6	0.1216
11	3.19	1274.4	0.2185
12	3.22	1285.4	0.1758
13	5.70	2276.9	0.0057
14	7.31	2920.8	0.0705
15	7.43	2971.1	0.0824
16	7.56	3021.5	0.0708
17	10.86	4339.9	0.0188

No.	Annotation	(ppm)
1	3ax,5ax	[1.70 .. 1.79]
2	3eq,5eq	[2.04 .. 2.20]
3	7	2.64
4	2ax,6ax	[2.95 .. 3.11]
5	2eq,6eq	[3.17 .. 3.24]
6	8	5.70
7	12	[7.31 .. 7.58]
8	11	10.86

No.	(ppm)	Value	Absolute Value
1	[5.47 .. 6.28]	0.609	1.26775e+1
2	[10.45 .. 11.28]	0.913	1.89965e+1
3	[7.29 .. 7.61]	1.541	3.20754e+1
4	[1.55 .. 1.97]	1.936	4.02988e+1
5	[1.99 .. 2.31]	2.000	4.16224e+1
6	[2.81 .. 3.11]	2.134	4.44053e+1
7	[3.11 .. 3.46]	2.335	4.85984e+1
8	[2.55 .. 2.81]	3.096	6.44339e+1

Fig. S13. ^{1}H NMR spectra of compound **3a** (in DMSO)

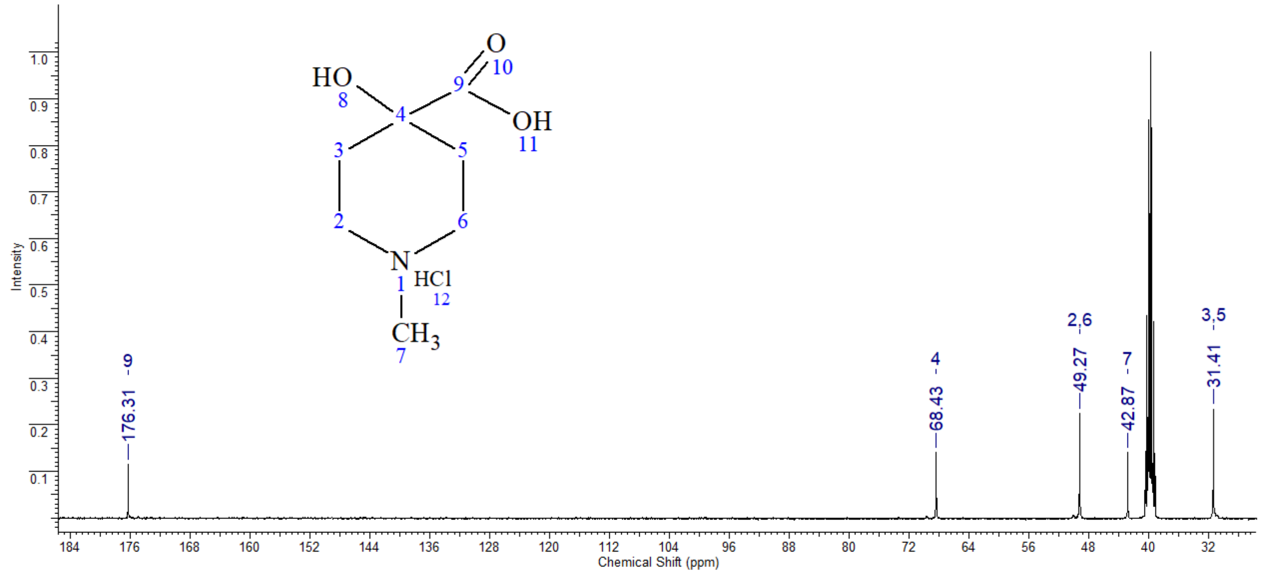

No.	(ppm)	(Hz)	Height
1	31.41	3157.3	0.2343
2	42.87	4309.4	0.1402
3	49.27	4952.5	0.2247
4	68.43	6879.0	0.1395
5	176.31	17723.1	0.1151

No.	Annotation	(ppm)
1	3,5	31.41
2	7	42.87
3	2,6	49.27
4	4	68.43
5	9	176.31

Fig. S14. ^{13}C NMR spectra of compound **3a** (in DMSO)

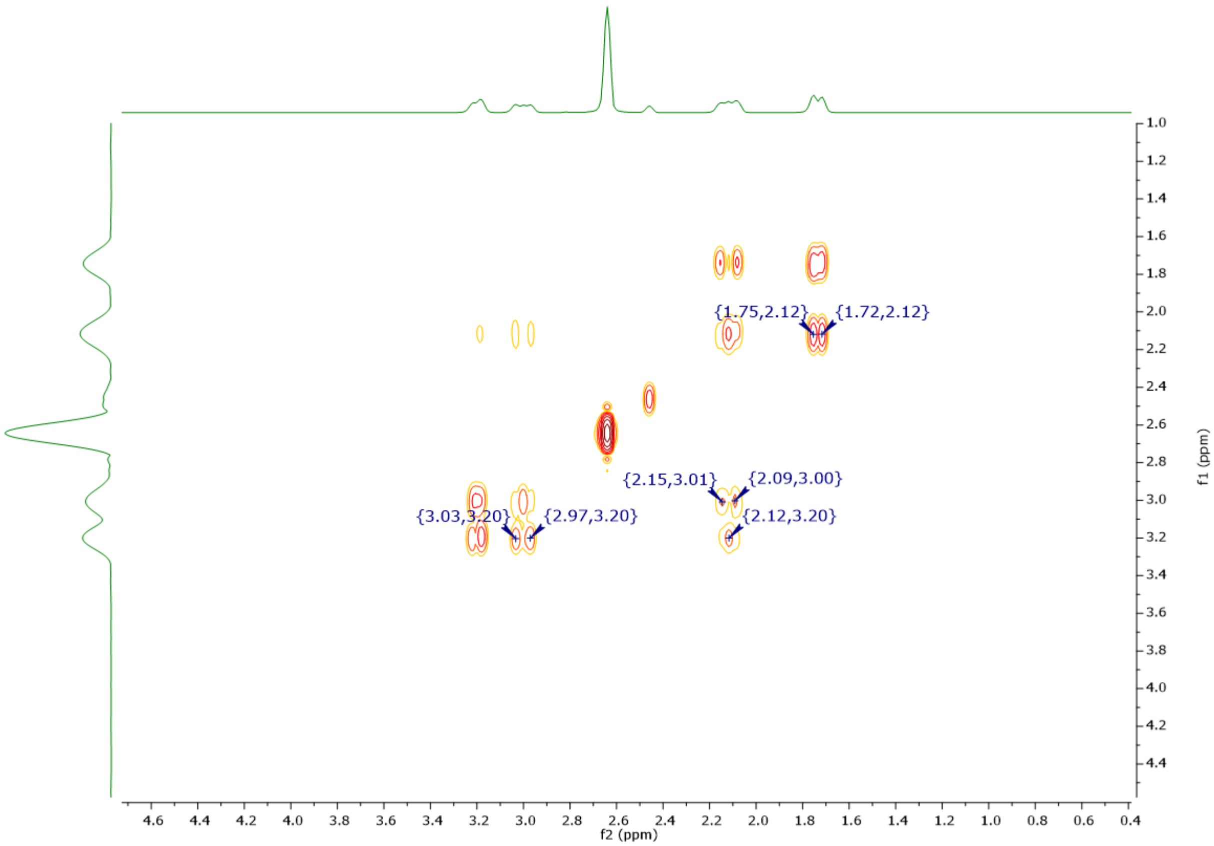

Fig. S15. COSY of compound **3a** (in DMSO)

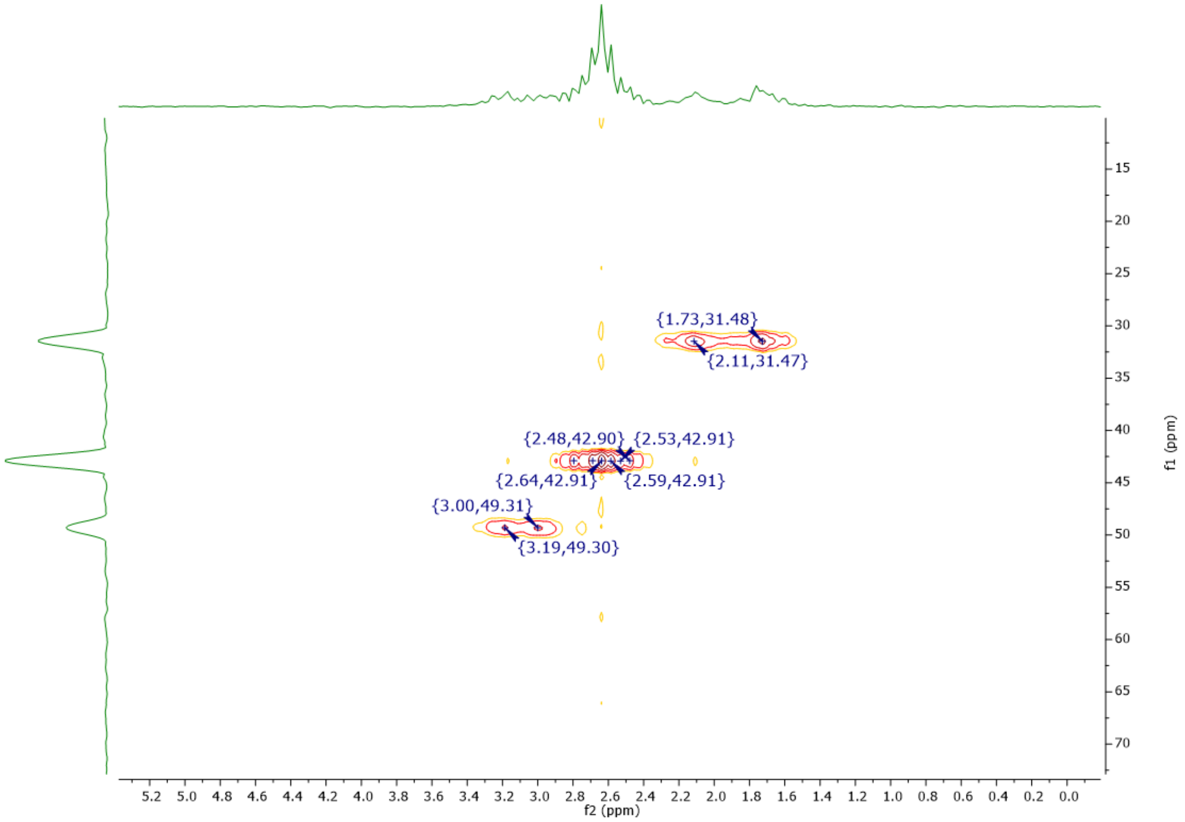

Fig. S16. HMQC of compound **3a** (in DMSO)

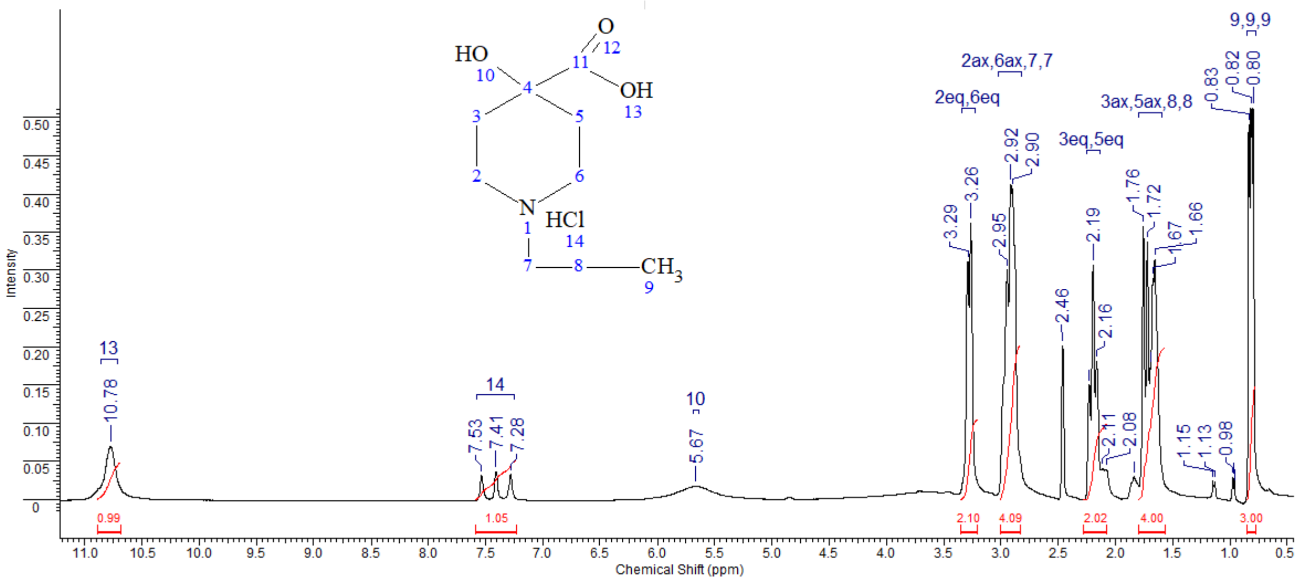

No.	(ppm)	(Hz)	Height
1	0.80	319.2	0.5317
2	0.82	326.5	1.0000
3	0.83	333.8	0.4909
4	0.96	382.4	0.0198
5	0.96	384.6	0.0279
6	0.97	388.3	0.0233
7	0.98	390.6	0.0287
8	1.13	452.9	0.0234
9	1.15	458.8	0.0238
10	1.66	662.6	0.3142
11	1.67	667.2	0.2897

No.	(ppm)	(Hz)	Height
12	1.70	677.7	0.1738
13	1.72	687.8	0.3372
14	1.76	702.0	0.3573
15	1.84	734.1	0.0295
16	2.08	832.1	0.0397
17	2.11	845.3	0.0402
18	2.16	863.7	0.1810
19	2.19	876.9	0.3072
20	2.23	890.7	0.1500
21	2.46	983.6	0.2013
22	2.90	1159.0	0.4063

No.	(ppm)	(Hz)	Height
23	2.92	1165.4	0.4118
24	2.95	1178.3	0.3001
25	3.26	1304.2	0.3611
26	3.29	1314.7	0.3112
27	5.67	2265.0	0.0176
28	7.28	2911.1	0.0328
29	7.41	2961.5	0.0365
30	7.53	3011.9	0.0314
31	10.78	4307.9	0.0698

No.	Annotation	(ppm)
1	9,9,9	[0.77 .. 0.85]
2	3ax,5ax,8,8	[1.60 .. 1.80]
3	3eq,5eq	[2.13 .. 2.25]
4	2ax,6ax,7,7	[2.82 .. 3.02]
5	2eq,6eq	[3.22 .. 3.34]
6	10	[5.64 .. 5.68]
7	14	[7.25 .. 7.57]
8	13	[10.71 .. 10.86]

No.	(ppm)	Value	Absolute Value
1	[10.68 .. 10.89]	0.994	1.35155e+1
2	[7.23 .. 7.59]	1.053	1.43162e+1
3	[2.07 .. 2.27]	2.017	2.74256e+1
4	[3.21 .. 3.36]	2.104	2.86161e+1
5	[0.78 .. 0.85]	3.001	4.08194e+1
6	[1.56 .. 1.80]	4.003	5.44467e+1
7	[2.83 .. 3.01]	4.089	5.56130e+1

Fig. S17. ^{1}H NMR spectra of compound **3b** (in DMSO)

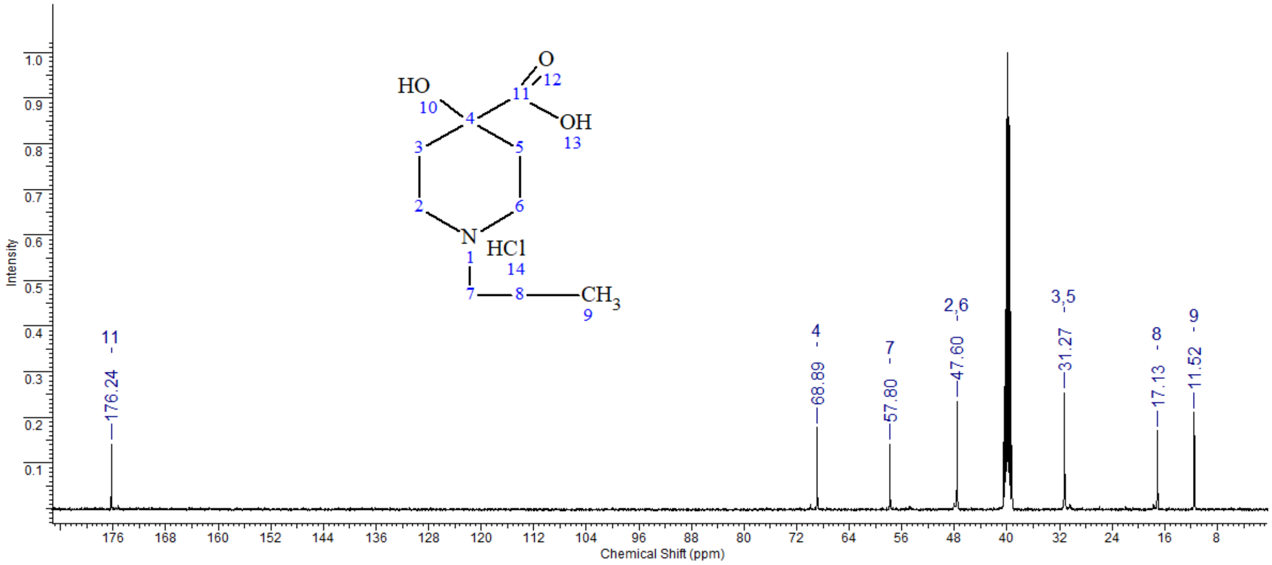

No.	(ppm)	(Hz)	Height
1	11.52	1158.0	0.2101
2	17.13	1722.5	0.1703
3	31.27	3142.9	0.2547
4	47.60	4784.8	0.2351
5	57.80	5810.4	0.1417
6	68.89	6925.1	0.1774
7	176.24	17716.4	0.1420

No.	Annotation	(ppm)
1	9	11.52
2	8	17.13
3	3,5	31.27
4	2,6	47.60
5	7	57.80
6	4	68.89
7	11	176.24

Fig. S18. ^{13}C NMR spectra of compound **3b** (in DMSO)

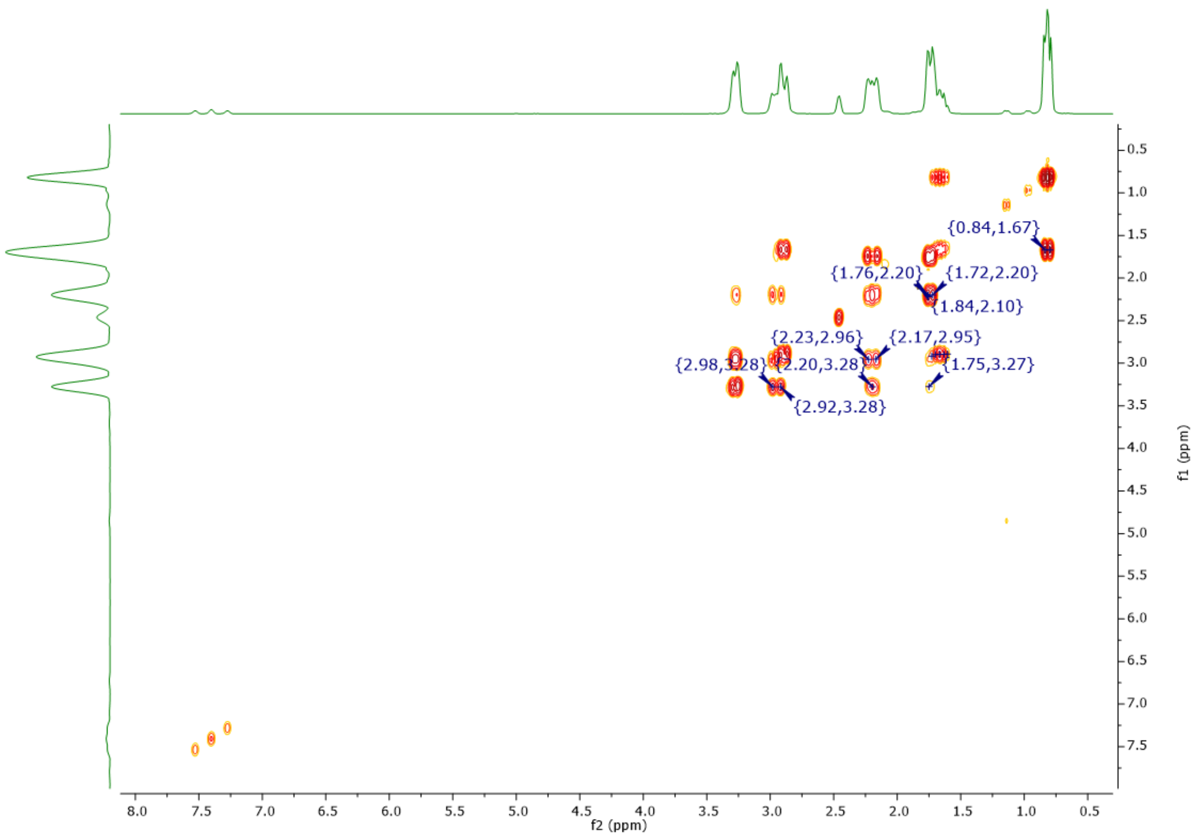

Fig. S19. COSY of compound **3b** (in DMSO)

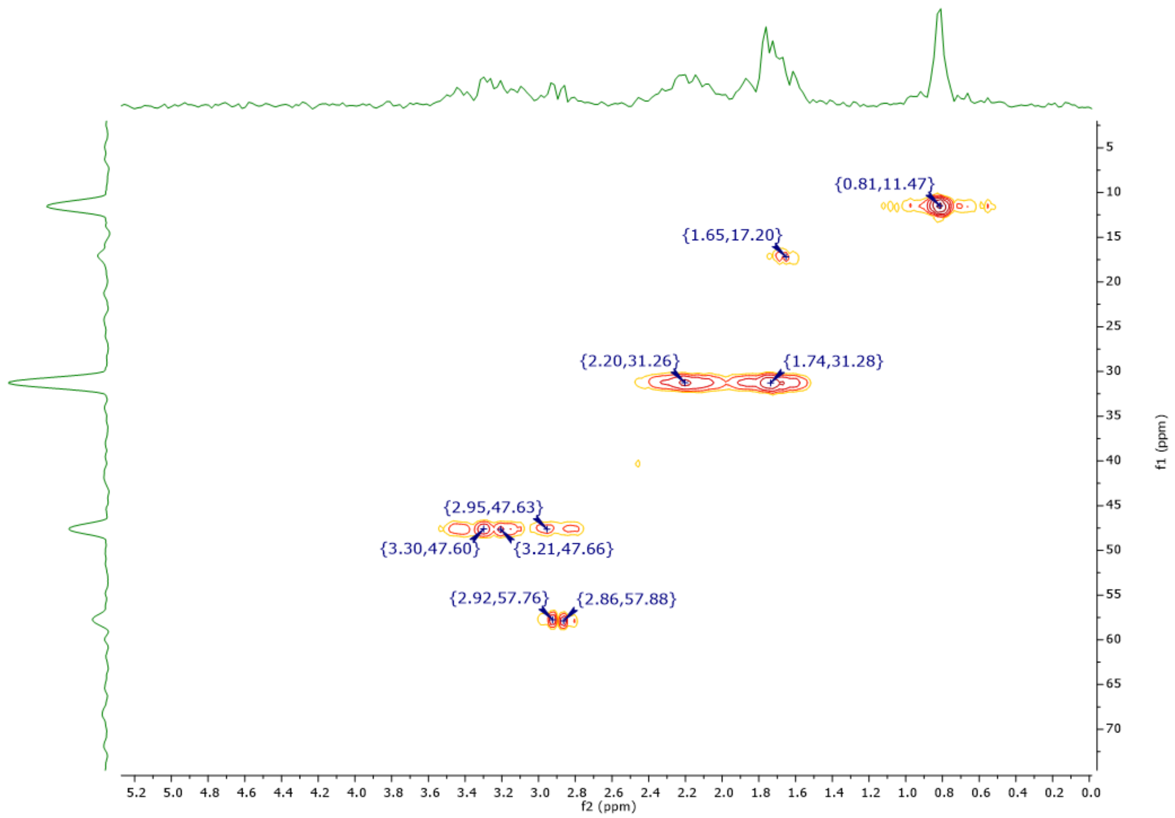

Fig. S20. HMQC of compound **3b** (in DMSO)

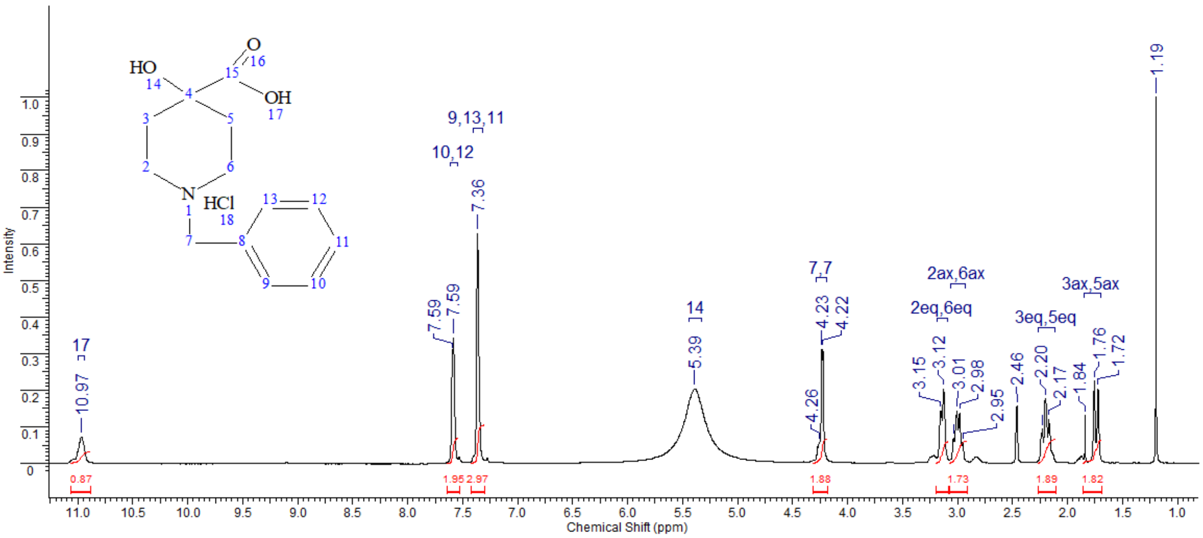

No.	(ppm)	(Hz)	Height
1	1.19	477.2	1.0000
2	1.72	688.3	0.2030
3	1.76	702.0	0.2240
4	1.84	735.4	0.1310
5	2.17	867.3	0.1192
6	2.20	877.9	0.1722
7	2.20	880.1	0.1755
8	2.23	891.1	0.0929
9	2.46	983.6	0.1564
10	2.95	1180.6	0.0557
11	2.98	1191.1	0.1367
12	3.01	1203.0	0.1442
13	3.04	1213.5	0.0691
14	3.12	1249.3	0.2028
15	3.15	1260.2	0.1428
16	4.22	1688.9	0.3116
17	4.23	1693.0	0.3121
18	4.26	1702.6	0.0543
19	5.39	2154.6	0.2025
20	7.36	2944.1	0.6283
21	7.59	3033.0	0.3431
22	7.59	3036.2	0.3023
23	10.97	4385.3	0.0721

No.	Annotation	(ppm)
1	3ax,5ax	[1.69 .. 1.85]
2	3eq,5eq	[2.12 .. 2.27]
3	2ax,6ax	[2.93 .. 3.06]
4	2eq,6eq	[3.09 .. 3.19]
5	7,7	[4.20 .. 4.29]
6	14	[5.33 .. 5.44]
7	9,13,11	[7.32 .. 7.41]
8	10,12	[7.55 .. 7.62]
9	17	[10.94 .. 11.00]

No.	(ppm)	Value	Absolute Value
1	[10.88 .. 11.06]	0.868	9.82161e+0
2	[3.08 .. 3.19]	1.674	1.89328e+1
3	[2.91 .. 3.07]	1.732	1.95846e+1
4	[1.69 .. 1.86]	1.821	2.05928e+1
5	[4.18 .. 4.31]	1.885	2.13157e+1
6	[2.11 .. 2.27]	1.887	2.13382e+1
7	[7.53 .. 7.64]	1.951	2.20616e+1
8	[7.30 .. 7.42]	2.975	3.36437e+1

Fig. S21. ^{1}H NMR spectra of compound **3c** (in DMSO)

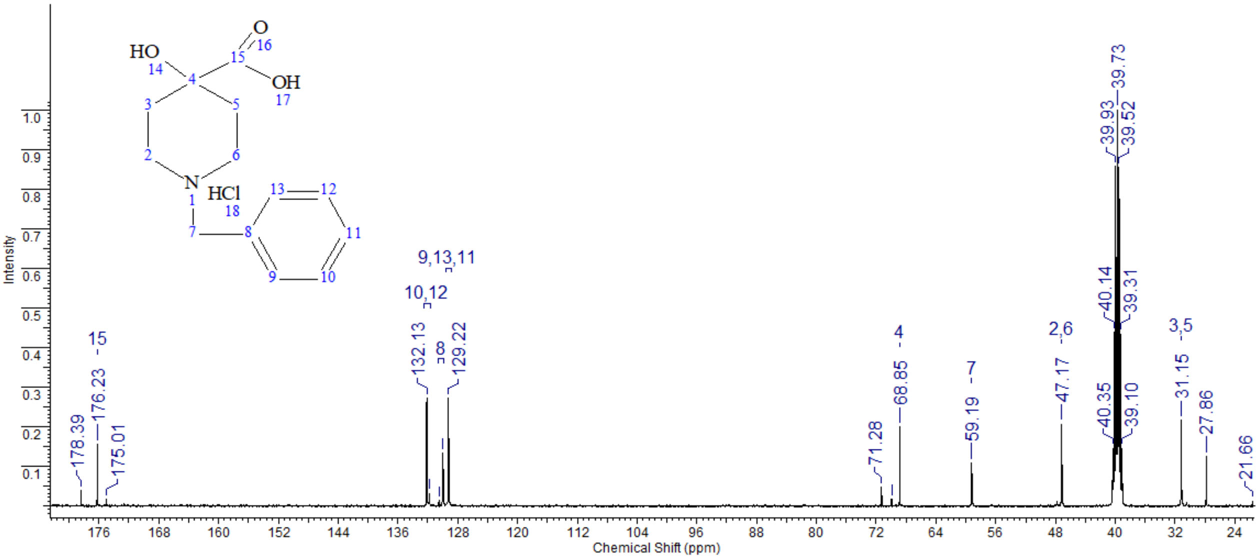

No.	(ppm)	(Hz)	Height
1	21.66	2177.8	0.0098
2	27.86	2800.8	0.1230
3	31.15	3131.4	0.2142
4	39.10	3930.8	0.1432
5	39.31	3951.9	0.4336
6	39.52	3973.0	0.8535
7	39.73	3994.1	1.0000
8	39.93	4014.2	0.8572
9	40.14	4035.3	0.4367
10	40.35	4056.4	0.1435
11	47.17	4741.7	0.2060
12	59.19	5950.3	0.1070
13	68.85	6921.2	0.1978
14	69.95	7031.4	0.0149
15	71.28	7165.6	0.0453
16	129.22	12990.2	0.2695
17	129.97	13065.0	0.1316
18	130.05	13073.6	0.1275
19	130.49	13117.7	0.0114
20	131.80	13249.0	0.0296
21	132.13	13282.6	0.2706
22	175.01	17592.8	0.0146
23	176.23	17715.5	0.1537
24	178.39	17933.0	0.0370

No.	Annotation	(ppm)
1	3,5	31.15
2	2,6	47.17
3	7	59.19
4	4	[68.84 .. 68.88]
5	9,13,11	[128.78 .. 129.56]
6	8	[129.81 .. 130.61]
7	10,12	[131.71 .. 132.46]
8	15	176.23

Fig. S22. ^{13}C NMR spectra of compound **3c** (in DMSO)

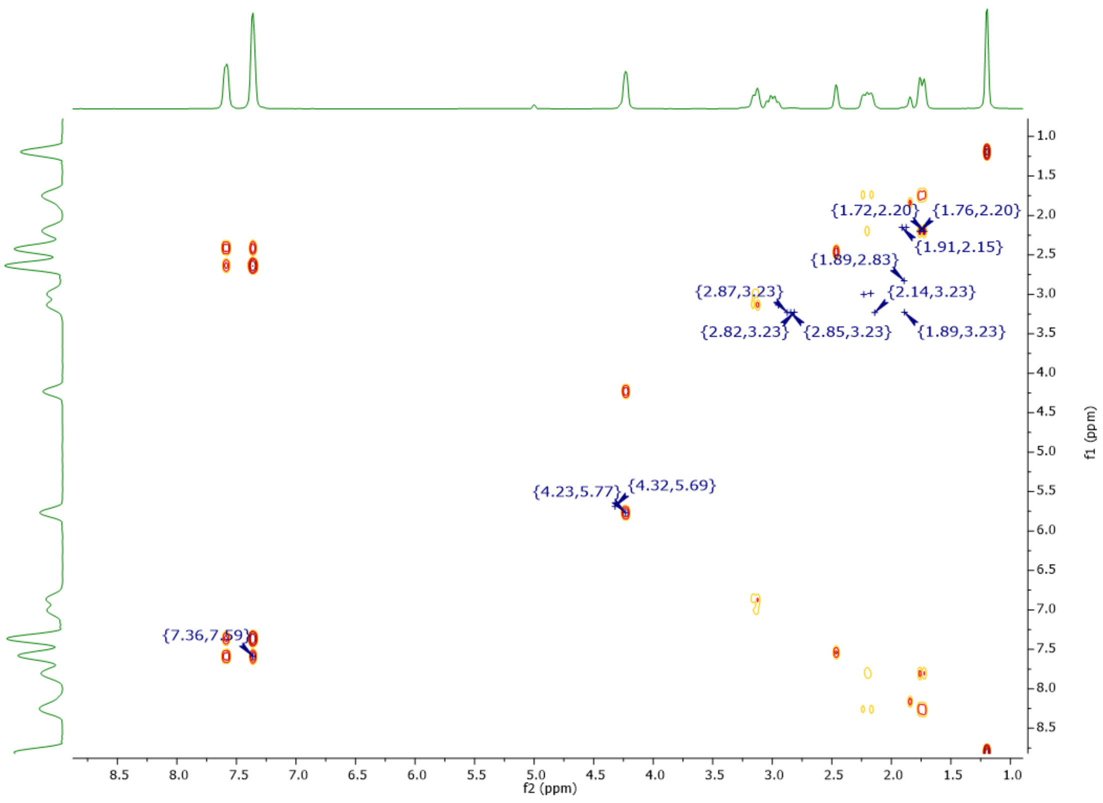

Fig. S23. COSY spectra of compound **3c** (in DMSO)

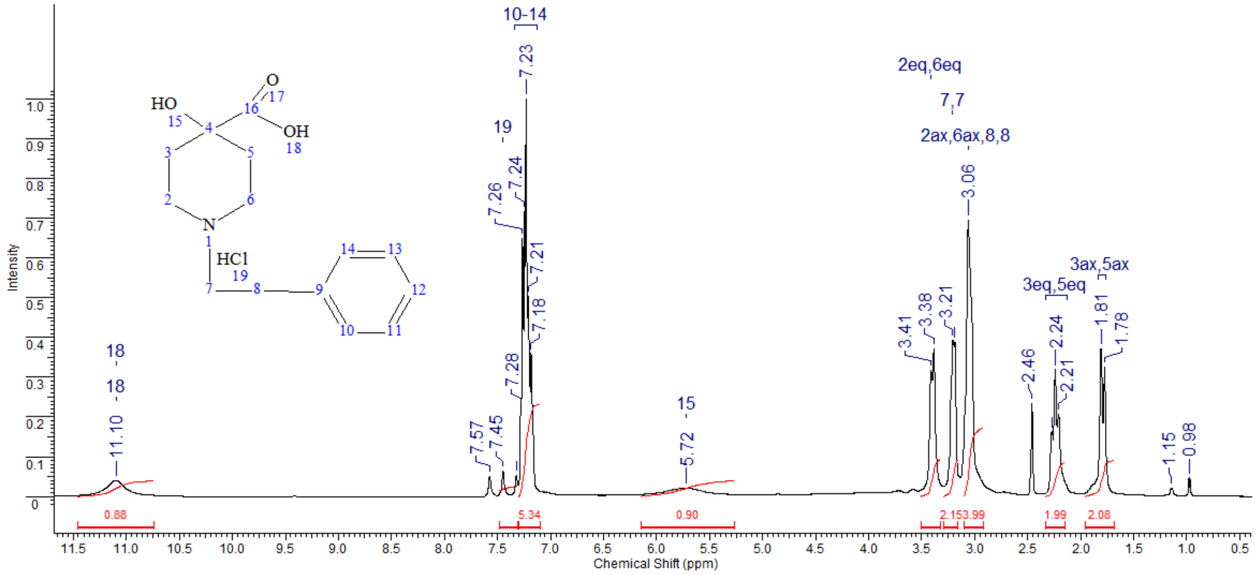

No.	(ppm)	(Hz)	Height
1	0.98	392.0	0.0444
2	1.15	459.3	0.0209
3	1.78	709.8	0.3245
4	1.81	723.1	0.3711
5	2.21	883.4	0.2078
6	2.24	896.2	0.3190
7	2.27	908.5	0.1627
8	2.46	983.6	0.2331
9	3.06	1224.1	0.6962
10	3.19	1274.4	0.3889
11	3.21	1281.8	0.3937
12	3.38	1353.2	0.3717

No.	(ppm)	(Hz)	Height
13	3.41	1363.3	0.3174
14	5.72	2286.5	0.0211
15	7.18	2871.3	0.3595
16	7.21	2882.7	0.5028
17	7.23	2890.1	1.0000
18	7.24	2896.0	0.7163
19	7.26	2903.8	0.6492
20	7.28	2910.7	0.2205
21	7.32	2926.3	0.0527
22	7.45	2977.1	0.0633
23	7.57	3027.5	0.0507
24	11.10	4435.6	0.0394

No.	Annotation	(ppm)
1	3ax,5ax	[1.77 .. 1.84]
2	3eq,5eq	[2.13 .. 2.33]
3	2ax,6ax,8,8	3.06
4	7,7	3.21
5	2eq,6eq	3.41
6	15	5.72
7	10-14	[7.13 .. 7.34]
8	19	7.45
9	18	11.10
10	18	11.10

No.	(ppm)	Value	Absolute Value
1	[7.31 .. 7.48]	0.324	4.65748e+0
2	[10.74 .. 11.46]	0.881	1.26634e+1
3	[5.26 .. 6.15]	0.897	1.28987e+1
4	[2.15 .. 2.33]	1.990	2.86165e+1
5	[1.68 .. 1.96]	2.085	2.99845e+1
6	[3.32 .. 3.50]	2.119	3.04738e+1
7	[3.16 .. 3.29]	2.147	3.08821e+1
8	[2.92 .. 3.10]	3.993	5.74210e+1
9	[7.10 .. 7.30]	5.338	7.67604e+1

Fig. S24. ^{1}H NMR spectra of compound **3d** (in DMSO)

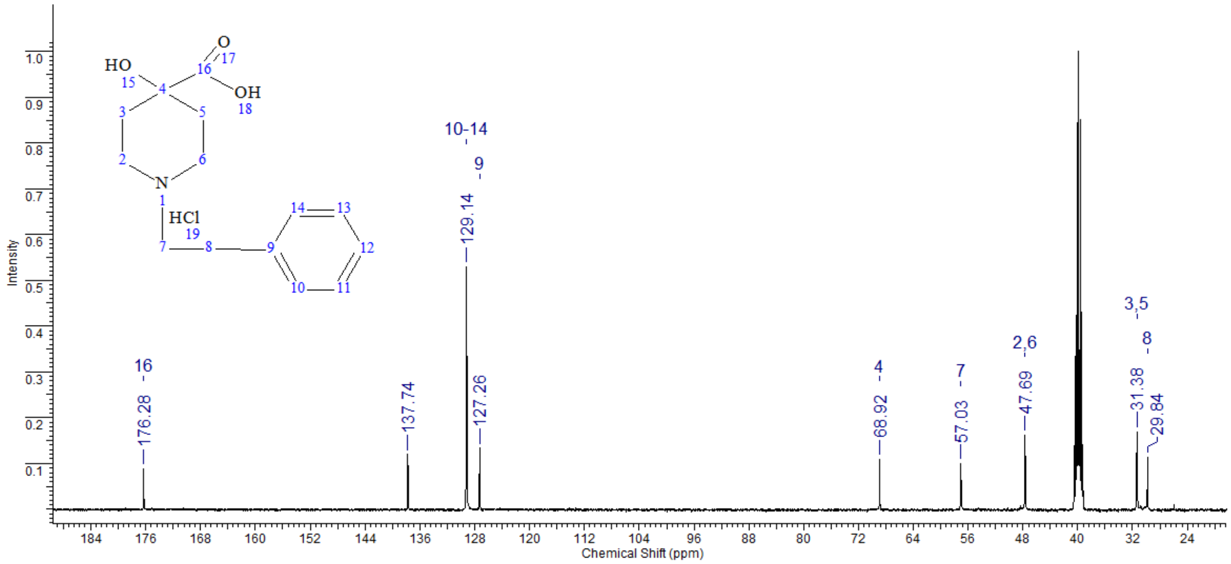

No.	(ppm)	(Hz)	Height
1	29.84	3000.1	0.1144
2	31.38	3154.4	0.1689
3	47.69	4794.4	0.1614
4	57.03	5732.7	0.0987
5	68.92	6927.9	0.1093
6	127.26	12792.8	0.1336
7	129.14	12981.6	0.5299
8	137.74	13846.1	0.1191
9	176.28	17720.3	0.0881

No.	Annotation	(ppm)
1	8	29.84
2	3,5	31.38
3	2,6	47.69
4	7	57.03
5	4	68.92
6	9	127.26
7	10-14	129.14
8	16	176.28

Fig. S25. ^{13}C NMR spectra of compound **3d** (in DMSO)

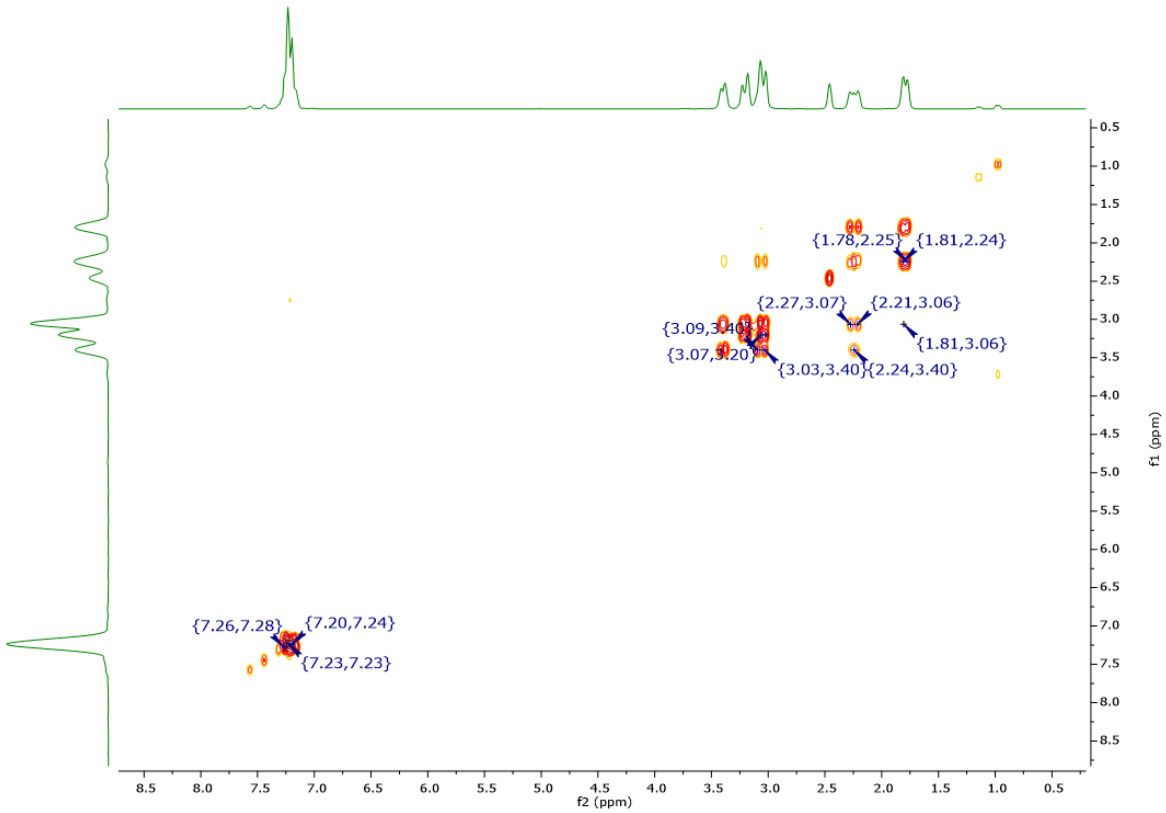

Fig. S26. COSY of compound **3d** (in DMSO)

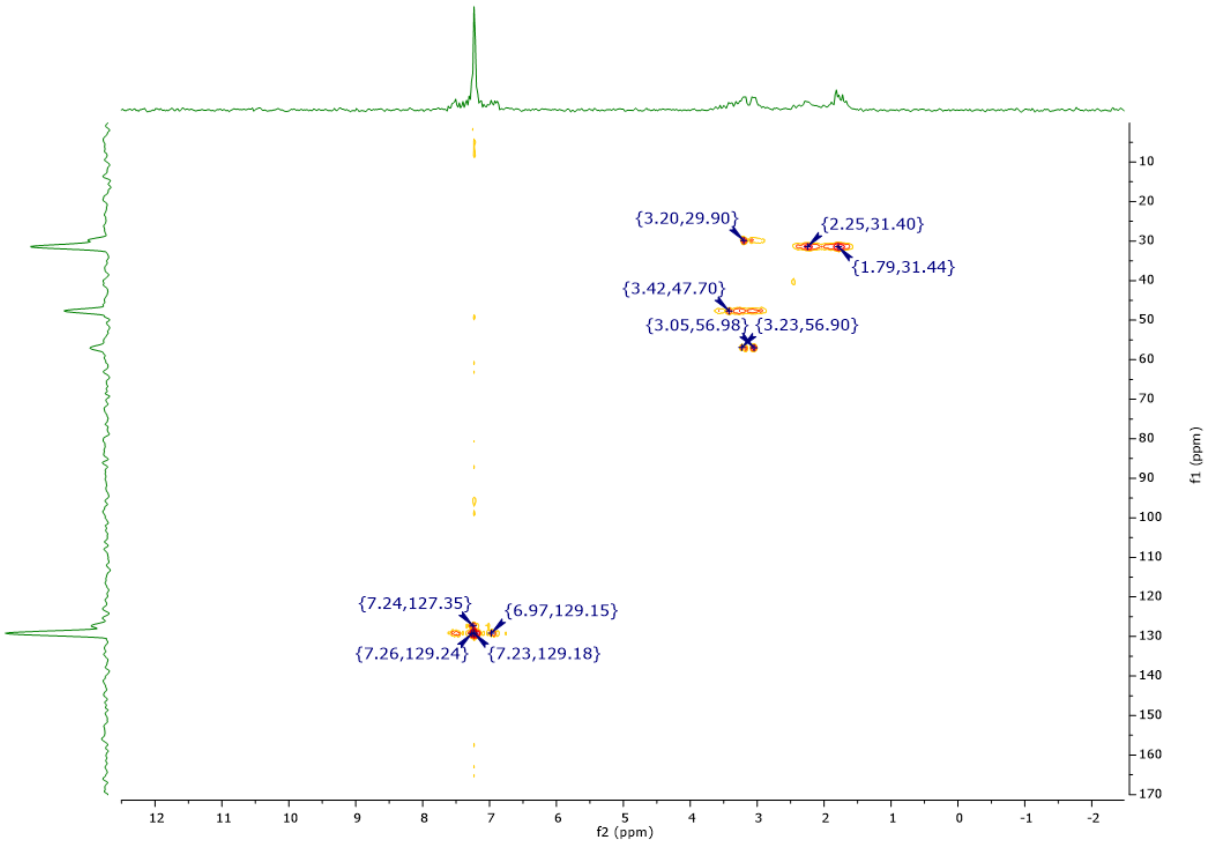

Fig. S27. HMQC of compound **3d** (in DMSO)

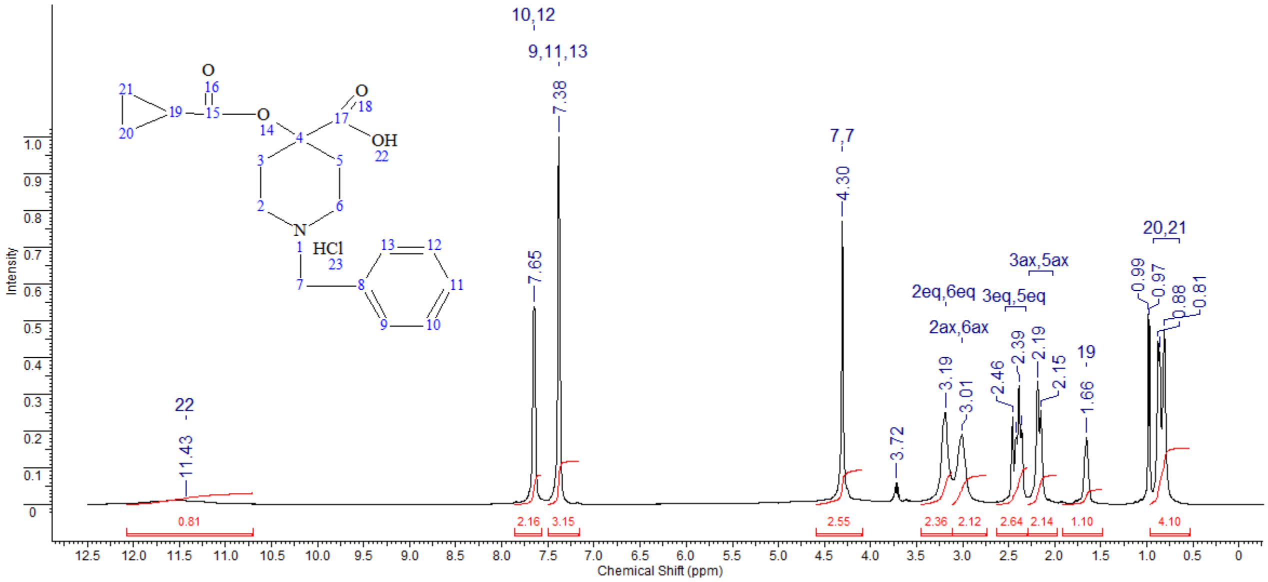

No.	(ppm)	(Hz)	Height
1	0.81	325.1	0.4748
2	0.86	343.0	0.4163
3	0.88	350.3	0.4443
4	0.97	388.3	0.5037
5	0.99	394.3	0.5193
6	1.66	662.2	0.1826
7	2.15	860.0	0.2553
8	2.19	873.7	0.3348
9	2.36	943.8	0.2037
10	2.39	955.3	0.3233
11	2.42	966.7	0.1831
12	2.46	983.2	0.2393
13	3.01	1202.5	0.1901
14	3.19	1274.4	0.2504
15	3.72	1486.5	0.0588
16	4.30	1720.9	0.7699
17	7.38	2951.0	1.0000
18	7.65	3057.7	0.5393
19	11.43	4570.7	0.0105

No.	Annotation	(ppm)
1	20,21	[0.65 .. 0.93]
2	19	1.66
3	3ax,5ax	[2.02 .. 2.28]
4	3eq,5eq	[2.32 .. 2.53]
5	2ax,6ax	3.01
6	2eq,6eq	3.19
7	7,7	4.30
8	9,11,13	7.38
9	10,12	7.65
10	22	11.43

No.	(ppm)	Value	Absolute Value
1	[10.70 .. 12.07]	0.815	1.04727e+1
2	[1.48 .. 1.91]	1.097	1.40971e+1
3	[2.74 .. 3.11]	2.123	2.72844e+1
4	[1.97 .. 2.29]	2.143	2.75349e+1
5	[7.56 .. 7.86]	2.160	2.77600e+1
6	[3.11 .. 3.46]	2.357	3.02934e+1
7	[4.09 .. 4.59]	2.546	3.27226e+1
8	[2.30 .. 2.63]	2.635	3.38655e+1
9	[7.16 .. 7.50]	3.154	4.05352e+1
10	[0.53 .. 0.96]	4.096	5.26330e+1

Fig. S28. ^{1}H NMR spectra of compound **5c** (in DMSO)

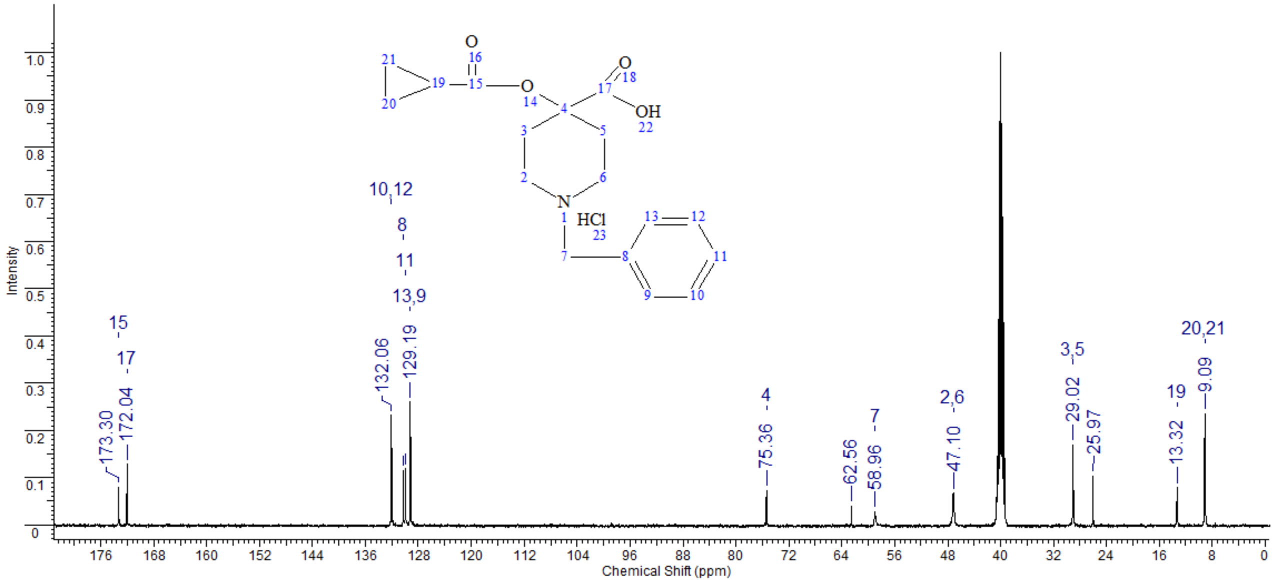

No.	(ppm)	(Hz)	Height
1	9.09	913.6	0.2344
2	13.32	1339.1	0.0787
3	25.97	2611.0	0.1032
4	29.02	2917.7	0.1696
5	47.10	4735.0	0.0672
6	58.96	5927.3	0.0276
7	62.56	6288.6	0.0395

No.	(ppm)	(Hz)	Height
8	75.36	7575.9	0.0725
9	129.19	12987.4	0.2597
10	129.92	13060.2	0.1192
11	130.26	13094.7	0.1157
12	132.06	13275.9	0.2313
13	172.04	17294.7	0.1277
14	173.30	17421.2	0.0792

No.	Annotation	(ppm)
1	20,21	9.09
2	19	13.32
3	3,5	29.02
4	2,6	47.10
5	7	58.96
6	4	75.36
7	13,9	129.19
8	11	129.92
9	8	130.26
10	10,12	132.06
11	17	172.04
12	15	173.30

Fig. S29. ^{13}C NMR spectra of compound **5c** (in DMSO)

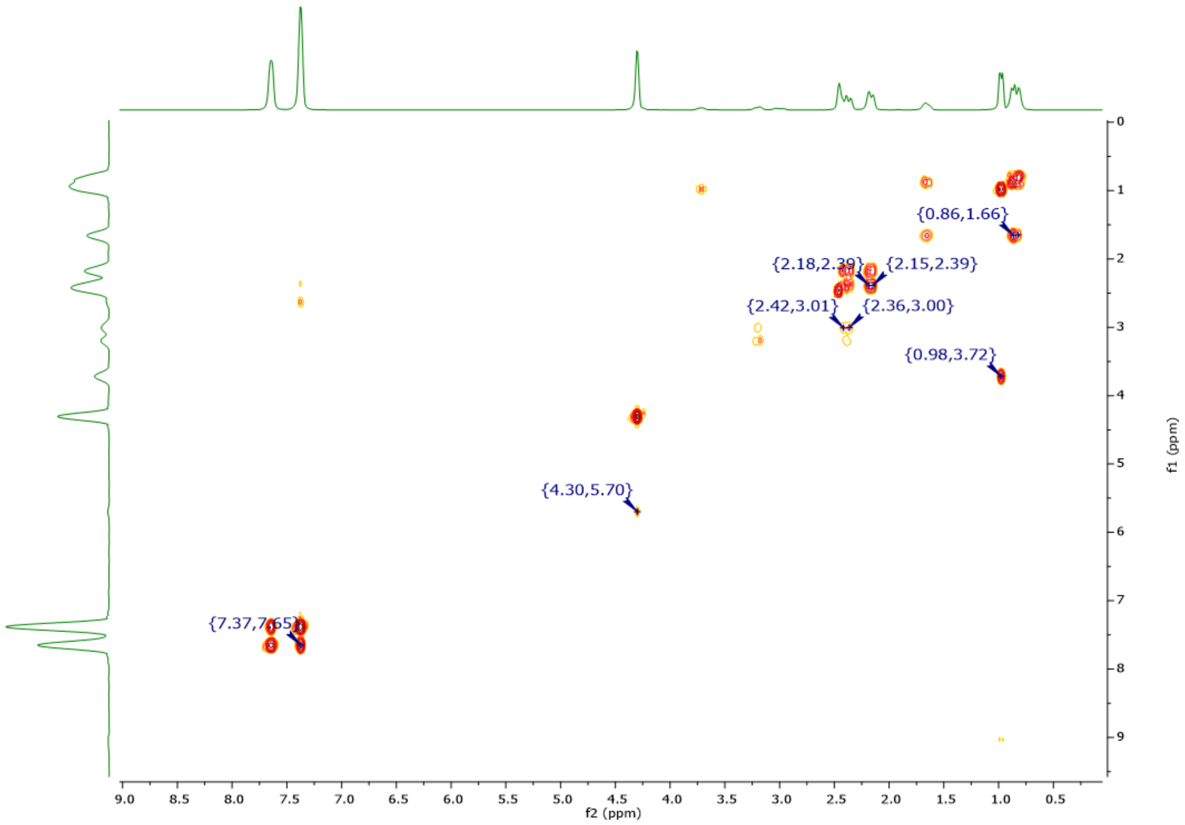

Fig. S30. COSY of compound **5c** (in DMSO)

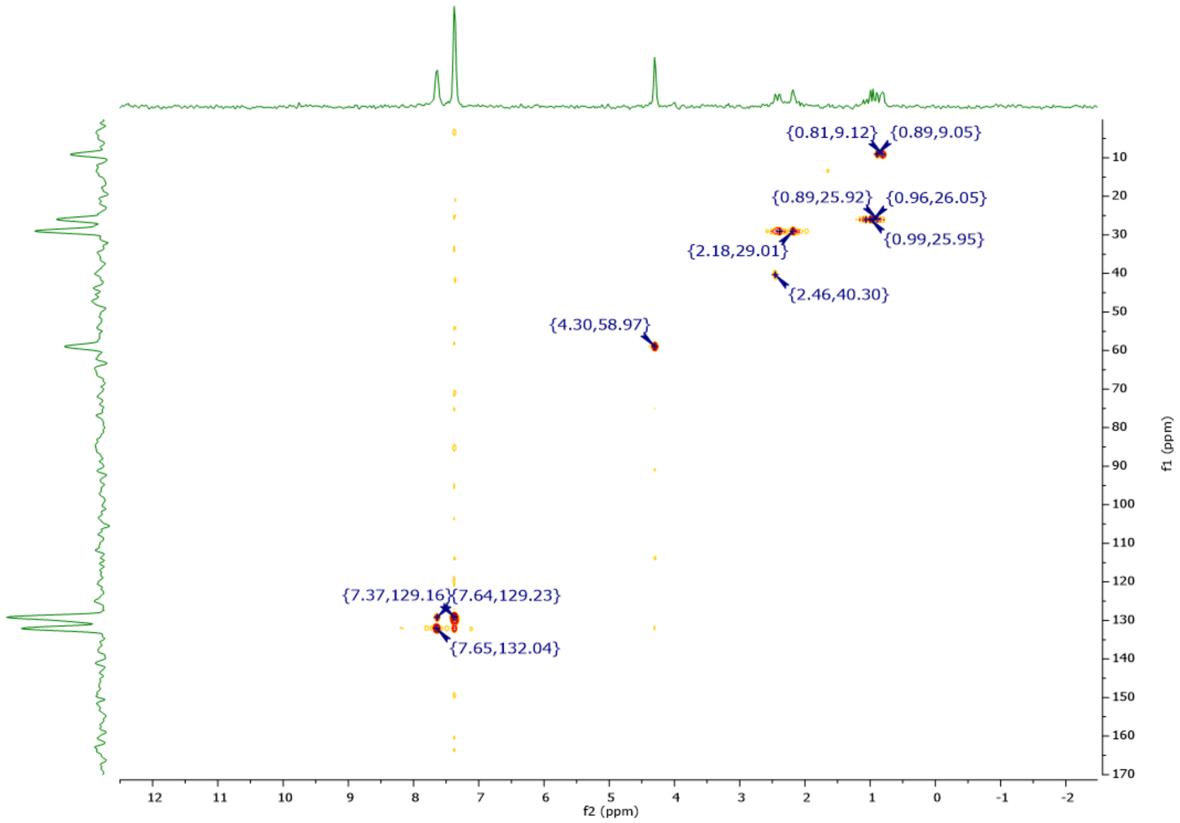

Fig. S31. HMQC of compound **5c** (in DMSO)

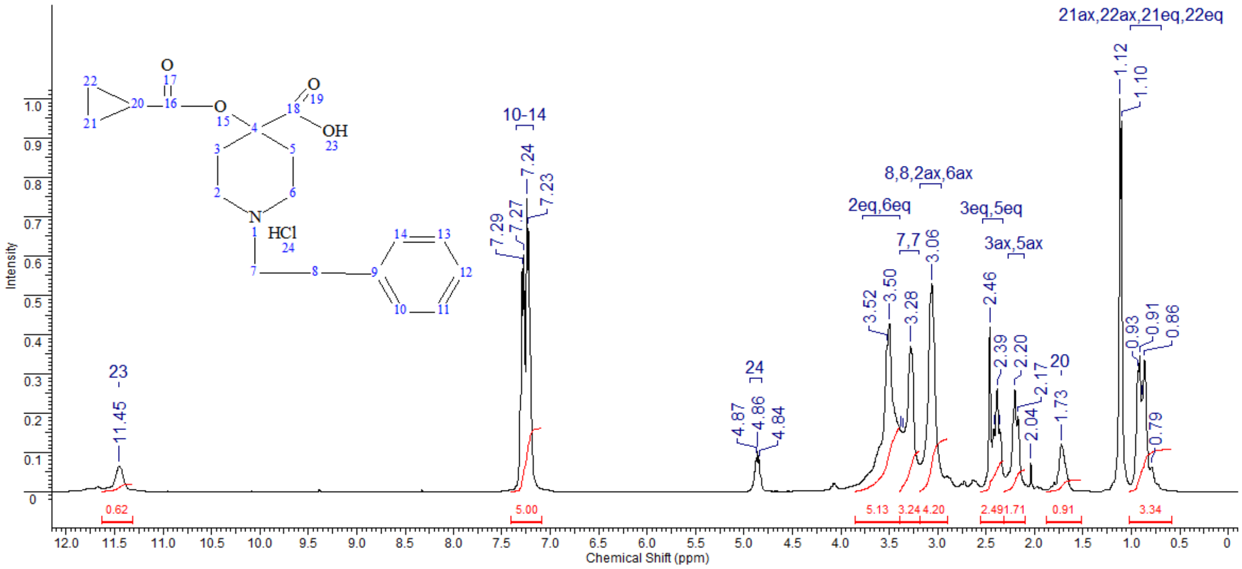

No.	(ppm)	(Hz)	Height
1	0.79	315.5	0.0620
2	0.86	344.8	0.3354
3	0.89	356.3	0.2347
4	0.91	365.0	0.3451
5	0.93	372.3	0.3086
6	1.10	440.1	0.9430
7	1.12	446.0	1.0000
8	1.73	690.1	0.1204
9	2.04	813.7	0.0723
10	2.17	866.9	0.1915
11	2.20	881.1	0.2596
12	2.36	942.0	0.1583
13	2.39	954.3	0.2604
14	2.42	967.2	0.1601
15	2.46	984.1	0.4191
16	3.06	1222.2	0.5289
17	3.28	1311.1	0.3716
18	3.36	1341.3	0.1522
19	3.37	1349.1	0.1551
20	3.50	1399.0	0.4279
21	3.52	1408.2	0.3731
22	4.84	1936.2	0.0798
23	4.86	1942.1	0.1033
24	4.87	1948.1	0.0867
25	7.23	2888.7	0.6711
26	7.24	2896.0	0.7461
27	7.27	2907.9	0.6027
28	7.29	2914.8	0.5676
29	11.45	4578.5	0.0660

No.	Annotation	(ppm)
1	21ax,22ax,21eq,22eq	[0.70 .. 1.01]
2	20	1.73
3	3ax,5ax	[2.11 .. 2.27]
4	3eq,5eq	[2.33 .. 2.53]
5	8,8,2ax,6ax	[2.96 .. 3.18]
6	7,7	[3.19 .. 3.39]
7	2eq,6eq	[3.39 .. 3.77]
8	24	[4.81 .. 4.94]
9	10-14	[7.17 .. 7.36]
10	23	11.45

No.	(ppm)	Value	Absolute Value
1	[11.31 .. 11.63]	0.621	6.12210e+0
2	[1.52 .. 1.87]	0.912	8.99369e+0
3	[2.10 .. 2.31]	1.711	1.68684e+1
4	[2.31 .. 2.55]	2.488	2.45313e+1
5	[3.19 .. 3.39]	3.244	3.19837e+1
6	[0.58 .. 1.03]	3.335	3.28814e+1
7	[2.90 .. 3.19]	4.202	4.14227e+1
8	[7.09 .. 7.40]	5.004	4.93349e+1
9	[3.39 .. 3.86]	5.129	5.05654e+1

Fig. S32. ^{1}H NMR spectra of compound **5d** (in DMSO)

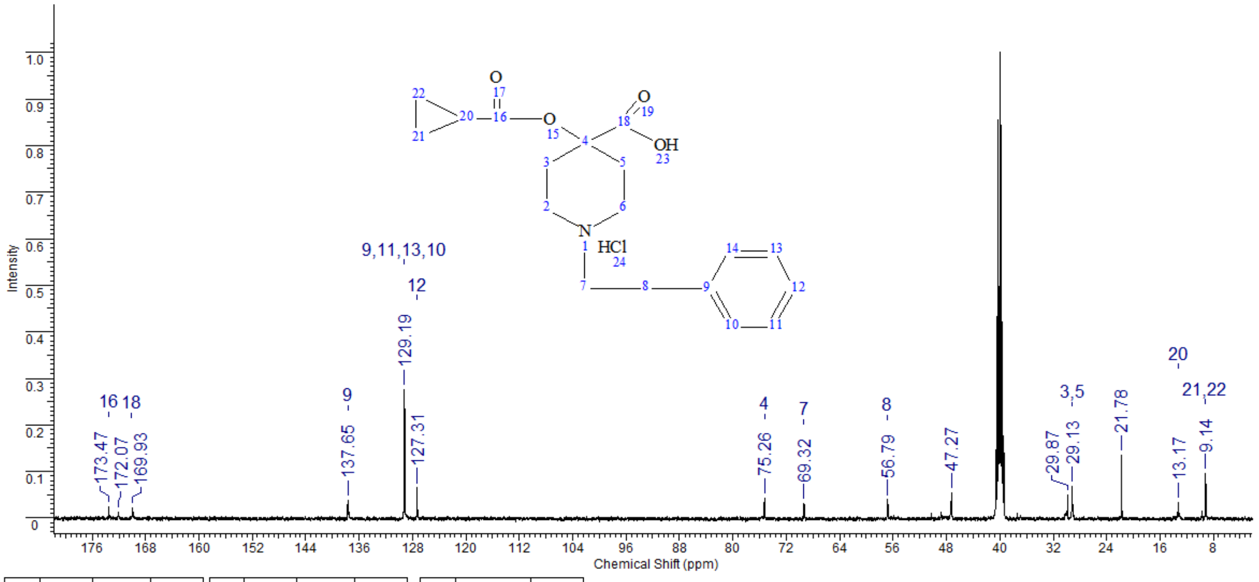

No.	(ppm)	(Hz)	Height
1	9.14	919.3	0.0955
2	13.17	1323.8	0.0331
3	21.78	2189.3	0.1349
4	29.13	2928.2	0.0683
5	29.87	3003.0	0.0494
6	47.27	4752.2	0.0545
7	56.79	5708.8	0.0392
8	69.32	6968.2	0.0312

No.	(ppm)	(Hz)	Height
9	75.26	7565.3	0.0429
10	127.31	12797.6	0.0654
11	129.19	12986.4	0.2757
12	137.65	13837.5	0.0381
13	169.93	17081.9	0.0223
14	172.07	17297.6	0.0134
15	173.47	17438.5	0.0249

No.	Annotation	(ppm)
1	21,22	9.14
2	20	13.17
3	3,5	29.13
4	8	56.79
5	7	69.32
6	4	75.26
7	12	127.31
8	9,11,13,10	129.19
9	9	137.65
10	18	170.06
11	16	173.47

Fig. S33. ^{13}C NMR spectra of compound **5d** (in DMSO)

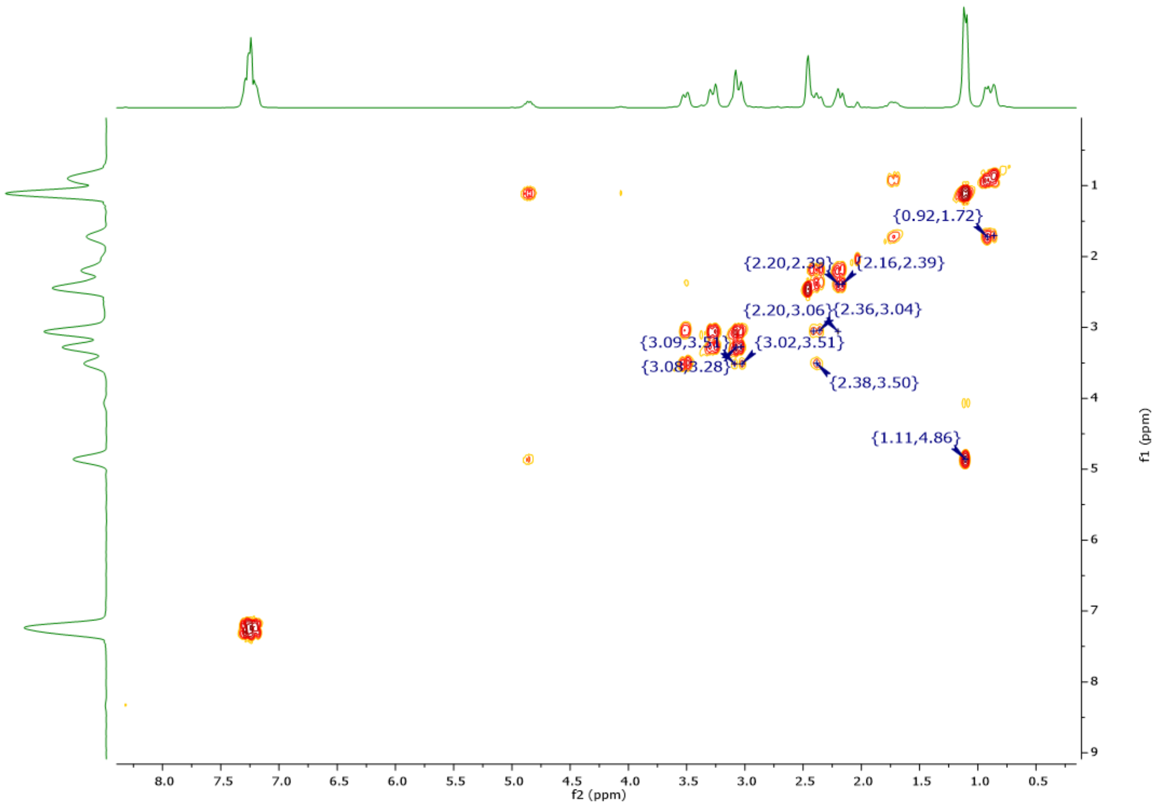

Fig. S34. COSY of compound **5d** (in DMSO)

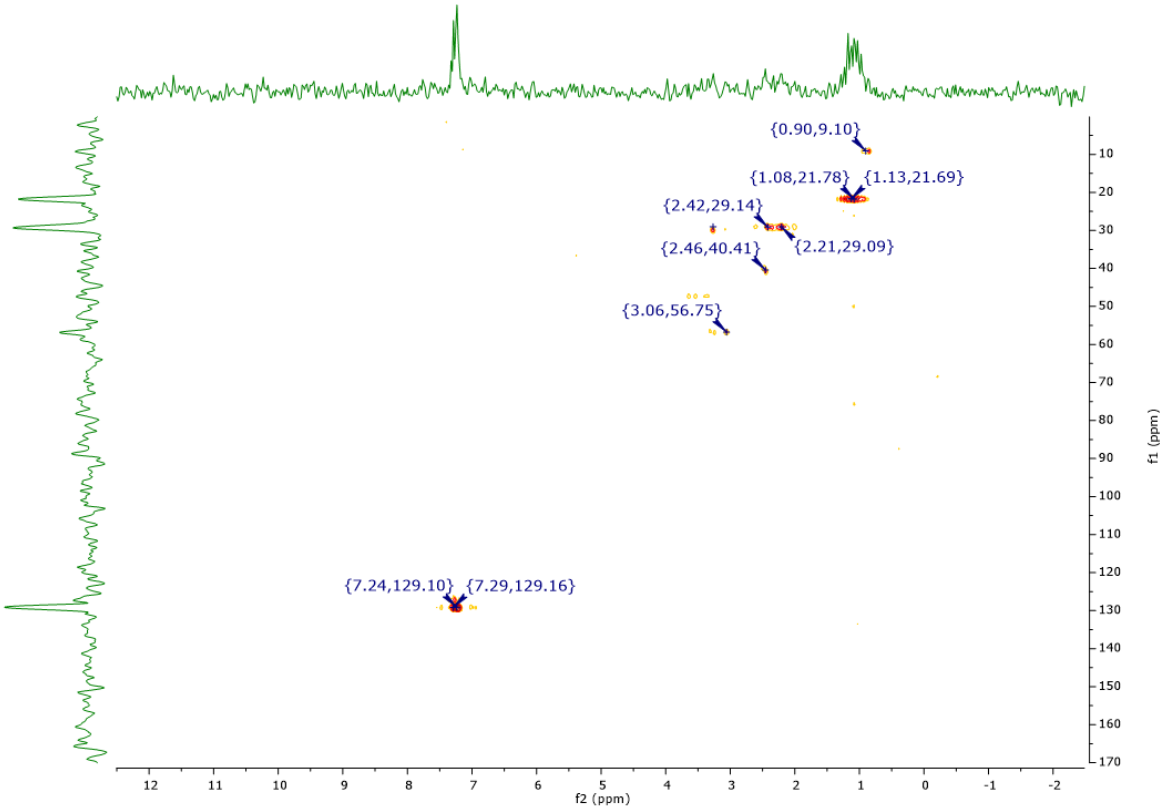

Fig. S35. HMQC of compound **5d** (in DMSO)

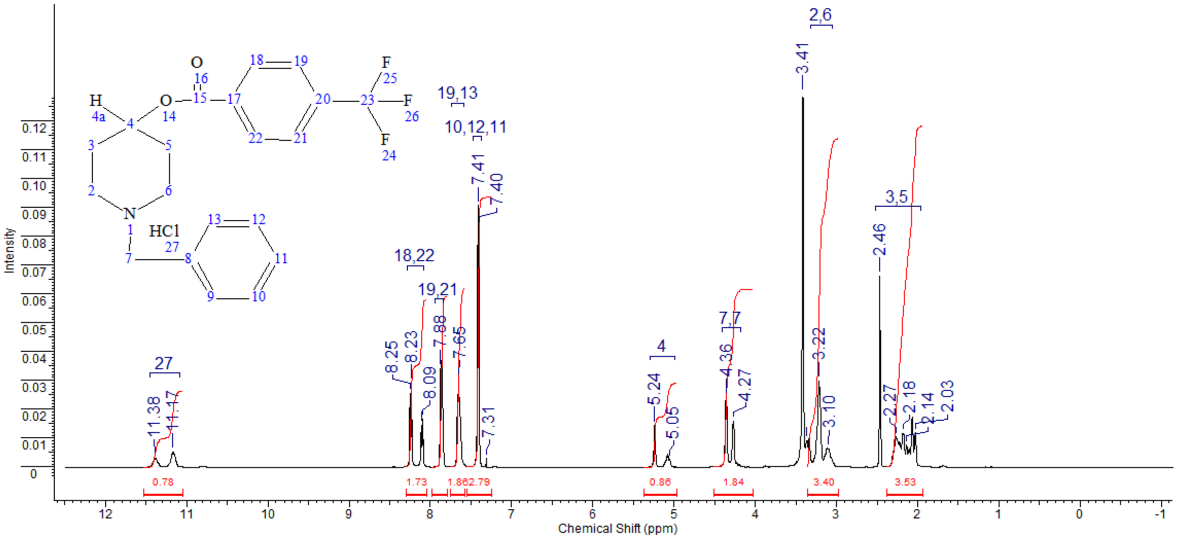

No.	(ppm)	(Hz)	Height
1	2.03	810.5	0.0118
2	2.14	853.6	0.0076
3	2.18	870.1	0.0116
4	2.27	906.2	0.0104
5	2.46	984.6	0.0663
6	3.10	1237.8	0.0064
7	3.22	1285.9	0.0299
8	3.36	1345.0	0.0092
9	3.41	1365.1	1.0000
10	4.27	1706.3	0.0158
11	4.36	1741.1	0.0242
12	5.05	2019.5	0.0026
13	5.24	2094.6	0.0143

No.	(ppm)	(Hz)	Height
14	7.31	2923.0	0.0030
15	7.40	2960.1	0.0835
16	7.41	2962.9	0.0907
17	7.42	2966.6	0.0770
18	7.65	3057.7	0.0305
19	7.88	3148.8	0.0342
20	8.09	3234.9	0.0166
21	8.11	3243.2	0.0145
22	8.23	3290.8	0.0292
23	8.25	3299.0	0.0256
24	11.17	4464.5	0.0053
25	11.38	4551.5	0.0031

No.	Annotation	(ppm)
1	3,5	[1.97 .. 2.52]
2	2,6	[3.05 .. 3.32]
3	7,7	[4.18 .. 4.41]
4	4	[4.99 .. 5.29]
5	10,12,11	[7.37 .. 7.48]
6	19,13	[7.59 .. 7.73]
7	19,21	[7.84 .. 7.94]
8	18,22	[8.08 .. 8.29]
9	27	[11.08 .. 11.45]

No.	(ppm)	Value	Absolute Value
1	[11.04 .. 11.54]	0.782	8.02771e+0
2	[4.96 .. 5.37]	0.861	8.83605e+0
3	[8.05 .. 8.30]	1.734	1.78031e+1
4	[7.79 .. 7.99]	1.764	1.81115e+1
5	[4.03 .. 4.51]	1.840	1.88853e+1
6	[7.58 .. 7.76]	1.857	1.90614e+1
7	[7.24 .. 7.55]	2.790	2.86436e+1
8	[2.97 .. 3.35]	3.400	3.49020e+1
9	[1.93 .. 2.38]	3.533	3.62708e+1

Fig. S36. ^{1}H NMR spectra of compound **8** (in DMSO)

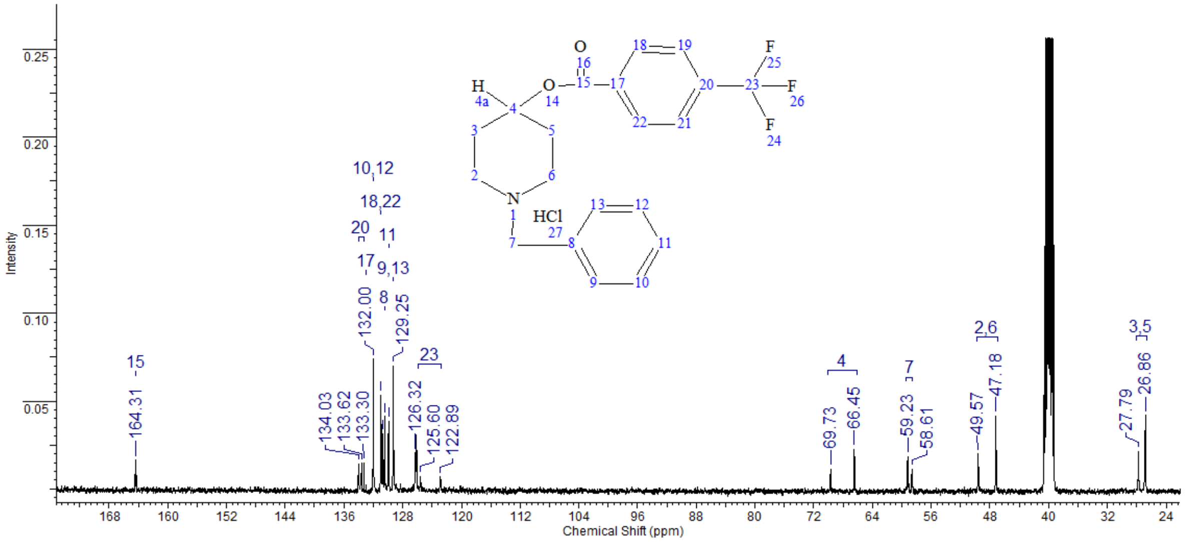

No.	(ppm)	(Hz)	Height
1	26.86	2700.1	0.0419
2	27.79	2793.1	0.0215
3	47.18	4742.6	0.0415
4	49.57	4983.2	0.0197
5	58.61	5891.8	0.0113
6	59.23	5954.1	0.0185
7	66.45	6679.7	0.0224
8	69.73	7009.4	0.0112
9	122.89	12353.8	0.0069
10	125.60	12626.0	0.0073
11	126.32	12697.9	0.0206

No.	(ppm)	(Hz)	Height
12	129.25	12993.1	0.0699
13	129.93	13061.2	0.0387
14	130.42	13111.0	0.0412
15	130.68	13136.9	0.0314
16	130.97	13165.6	0.0532
17	132.00	13269.1	0.0739
18	133.30	13400.5	0.0148
19	133.62	13432.1	0.0145
20	134.03	13473.3	0.0141
21	164.31	16517.4	0.0164

No.	Annotation	(ppm)
1	3,5	[26.64 .. 28.06]
2	2,6	[46.98 .. 49.84]
3	7	[58.52 .. 59.48]
4	4	[66.13 .. 70.06]
5	23	[122.75 .. 125.86]
6	9, 13	129.25
7	11	129.93
8	8	130.42
9	18,22	130.97
10	10,12	[131.96 .. 131.99]
11	17	132.99
12	20	[133.26 .. 133.99]
13	15	164.31

Fig. S37. ^{13}C NMR spectra of compound **8** (in DMSO)

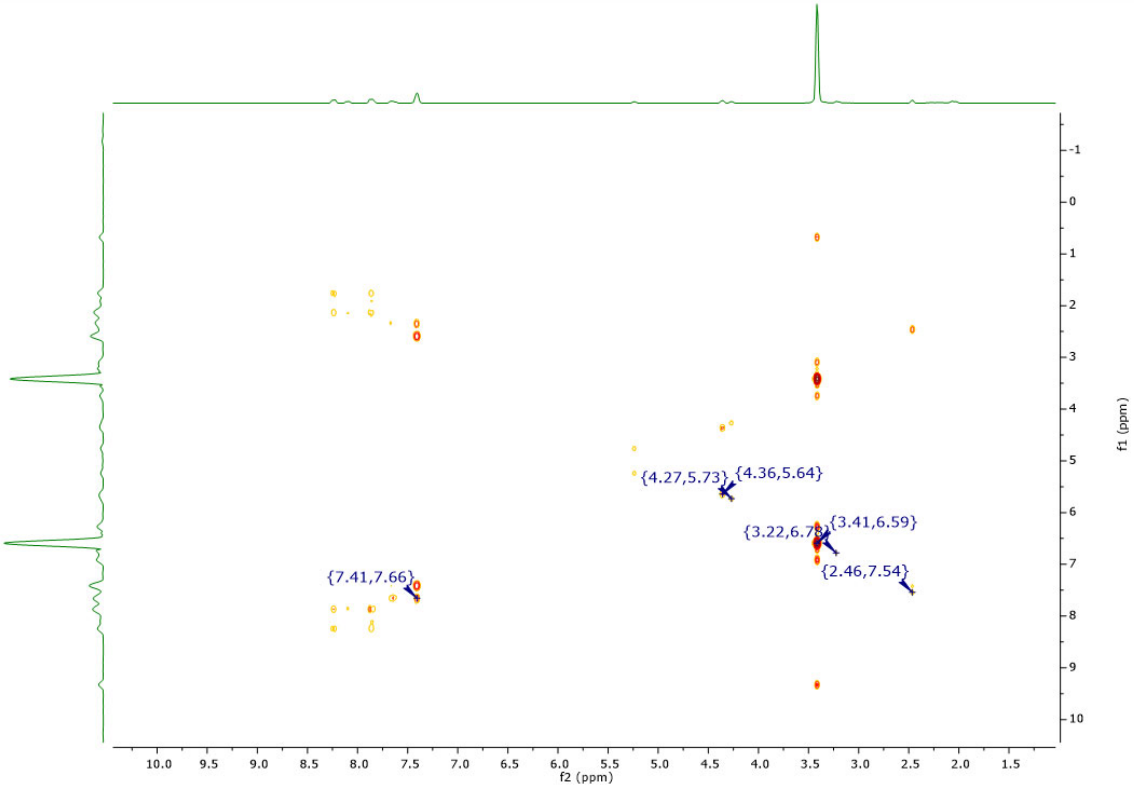

Fig. S38. COSY of compound **8** (in DMSO)

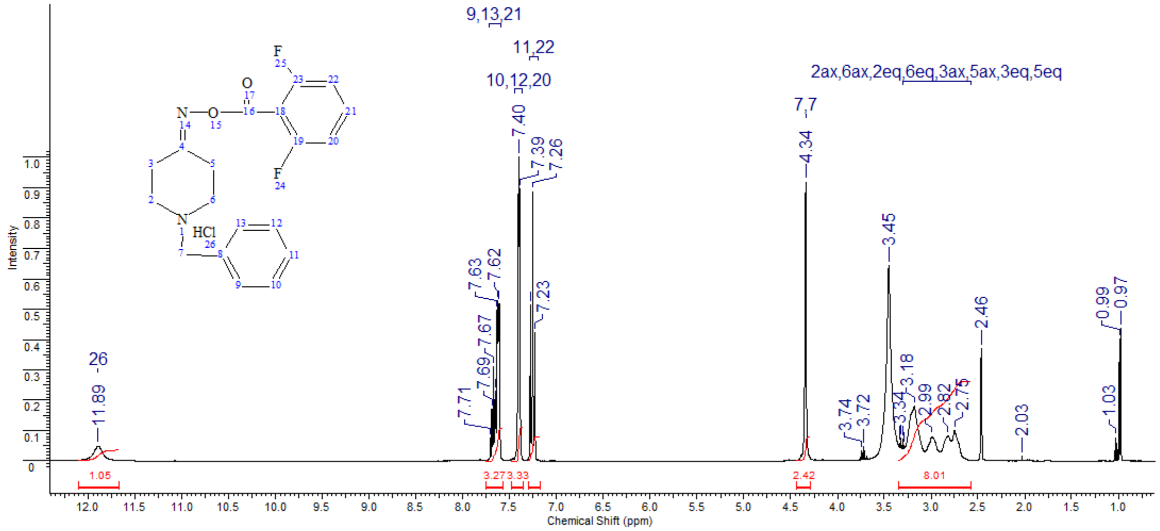

No.	(ppm)	(Hz)	Height
1	0.97	389.7	0.4352
2	0.99	395.6	0.4001
3	1.03	409.8	0.0766
4	2.03	812.4	0.0140
5	2.46	985.0	0.3692
6	2.75	1098.1	0.0988
7	2.82	1127.4	0.0816
8	2.99	1195.7	0.0792
9	3.18	1271.2	0.1787
10	3.29	1313.8	0.0504
11	3.30	1321.1	0.0704
12	3.32	1328.0	0.0747

No.	(ppm)	(Hz)	Height
13	3.34	1334.9	0.0556
14	3.45	1379.3	0.6422
15	3.72	1488.3	0.0456
16	3.74	1494.3	0.0324
17	4.34	1735.6	0.9183
18	7.23	2892.4	0.4219
19	7.26	2901.1	0.8872
20	7.28	2909.3	0.5128
21	7.39	2956.0	0.8957
22	7.40	2958.8	1.0000
23	7.41	2962.0	0.8809
24	7.61	3042.1	0.5170

No.	(ppm)	(Hz)	Height
25	7.62	3044.9	0.5182
26	7.62	3048.1	0.5280
27	7.63	3051.7	0.4955
28	7.65	3058.1	0.2262
29	7.65	3060.0	0.1876
30	7.67	3066.8	0.3095
31	7.69	3073.3	0.1695
32	7.69	3075.1	0.1694
33	7.71	3081.5	0.0750
34	11.89	4754.8	0.0474

No.	Annotation	(ppm)
1	2ax,6ax,2eq,6eq,3ax,5ax,3eq,5eq	[2.58 .. 3.30]
2	7,7	4.34
3	11,22	[7.20 .. 7.29]
4	10,12,20	[7.36 .. 7.44]
5	9,13,21	[7.59 .. 7.72]
6	26	11.89

No.	(ppm)	Value	Absolute Value
1	[11.67 .. 12.10]	1.049	1.09295e+1
2	[4.29 .. 4.44]	2.418	2.51969e+1
3	[7.17 .. 7.30]	2.435	2.53728e+1
4	[7.57 .. 7.76]	3.272	3.40865e+1
5	[7.35 .. 7.48]	3.332	3.47146e+1
6	[2.58 .. 3.34]	8.006	8.34142e+1

Fig. S39. ^{1}H NMR spectra of compound **11** (in DMSO)

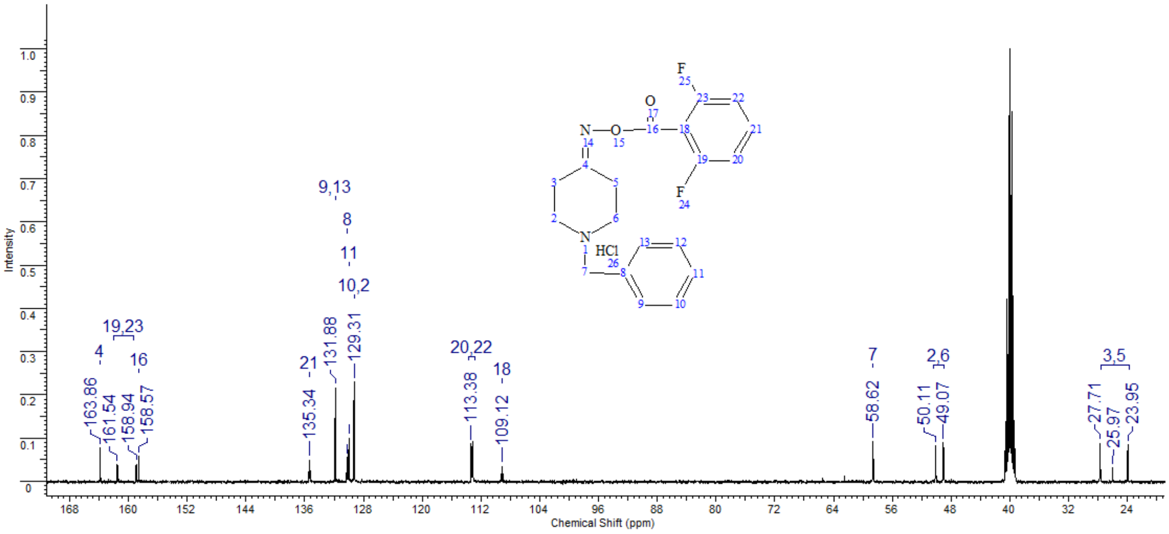

No.	(ppm)	(Hz)	Height
1	23.95	2407.8	0.0846
2	25.97	2611.0	0.0303
3	27.71	2785.4	0.0876
4	49.07	4932.4	0.0889
5	50.11	5036.9	0.0822
6	58.62	5892.8	0.0909
7	109.12	10969.8	0.0348
8	113.38	11397.3	0.0874
9	129.31	12998.9	0.2289

No.	(ppm)	(Hz)	Height
10	130.01	13068.8	0.0996
11	130.25	13093.7	0.0550
12	131.88	13257.6	0.2158
13	135.34	13605.6	0.0495
14	158.57	15940.4	0.0576
15	158.94	15977.8	0.0398
16	161.54	16238.5	0.0378
17	163.86	16472.3	0.0772

No.	Annotation	(ppm)
1	3,5	[23.70 .. 27.64]
2	2,6	[48.94 .. 50.45]
3	7	58.62
4	18	109.12
5	20,22	[112.84 .. 113.65]
6	10,2	129.31
7	11	130.01
8	8	130.25
9	9,13	131.88
10	21	135.34
11	16	158.57
12	19,23	[159.27 .. 162.05]
13	4	163.86

Fig. S40. ^{13}C NMR spectra of compound **11** (in DMSO)

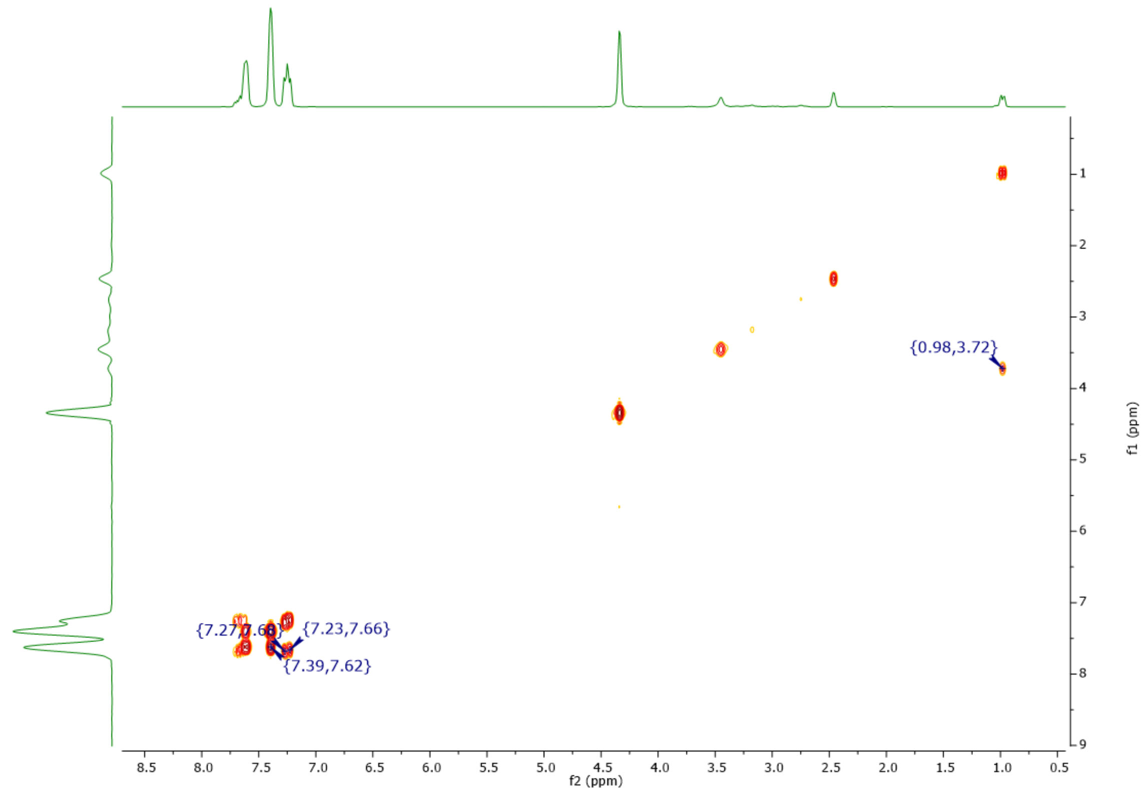

Fig. S41. COSY of compound **11** (in DMSO)

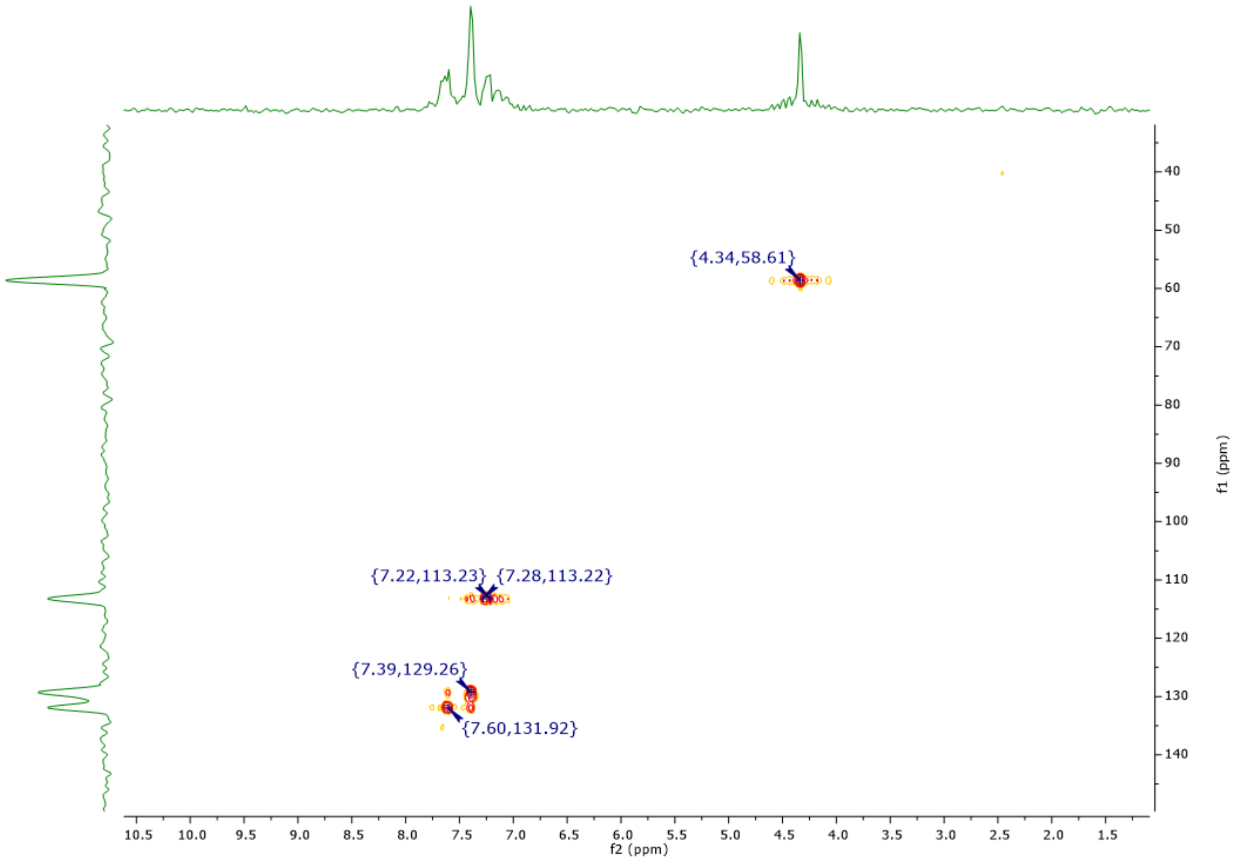

Fig. S42. HMQC of compound **11** (in DMSO)
